# MEA-ToolBox: an Open Source Toolbox for Standardized Analysis of Multi-Electrode Array Data

**DOI:** 10.1007/s12021-022-09591-6

**Published:** 2022-06-09

**Authors:** Michel Hu, Monica Frega, Else A. Tolner, A. M. J. M. van den Maagdenberg, J. P. Frimat, Joost le Feber

**Affiliations:** 1 Department of Human Genetics, Leiden University Medical Centre, Leiden, the Netherlands; 2 Department of Neurology, Leiden University Medical Centre, Leiden, the Netherlands; 3 Department of Clinical Neurophysiology, University of Twente, Enschede, the Netherlands

**Keywords:** Electrophysiology, Data visualization, Burst analysis, Multi-electrode array, Neuron, Spike analysis

## Abstract

Functional assessment of in vitro neuronal networks—of relevance for disease modelling and drug testing—can be performed using multi-electrode array (MEA) technology. However, the handling and processing of the large amount of data typically generated in MEA experiments remains a huge hurdle for researchers. Various software packages have been developed to tackle this issue, but to date, most are either not accessible through the links provided by the authors or only tackle parts of the analysis. Here, we present ‘‘*MEA-ToolBox*’’, a free open-source general MEA analytical toolbox that uses a variety of literature-based algorithms to process the data, detect spikes from raw recordings, and extract information at both the single-channel and array-wide network level. *MEA-ToolBox* extracts information about spike trains, burst-related analysis and connectivity metrics without the need of manual intervention. *MEA-ToolBox* is tailored for comparing different sets of measurements and will analyze data from multiple recorded files placed in the same folder sequentially, thus considerably streamlining the analysis pipeline. *MEA-ToolBox* is available with a graphic user interface (GUI) thus eliminating the need for any coding expertise while offering functionality to inspect, explore and post-process the data. As proof-of-concept, *MEA-ToolBox* was tested on earlier-published MEA recordings from neuronal networks derived from human induced pluripotent stem cells (hiPSCs) obtained from healthy subjects and patients with neurodevelopmental disorders. Neuronal networks derived from patient’s hiPSCs showed a clear phenotype compared to those from healthy subjects, demonstrating that the toolbox could extract useful parameters and assess differences between normal and diseased profiles.

**Supplementary Information:**

The online version contains supplementary material available at 10.1007/s12021-022-09591-6.

## Introduction

A multi-electrode array (MEA) is a device containing multiple microelectrodes through which extracellular voltage changes from neuronal networks can be measured. MEAs for in vitro studies typically consist of a dish with tens to hundreds embedded electrodes, to allow multi-site electrophysiological recordings from living tissue slices or dissociated cell cultures. Electrophysiological recordings of neuronal networks derived from human induced pluripotent stem cells (hiPSC) or rodent brain tissue have been successfully assessed by MEA technology (Mossink et al., 2021; Cao et al., 2012, Bateup et al., 2013; Wainger et al., 2014; Bradley et al., 2018; Tukker et al., 2018; Frega et al., 2019). Compared to conventional in vitro neurophysiological recordings with glass or metal electrodes, MEA technology has several advantages including a high throughput and the ability to record activity of hundreds of neurons simultaneously for long periods of time, and offers a wide range of MEA electrode designs. These electrophysiological recordings of neuronal networks can serve as great functional readouts for healthy and diseased conditions^1^. Not only can MEA technology serve as a platform to elucidate neurophysiological signatures of disease, it is also a platform for drug discovery and drug testing (Cao et al., 2012, Bateup et al., 2013; Wainger et al., 2014; Bradley et al., 2018).

This has led to an increase in popularity of the MEA technology for studying disease or recapitulating the in vivo response to drugs (Tukker et al., 2018; Frega et al., 2019).

However, MEA data are often difficult to manage and process. MEA data are typically large in size due to the high sample rates needed for spike detection, large number of electrodes and long or repeated recording times. Most laboratories use commercial MEA recording systems that come with dedicated software (Plexon inc. Software, 2020; Multi Channel Systems MCS GmbH. Software, 2020a; Axion Biosystems and Axis software, 2020). However, even with the use of dedicated software, analyzing MEA data remains difficult because the analysis tools are limited and users cannot extend or modify existing tools. Different open source MEA toolboxes have been created in the past, aiming to address this need (Gelfman et al., 2018; Bologna et al., 2010; Mahmud et al., 2014; Someville et al., 2010; Pastore et al. 2016; Wagenaar et al., 2005; Cui et al., 2008; Yger et al., 2018; Meier et al., 2008; Mahmud et al., 2012; Bongard et al., 2014; Nick et al., 2013; Dastgheyb et al., 2020). Several of the open source toolboxes assume that the user is already knowledgeable with the language the toolbox is programmed in, which often forms a barrier to use that toolbox (Gelfman et al., 2018; Bologna et al., 2010; Wagenaar et al. 2005). Moreover, most open source toolboxes specialize on only a single aspect in the analysis of MEA data. For example, some focus only on analyzing neuronal network connectivity (Pastore et al., 2016) or spike sorting (Yger et al., 2018), others may include a wide range of tools but miss the very relevant feature of spike detection (Gelfman et al., 2018; Dastgheyb et al., 2020). As a consequence, the user needs to switch between toolboxes to gain a full picture of the data, which easily leads to compatibility issues between the different toolboxes. Moreover, in most toolboxes, data files can only be processed one at a time, thus analyzing multiple files in series is not possible. Finally, the analysis pipeline of most of available toolboxes does not allow the more experienced user to adjust parameters or modify the tool.

Therefore, in spite of these great contributions, analyzing MEA data remains a daunting task. To overcome these limitations, we created *MEA-ToolBox*, an open source general toolbox for research purposes that (i) does not require any coding expertise, (ii) combines multiple tools to extract information about spike train metrics, single-channel burst metrics and network metrics, (iii) provides a standardized analysis pipeline that allows processing of multiple files in series, and (iv) offer more experienced users the ability to modify and add to the existing tools. To the best of our knowledge, such a toolbox is not available yet.

*MEA-ToolBox* is a free Matlab-based, open source program with a user-friendly GUI. It is directly compatible with Multi Channel Systems (MCS) MEA recordings from single-well 60-channel and 120-channel as well as both 12 and 24 multiwell formats. Both raw voltage traces or only spike time stamps can be processed (data sets that already have the detected spike stamps without the voltage trace data). *MEA-ToolBox* provides a standardized and automated working environment for efficient data processing and management of multiple MEA data sets. *MEA-ToolBox* was compared to 2 other open source MEA toolboxes to highlight the necessity for an open source MEA toolbox that contains multiple tools to assess spike trains, single-channel bursts and network metrics all in one package without the need for any coding expertise.

## Material and Methods

### Software Implementation

*MEA-ToolBox* was programmed with a user friendly graphical user interface (GUI) (Geiger, 2019) in the environment of MATLAB 2018b (Mathwork Inc., USA) using GUIDE and is provided as stand-alone application without the need of a MATLAB license, making it freely accessible.

(https://github.com/mhyhu/Toolbox). This allows users without coding expertise to analyze MEA datasets. However, experienced users can also access the source code of the *MEA-ToolBox* to support the development of additional features or modify existing ones. The manual offers a more detailed explanation of the code, starting from page 30.

**Fig. 1:**
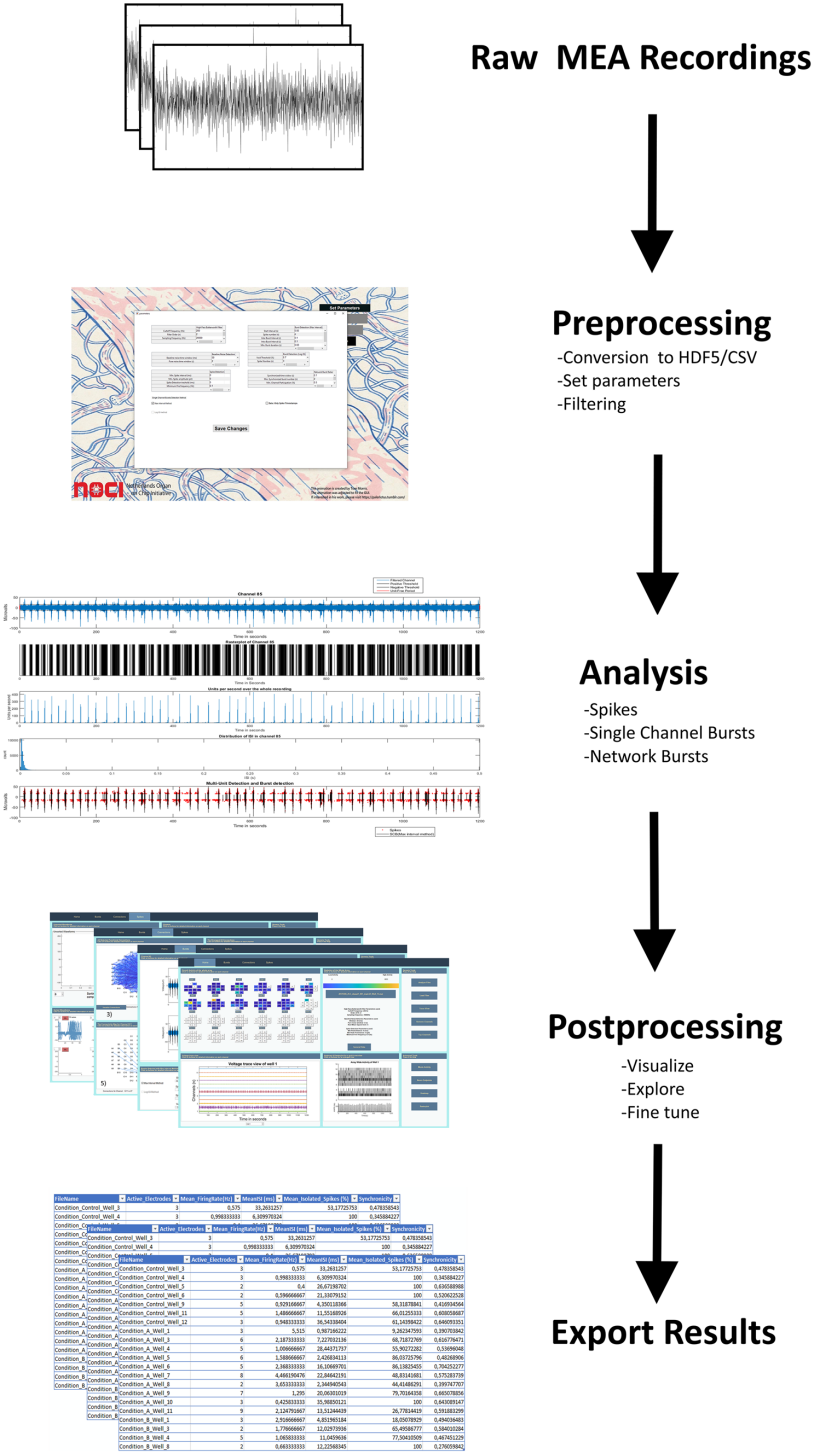
Flow chart of the *MEA-ToolBox*. Raw MEA data files need to be converted to HDF5 format, and are processed by *MEA-ToolBox* for spike detection and spike threshold setting. Spike train metrics are then collected along with burst, network, and connectivity features, quantified as 20 neuro endpoints. The average analysis time was 30 min for a 10 min measurements with 288 electrodes (24 multiwell, MCS) 4 min for spike detection, 7 min for single-burst detection and 18 min for network related metrics. Then the results are visualized in a GUI with the option for post processing if necessary. When satisfied the results can be exported in Excel format

*MEA-ToolBox* analyzes the data in three major steps: spike analysis, burst analysis, and connectivity analysis. This provides a more complete collection of analytic tools compared to existing MEA analysis software, which often lack one of these major steps. Figure 1 depicts a flow chart to explain the different steps followed by *MEA-ToolBox.*

In order to improve standardizing the analysis of MEA data, *MEA-ToolBox* is compatible with the HDF5 file format that is obtained by using the HDF5 file converter from multi-channel systems (Multi Channel Systems MCS GmbH, 2020b). This file format was chosen as it supports storing large amounts of data of different types in a compressed manner (Folk et al., 2011). The toolbox can analyze continuous voltage traces, as well as data sets with only spike time stamps obtained by other software. If the data files were not converted with the multi-channel systems converter then the user must convert their data files into the HDF5 file format according to specifications mentioned in the manual to make it compatible with the toolbox. In addition, *MEA-ToolBox* has also been made compatible with CSV files based on the 24 multiwell format from Axion Biosystems. We provide two different input file formats for the *MEA-ToolBox* as a starting point (MCS and Axion Biosystems). In the case that the data is not a HDF5 or CSV file then we have provided a specific description in the manual of how the CSV file or HDF5 should be structured to make it compatible with *MEA-ToolBox* (See Supplementary Figs. 3 and 4).

In addition, *MEA-ToolBox* is compatible with standard single-well MEA layouts with 60 and 120 channels or the 12 and 24 multiwell standard layouts with 12 electrodes per well. Furthermore, there is an option for multiwell data to combine the analyzed data from multiple wells that belong to the same group.

Lastly, 20 ‘’neuro endpoints’’ (Supplementary Table 1, also found in the manual) are extracted during the analysis that provide 20 different readout parameters concerning spike train, single-channel burst and network metrics, which allows the user to classify the MEA data into different electrophysiological profiles.

### Spike Detection

The spike detection method implemented in *MEA-ToolBox* is based on a published spike detection algorithm with slight alterations (Nick et al., 2013). In short, the spike detection algorithm first detects the baseline noise level in the signal by searching for ‘’spike-free’’ periods within the signal. The signal is split into 50-ms time windows. Afterwards the data within each time window is fitted with a Gaussian distribution. In the original method, if the gaussian fit within a time window had a standard deviation (SD) of 5 or lower, it was interpreted as ‘’pure, spike-free noise’’.

We deviated from this harsh threshold by setting the threshold based on actual MEA data. To this end, the threshold was established by first gathering all the SD values of the gaussian fits of each 50-ms time window in a particular MEA channel, from which the median value was calculated. Next, the SD of each gaussian fit in each 50-ms time window is compared to the median value, and if the SD in the time window was lower than the median, the data within that time window was considered ‘‘pure noise’’. The algorithm continues until a time period of 2 s containing multiple time windows consecutively was found that were all considered to be ‘pure noise’. Afterwards, this process was repeated to find another time period of 2 s that was interpreted as ‘‘pure noise’’. The average of these two time periods is set as ‘’baseline noise’’. Based on the detected baseline noise, the root means square (RMS) is determined, and the threshold to detect spikes is set at 5X RMS. If the algorithm can only find one time window, it will use this time window as ‘’baseline noise’’ however when the algorithm cannot find any window then the channel is determined to be too noisy and disregarded for further analyses.

Spike detection is performed for each individual MEA channel separately thus accounting for differences between channels. After the spikes are detected, ‘’artifact detection’’, by inspection of spike waveforms is performed (Wagenaar et al., 2005). The detected spikes are validated through an artifact detection algorithm where the detected spike has to have the highest peak with no secondary peaks within a ± 1 ms window, and more than 50% of the spike amplitude falling within this same window (Wagenaar et al., 2005). If the detected spikes do not fulfil this criteria they will be removed from further analyses. Furthermore, one can also set a minimum voltage that the amplitude of the spikes should reach before considering them as spikes (default = 0 µV). after the spikes are detected the a final check will be performed to determine if a channel is active or not. The channel is considered active when the firing rate is at least 0.1 Hz otherwise the channel will be disregarded from further analyses.

**Fig. 2:**
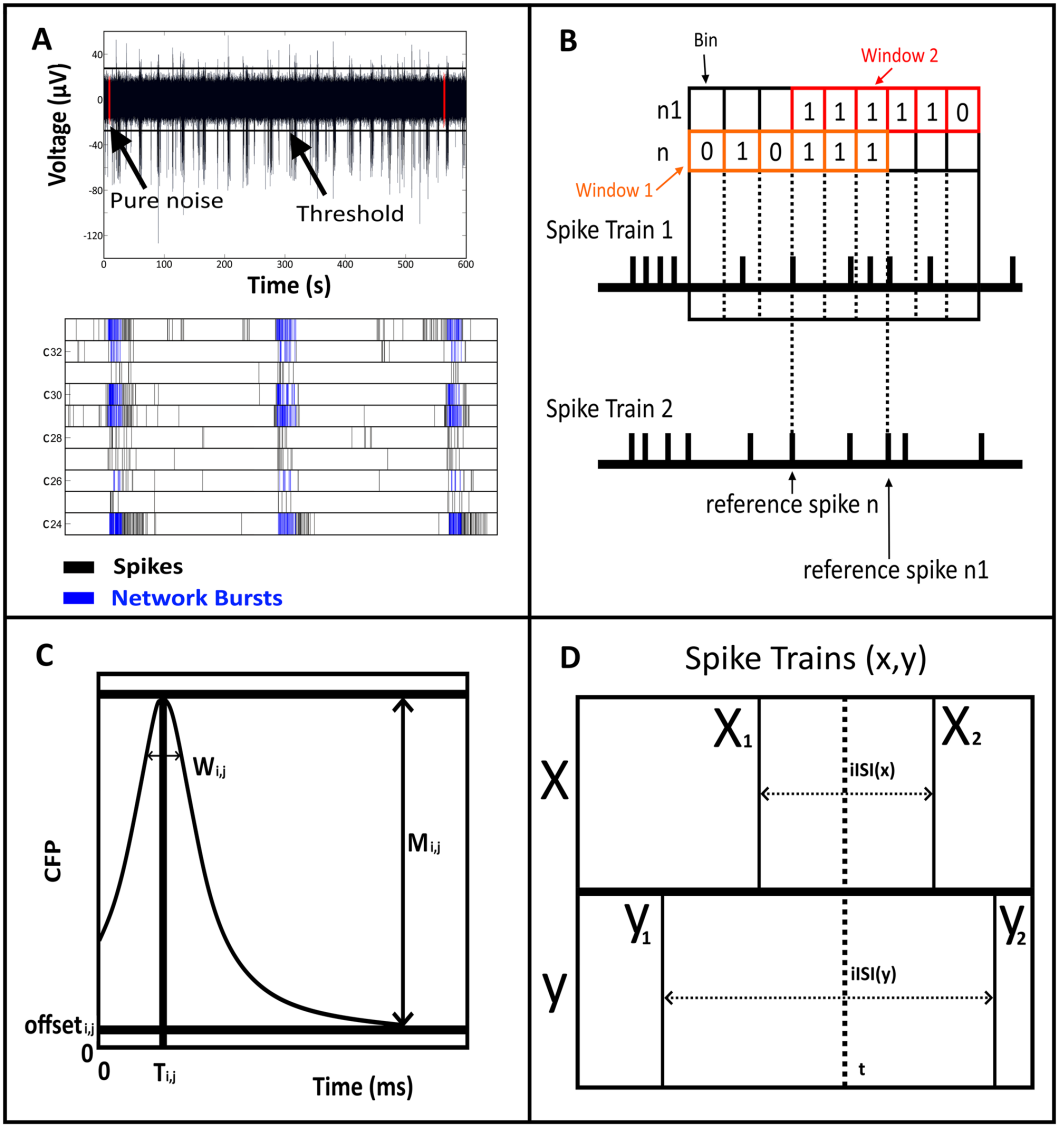
Detection of spikes, single-channel and array-wide network bursts and assessment of cross-correlation, conditional firing probability and inter-spike intervals. Spikes are detected based on a threshold of 5X the root mean square (rms) of baseline noise. **A**, top: the time windows used for assessing baseline noise are indicated in red and the threshold based on the baseline noise is indicated by black horizontal lines. **A**, bottom: array-wide network bursts are detected based on the previously assessed single-channel bursts, which are indicated as black lines for the visualized six channels C1 -C6. The first step in detecting array-wide network bursts is to find single-channel bursts that occur within a 100-ms time window from each other across all the channels based on their first spike. These bursts are ‘‘combined’’ and are called network bursts. After detecting network bursts, the beginning and end times of these synchronized bursts are based on the earliest spike time and latest spike time of the SCBs that comprise the synchronized bursts. Then any SCBs that occur within the time window of the synchronized bursts are added to the respective synchronized bursts. Lastly, a check is performed to determine if a minimum of 25% of the active channels are participating in these synchronized bursts before considering it a network burst. In the example given in A, three network bursts in blue are found because the three synchronized bursts and more than 25% of the active channels are contributing to each network burst. **B**. Cross correlation is assessed by comparing the spike train output of two channels. For this, the spike time stamps of the two channels are vertically aligned with each other, whereby one channel serves as the reference (spike train 2) and the other channel is the target (spike train 1). A time window is centred around each spike in train 2 and subsequently split in smaller bins, whereby the toolbox determines whether any spikes from spike train 1 fall within each bin of the time window. **C**: Simplified representation of the function used to calculate the conditional firing probability (CFP) for an electrode pair i and j (see Materials and Methods Eq. 2). M_i,j_ represents the maximum probability above offset, whereas T_i,j_ is the time delay at which the CFP is maximum. The w_i,j_ is the width at offset_i,j_ + 0.8 M_i,j_ and the offset_i,j_ represents uncorrelated background activity. **D**: The ISI distance calculated at time t requires three inputs: the time stamps of the previous spike (X1), the time stamp of the following spike (X2). Using the time stamps X1 and X2, the instantaneous inter-spike interval (iISI) can be calculated which is the third input. This process is also repeated for spike train Y after which the iISIx and iISIy are used to calculate a ratio between the iISI of both spike trains, which is normalized to a value between -1 and 1 according to Eq. 3 before temporally averaging

An example of the spike detection is shown in Fig. 2A (top) where a filtered trace is displayed, in which the ‘‘spike-free’’ periods are displayed in red. Based on these calculated ‘’spike-free’’ periods a threshold was set (black line) on both sides of the y-axis. Any peak that exceeded this threshold was labeled as a spike.

### Single-Channel Burst and Array-Wide Network Burst Detection

A ‘’single-channel burst’’ (SCB) is defined as a collection of consecutive spikes with small inter-spike intervals occurring during a defined relatively short period occurring on a single MEA channel (Zeldenrust et al., 2018). *MEA-ToolBox* uses the ‘‘max interval’’ and ‘’log ISI’’ method (Cotteril et al., 2016) to detect SCBs. These two methods were chosen based on literature (Cotteril et al., 2016).

In short, the max interval method consists of tuning 5 parameters. These parameters include the “Start Interval (s)”, which is the maximum time interval between the first two spikes of a SCB, set at 0.05 s. The number of spikes (“Nspike (n)”) which is set at 3. Inter-burst interval as well as intra-burst interval, with default settings at 0.1 s for both. Inter-burst interval (IBI) refers to the maximum time interval between detected SCBs. If the time interval is smaller than this parameter than the SCBs will be combined. Intra-burst interval (s) is the maximum time interval of the spikes that follow after the two initial spikes are found of a potential SCB. If the time interval is smaller than this value it will be included in the SCB. Finally, “Minimum burst duration (s)” which is set at 0.03 is combined with the above-mentioned parameters to select what the minimum duration of a SCB should be. These parameters are chosen based on values found in literature (Cotteril et al., 2016).

The other method for SCB detection is called the Log ISI method where 2 parameters can be tuned: the previously mentioned ‘’Nspike (n)’’ which is set at 3 as default and the ‘’Void Threshold (%)’’, which is the threshold for determining if the peaks detected in a log ISI histogram are separated well enough. If the calculated value is higher than the set value, the peaks are separated well enough (Pasquale et al., 2010).

When multiple SCBs fire within a similar time window on multiple channels, these are called network bursts (Nb) (Van pelt et al., 2004). The detection method for network bursts in the toolbox is also based on methods from literature (Wagenaar et al., 2005; Mendis et al., 2016). The method is based on first detecting the SCBs on each channel after which the algorithm will use the time of the first spike within each SCB to compare with each other to determine which SCBs fire within a 100-ms time window. If at least 2 SCBs are found to fire within a 100-ms time window based on their first spike from each other on different channels these SCBs are considered to represent a synchronized burst. Then the algorithm considers the start time and end time of the synchronized burst to be the earliest spike time point and the last spike time point of the SCBs that are part of the synchronized burst. Next any SCBs that falls within the time window of the synchronized burst are added to the synchronized burst to create a potential network burst. The last step is to remove any potential network bursts in which less than 25% of active channels are contributing to the network burst (Fig. 2A, bottom).

### Connectivity Detection

*MEA-ToolBox* provides an estimate of functional connectivity of neuronal networks based on cross correlation (CC). The CC-based method that is used in the toolbox is termed conditional firing probability (CFP) (Le Feber et al., 2007), and is established based on co-occurrence of spikes across the different MEA channels (Mendis et al., 2016). In short, data is split into chunks of 2 (Pastore et al., 2016) spikes summed over all channels, and in all chunks for each pair of active electrodes (i, j) a CFP curve is calculated as the probability that an action potential is recorded at electrode j at t = tau (tau < 500 ms), given that one was recorded at electrode i at t = 0. To estimate this probability, for each 0.5-ms time bin the number of action potentials at electrode j is counted that follows the action potential at electrode i (N_followi,j_). This is divided by the number of action potentials at electrode I, N_i_, as indicated by the following Eq. 1. The X_i_ and X_j_ are binary arrays that represent the recorded signals at electrodes I and j as point processes. X = 1 represents an action potential and otherwise it is 0.

1 $${CFP}_{i,j}\left[\tau\right]=\frac{N_{follow{}_{i,j}}}{N_i}=\frac{{\sum_t\;X}_i\;\lbrack t\rbrack\;\cdot\;X_j\;\;\lbrack t+\tau\rbrack}{{\sum_t\;X}_i\;\lbrack t\rbrack}\left|0\;<\;\tau\;\leq\;500\;ms.\right.$$

To facilitate interpretation, a standard function was fitted to CFP curves using a Nelder-Mead simplex algorithm to reduce the mean squared error, as shown in Eq. 2:

2 $${CFP}_{i,j}^{fit}\left[\tau \right]=\frac{{M}_{i,j}}{1+{\left(\frac{\tau -{T}_{i,j}}{{w}_{i,j}}\right)}^{2}}+{offset}_{i,j}$$

In Eq. 2, M_i,j_ is the maximum value of the CFP_fit_ above the offset, Ti,j the latency at which maximum CFP is reached, wi,j is a parameter related to the width of the peak and offset_i,j_ represents uncorrelated background activity (see Fig. 2C). M_i,j_ is interpreted as the strength of a connection, T_i,j_ as the latency. Connectivity maps were made based on the averaged M_i,j_ values over all the chunks obtained from the CFPfit.

### Spike Train Synchronicity Measurements

Spike train synchronicity can be defined as the similarity between pairs of spike trains. We implemented the ISI-distance method to calculate the spike train synchronicity due to its parameter-free nature and flexibility to be extended (Kreuz et al., 2007; Satuvuori et al., 2017). The method transforms the spike times of two spike trains into temporal profiles with one value for each time point. To obtain this value, the time stamp of the previous spike and time stamp of the following spike are used to calculate the instantaneous inter spike interval. For example, Fig. 2D shows two spike trains X and Y with two spikes in both. At time t, the last spike with time stamp < = t (X1 and Y1) and the first spike with time stamp > t (X2 and Y2) are determined for both spike trains.

The instantaneous inter-spike interval (iISI) is calculated as the difference between these time stamps (iISI_x_ = X2-X1 and iISI_y_ = Y2-Y1). Then a normalized ratio value I(t) is obtained by dividing the two iISI values from both spike trains according to the following Eq. 3:

3 $$I\left(t\right)=\left\{\begin{array}{cc}{iISI}^{\left(x\right)}/{iISI}^{\left(y\right)}-1&if\;{iISI}^{(x)}\leq{\;iISI}^{(y)}\\-({iISI}^{\left(y\right)}/{iISI}^{\left(x\right)}-1)&otherwise.\end{array}\right.$$

This process is repeated for all the spike times in the whole signal. The last step involves temporal averaging across the absolute values of I to get the mean ISI distance. The result is a value between -1 and 1. Negative values indicate that the first spike train is faster than the second, positive values the opposite. I(t) = 0 means that both trains are equally fast.

### Spike Sorting

Due to the use of the MEA, it is important to realize that the recorded signals may contain action potentials from multiple neurons as the extracellular electrodes are typically bigger than a neuron. Therefore, in order to investigate the contributions of individual neurons it is necessary to separate the detected spikes from different neurons on the same electrode. This is possible using a method called spike sorting. Spike sorting is a clustering process by which detected spikes are separated into different clusters based on the similarity of their waveforms. Each detected cluster is a different neuron because the extracellular spike waveform is different due to the biological properties of neurons. One of the key steps in spike sorting is to determine which features of the spike waveform should be used for the clustering process without any manual intervention.

We adapted a method from the literature that is based on combining different feature extraction techniques with Gaussian mixture models (GMM) (Souza et al., 2019). This method was shown to be better than other approaches known in literature with the added benefit of needing no manual intervention. GMMs are a type of statistical model that fit data using Gaussian distributions.

To better fit complex MEA data that contain multiple peaks/features rather than a single type, multiple Gaussians distributions each accounting for a distinct peak/feature are combined to form this ‘’continuous’’ Gaussian curve (mixture) to better fit complex data. In this recent method, GMMs are used in combination with either a principal component analysis (PCA), weighted PCA or a wavelet decomposition (WD) to extract three features from the data that are dependent on the GMM (peaks, inflection point of the mixture and the distance between individual Gaussians). A feature that is independent of the GMM (variance of the features) is also used. The five highest-ranked PC scores or wavelet coefficients were selected for the clustering procedure. The first step in the clustering procedure is to overestimate the number of clusters by fitting a GMM into the five selected features with a high number of Gaussians. As a final step, an additional GMM was fitted on the previously detected cluster centers with each Gaussian corresponding to a detected cluster center. Afterwards each waveform was assigned to the cluster that had the highest corresponding probability.

We adopted the Souza et al. (2019) user-friendly interface into *MEA-ToolBox* to allow the user to not only visualize the clusters but also to check for stability of the clusters and to remove clusters if needed. The stability of the clusters is visualized as the peak-to-valley distance of the waveforms of a cluster together with the firing rate over the whole recording.

This can be used to determine if the cluster has similar properties over time and to potentially identity shifts of a neuron from a cluster to another cluster. Therefore, this feature allows the user to get a better understanding of their dataset.

### MEA-ToolBox Analysis Parameters Summary

A summary of the main *MEA-ToolBox* default parameters that are used to run the standard analysis pipeline are summarized here and can be accessed before starting the analysis procedure from the *MEA-ToolBox* GUI, as shown in Supplementary Fig. 1. All of the mentioned parameters below are changeable by the user, however we recommend to use the default values, as seen in brackets. These parameters include: (1) the high-pass Butterworth filter cutoff frequency and filter order (200 Hz and 2), (2) baseline noise detection and pure noise window (50 ms and 2 s), (3) spike detection threshold, spike detection interval, minimum spike amplitude and minimum fire frequency to be considered an active channel (5, 0 ms, 0 µV and 0.1 Hz), (4) Burst detection with Max Interval Method start interval, number of spikes, inter burst interval, Intra burst interval and minimum burst duration (0.05 s, 4, 0.1 s, 0.1 s and 0.03 s), (5) Log ISI Method with void threshold and number of spikes (70% and 3), and (6) network burst detection synchronized time window, minimum synchronized burst count and minimum active channel participation (0.1 s, 2 and 25%). An option to analyze spike time stamps only is also provided. Once these parameters are set, *MEA-ToolBox* can analyze multiple files in series which means that entire experimental recording data sets can be processed instead of having to run the analysis file by file with the same parameters.

### Data Sets Used for Developing MEA-Toolbox and Application of MEA-Toolbox to Experimental Data

We used a model data set obtained from a patient with Kleefstra syndrome to compare the performance of *MEA-ToolBox* to two other MEA toolboxes: *meaRtools* (Gelfman et al., 2018) and *Multiwell Analyzer* (Multi Channel Systems MCS GmbH, 2019). These toolboxes were the only ones compatible with the multiwell format. To complement comparisons, we also show the usefulness of having the tools to sort spikes and perform a connectivity analysis all in one package and state the limitations of *MEA-ToolBox* compared to the other toolboxes. To perform a comprehensive analysis with *meaRtools* and *Multiwell Analyzer*, the burst detection method were set to match the *MEA-ToolBox* parameters used for single-channel burst detection and array-wide network burst detection. Since network burst inter burst interval (IBI) and network burst coefficient of variation (CV) of IBI are not included in *meaRtools*, these had to be manually calculated, by using the ‘’Network Burst (Nb)_start’’ and ‘’Nb_end’’ values located in the variable nb, all separately for each well; the endpoint, called ‘’mean_NB_time_per_sec_10’’, was used to represent the duration of the network burst detected by meaRtools. For *Multiwell Analyzer*, the network burst IBI and network burst CV of IBI were manually calculated using the start and end times of the detected network bursts.

All graphs seen in Fig. 5 were created by following a similar approach in which the average was taken of the same four wells for each endpoints. All three toolboxes were used on a PC with Windows 10, with an intel Xeon E3-1240 v6 clocked at 3.7 GHz and 16 GB of Ram.

## Results

### Data Visualization and Data Analysis with *MEA-ToolBox*

**Fig. 3:**
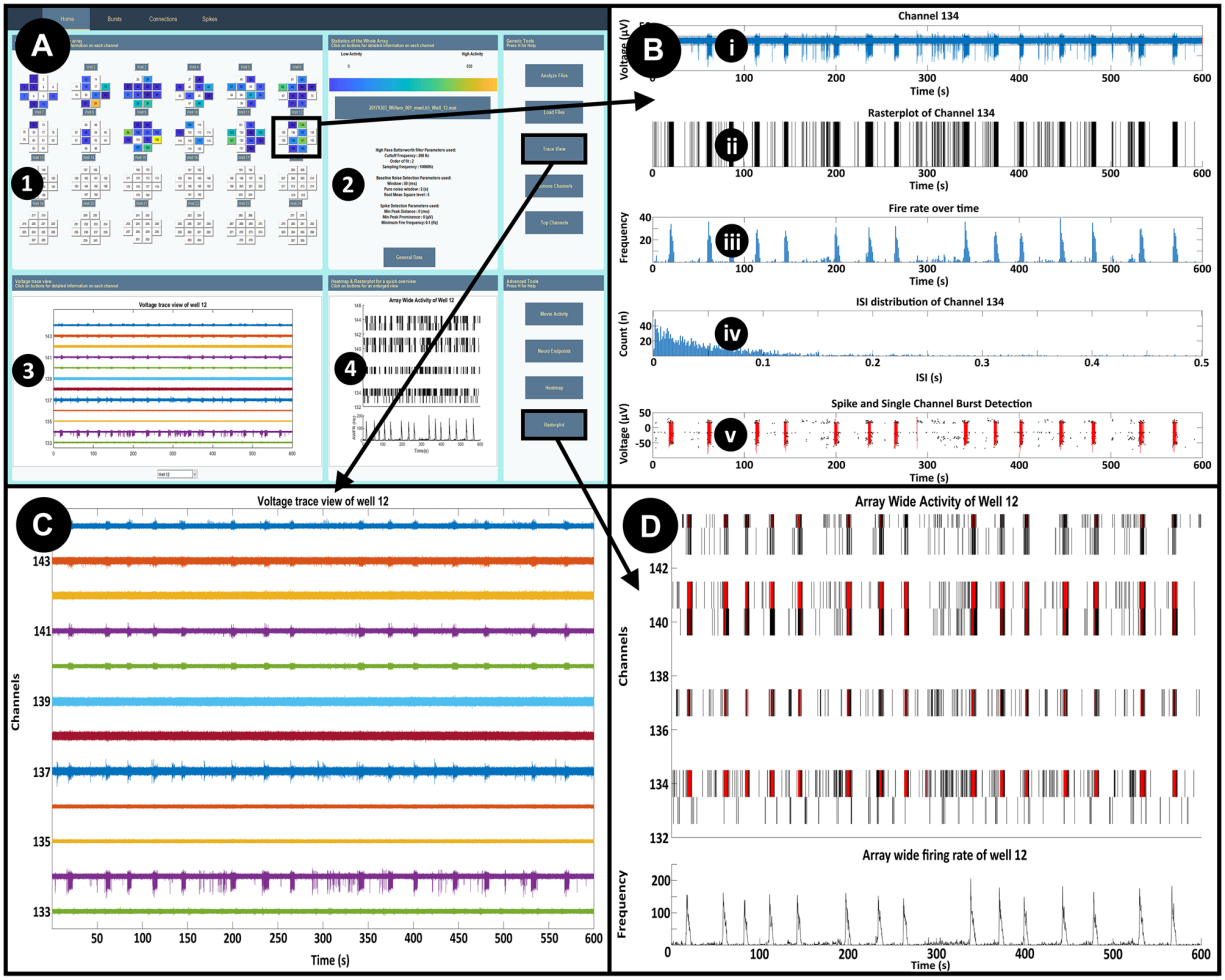
Home screen display. Color coded overview of array activity **A1**, parameters used for the analysis **A2**, full trace visualization **A3** and raster plot **A4**. Single-channel metrics **B**, filtered signals **C** and raster plots with detected bursts (red) and network bursts(if applicable, blue) **D** are also displayed. For multiwell formats, selecting a well in the overview will automatically display the accompanying trace view and raster plot of the selected well. Well 12 is shown here as an example with channel 134 selected for single-channel metrics **B** containing information about **i** the filtered voltage trace showing which part of the measurement is used as baseline noise to set the threshold for spike detection (pure noise, red) together with the thresholds used for spike detection (horizontal black bars), **ii** a spike raster plot of a single-channel, **iii** A firing rate histogram for the selected channel, **iv** Histogram of the distribution of the inter spike interval (ISI) and **v** Time stamp of detected spikes (black) and bursts (red)

The user can change several parameters before starting the analysis (Fig. 1 suppl). Once the analysis is finished the toolbox will save a.mat file that contains all the information the toolbox needs to calculate the neural endpoints and allow use of the GUI. For a more detailed look at the output.mat file please see the manual. *MEA-ToolBox* is programmed with a user-friendly GUI home screen (Fig. 3), which is designed to give an overview of the MEA data. For this, the home screen tab shows a color-coded overview of the activity of all channels on the left top (Fig. 3A (1)). Next, several options are available to visually inspect the analyzed data and modify the default parameters to suit the user’s need. For example, each channel can be accessed for additional single-channel information (Fig. 3B, e.g.: channel 134). Five parameters are displayed: (1) filtered voltage (noise-estimation period indicated in red) together with the thresholds used for spike detection (horizontal black bars); (2) spike raster plot of the selected channel; (3) a firing rate histogram of the selected channel; (4) histogram of the inter-spike-interval (ISI); and (5) time stamps of detected spikes (black) and bursts (red). A table is displayed showing information about the file name and the parameters that will be used in the analysis (Fig. 3A (2)). A general data button is provided that will retrieve all spike train metrics and related statistics for every single-channel in the displayed file. These include spike number, mean ISI, median ISI, std ISI, SCB (Max Interval), SCB (Log ISI), Fire rate, mean instantaneous firing rate, mean CV ISI, mean CV2 ISI and threshold value. This information is exportable as an Excel table. Raw voltage traces of all the channels within one well can be visualized (Fig. 3A (3)) by using a “full trace” button. An example is shown in Fig. 3C, whereby the raw voltage signals are displayed for a 200-s window.

Following spike detection, a spike raster plot is displayed for a selected single-channel in which each black line represents a spike (Fig. 3A (4)). A “raster plot” button is also provided with a more detailed view of the spike raster plot (Fig. 3D). Here, single-channel bursts can additionally be displayed in red, overlaid with either the spike raster plot or (in blue) array-wide network bursts. A ‘‘10 most active channels option’’ displays a new graph that allows for a quick determination of the most active channels. This option also allows to detect broken or degrading channels where the threshold detection will detect pure noise of high amplitude, resulting in falsely hyperactive channels. *MEA-ToolBox* can remove such channels from further analysis with a ‘’remove channel’’ option. When used the single-channel burst detection, network burst detection and connectivity detection algorithms are rerun again. Heatmaps of the whole MEA are also generated based on the number of spikes in each channel. In addition, an animation of the spike activity over time can be saved as an.avi file. The previously mentioned visualizations can all be saved as a.tiff file or any other image file by going into the upper left corner ‘file’ menu and selecting the save as button. For extraction of MEA spike features, all analyzed files can be placed in a single folder—for example from multiple experiments or from the same MEA over multiple days/weeks)—and via the “Neuro endpoints” button 20 different features of spike activity are calculated for each file and is saved as an excel file with the different neural endpoint in columns and each row represents a well. Supplementary Table 1 provides a detailed description of the Neuro endpoints and their definitions.

In case of multiwell data, *MEA-ToolBox* can group certain wells together to calculate the mean/median and SEM/25%-75 quartiles values of such groups for the 20 Neuro endpoints. The analyzed Neuro endpoint data are exportable as an Excel table for further data processing or creating graphs. If certain wells are grouped, then the rows represent the grouped wells which is also indicated in the naming of the row.

### Burst Analysis with *MEA-ToolBox*

**Fig. 4:**
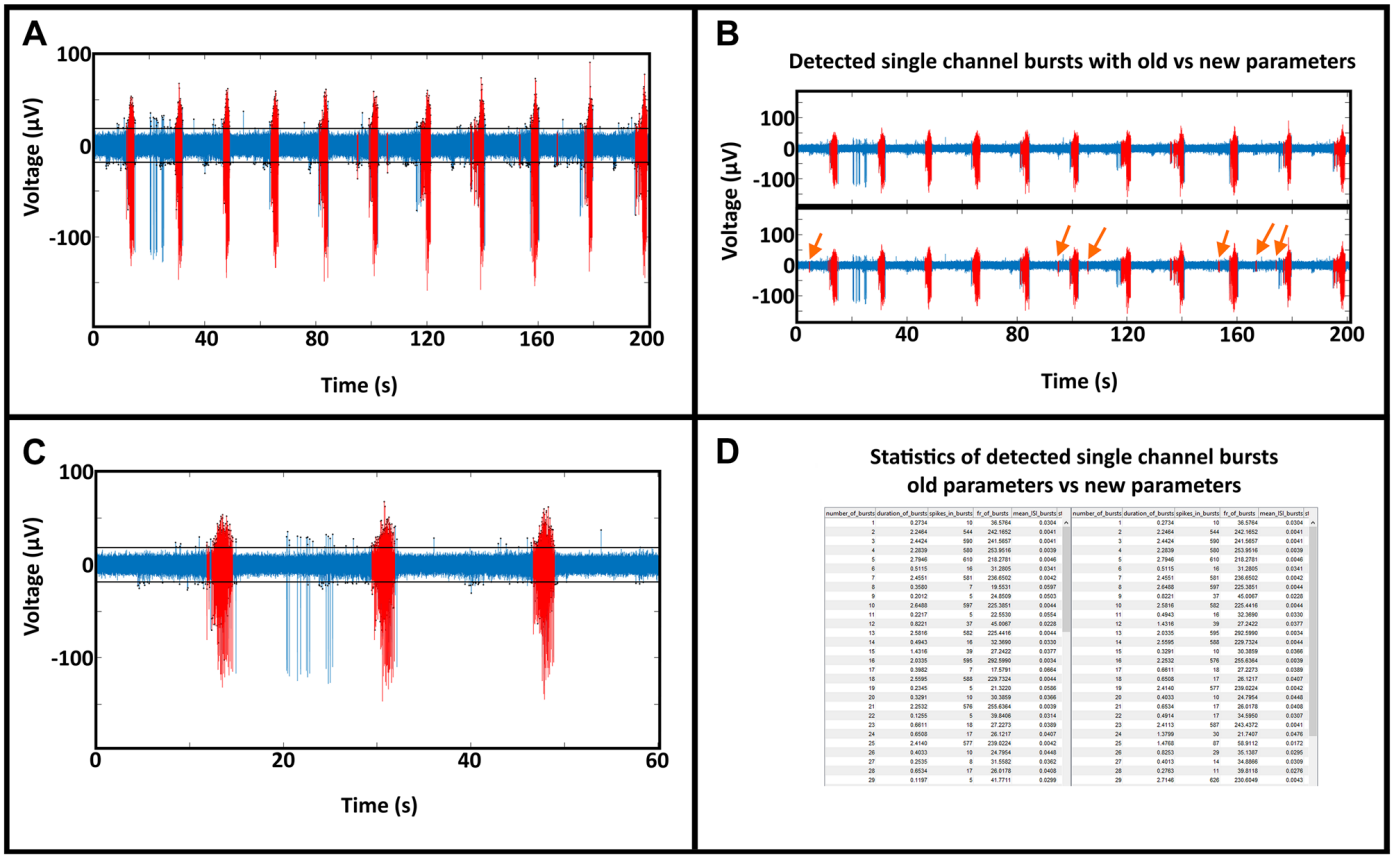
Burst detection display. **A**: An example of a single-channel burst visualization (channel 134, well 12) detected with the max interval method in the Burst tab of the toolbox. The detected SCBs are displayed in red with the spikes and the used threshold to detect the spikes in black. **B**: Within the burst tab it is possible to not only visualize the detected SCBs but also change the parameters used to detect the SCBs if the user doesn’t agree with the detected SCBs. The user can choose between the log-ISI and the max-interval method after which the corresponding parameters with the methods will become available to change. These parameters are located in the bottom of the tab (GUI not shown). For the log ISI method the user can change the void threshold and the minimum spike count to be considered a SCB. The method involves checking if the log ISI histogram detects multiple peaks and if the algorithm detects multiple peaks then the algorithm will check how well they are separated by using the void threshold parameter. The void threshold value indicates how strict the separation between the peaks needs to be before the algorithm considers it a SCB. If the algorithm cannot find two peaks then it is not considered a SCB. For the max interval method the user can change 5 parameters, the maximum start interval between the first 2 spikes, the number of spikes needed to be considered a SCB, the inter-burst interval which refers to the minimum time that needs to be present between the each SCB ( if the time is shorter between detected SCBs then they will be combined), the intra-burst interval which is the time between the spikes after the 2 initial spikes in a SCB ( if spikes after the 2 initial spikes is shorter than this value than they will be included in the SCB) and lastly the minimum burst duration. After the user changed the parameters, the user use the rerun button to only rerun the SCB detection algorithm and when finished the toolbox will display the newly detected SCBs or not. Here the orange arrows indicate the additional burst detected by changing the minimum spike count from 4 to 3. **C**: Possibility to zoom inside the voltage signal to visualize specific bursts. **D**: Statistical report containing all the SCB metrics for the selected channel for the old parameters and new parameters if the user chose to rerun the detection otherwise it will only display the SCB metrics for detected SCBs with the initial parameters at the beginning of the analysis

The start screen and ‘’Bursts’’ tab of *MEA-ToolBox* allows for visualization of detected single-channel (SCB) and network bursts (Nb). Detected spikes, thresholds, SCBs and Nbs can be displayed here (e.g. channel 134, Fig. 4A). Furthermore, the ‘‘Bursts’’ tab also includes options for fine-tuning of the parameters used for SCB detection and Nb detection. The user can also rerun these detection algorithms using the changed parameters and make a comparison between the detected SCBs and Nbs without the need to re-analyze the whole file (Fig. 4). The result is a comparison between the detected bursts using the previous parameters versus the newly chosen parameters (as indicated in black and blue). An example is shown where the logISI parameters for the number of spikes was decreased from 10 to 3 and shows that indeed more bursts are detected (Fig. 4B, arrows). This option can be useful if the burst detection needs to be less strict or fit a more precise profile and therefore can be first visualized here before re-analyzing the whole file again.

Similarly, there is a button for optimizing the array-wide network burst detection parameters. These options include the ability to toggle between using the log ISI detected bursts or the max interval detected bursts (default) and changing the network burst parameters. Zooming in the detected bursts for closer inspection is done by selecting which burst number to visualize, an example of Max Interval burst #2 and #3 is shown in Fig. 4C for channel 134.

Finally, a table is presented that contains all the single-channel/network burst metrics for the selected channel of both the max interval method and log ISI method (Fig. 4D). Metrics for the Max Interval and log ISI detected bursts include number of bursts, duration, number of spikes per burst, frequency of burst, mean ISI in bursts and the percentage of spike participating in bursts. For the network bursts, statistics about the number of network bursts, start time, end time, duration, fire rate of the spikes within the network bursts, the ISI of the spikes within the network bursts and the IBI between network bursts are extracted.

### Connectivity Analysis with *MEA-ToolBox*

The *MEA-ToolBox* connectivity analysis tool provides an estimate on functional connectivity within the neuronal networks recorded on a MEA, based on the conditional firing probability (CFP). This is a cross correlation-based method in which the relationship between two spikes is determined by two variables: M for the strength of and T for the latency of a presumed relationship (Le Feber et al., 2007).

**Fig. 5:**
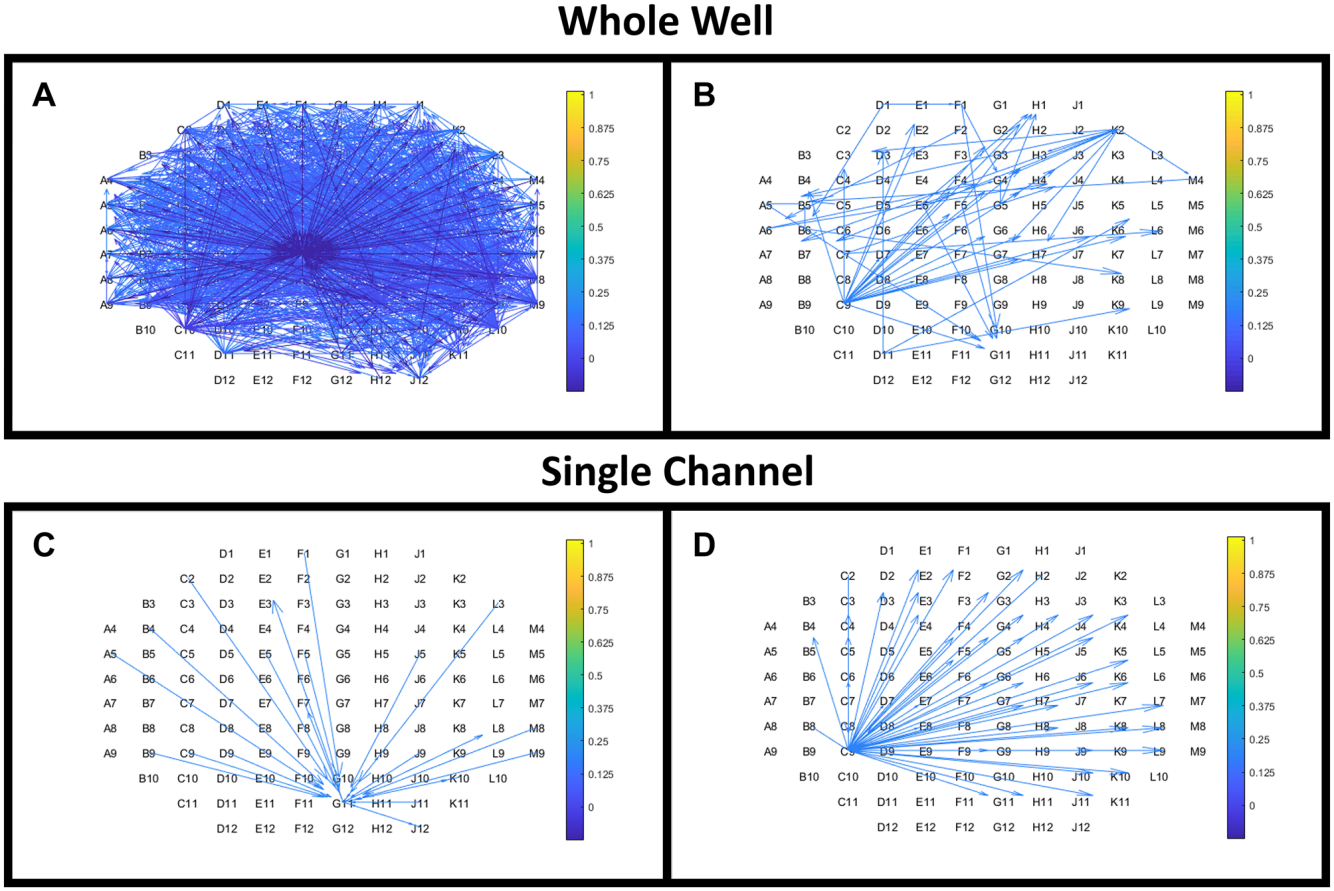
Connectivity map of an example single-well MEA recording. **A**: The human induced pluripotent cell (hiPSC)-derived neuronal culture in this example exhibited a multitude of putative functional connections, shown by the drawn connections, based on the connectivity map. The connectivity map was created based on the M values obtained from the conditioning firing probability for every electrode pair. The M values represent the strength of the connection between an electrode pair. **B**: The top right panel also show the functional connections but will only take into account the strongest 60 connections found. Visualize connections from a specific channel (**C** and **D**)*,* that allows to select a channel and will only visualize the connections associated with this selected channel. Either the selected channel is connected to other channels or other channels are connected to the selected channel. Arrows indicate direction of the putative connections, whereas the color indicates the strength of the connections

Here, we present some example data from human induced pluripotent cell (hiPSC)-derived neuronal cultures for visualization purposes to show that the versatility of the connectivity tool is not restricted to a certain electrode layout. In this example, the data was recorded on a 120-channel single MEA. Figure 5 shows the connectivity analysis tab in which the left panel will show all the detected putative connections, whereas he right panel only shows the 60 strongest connections (Fig. 5A and B). Setting a limit to the number of shown connections improves readability of the graph. The putative connections associated with a selected channel can be visualized in the bottom panels. This will allow for a more detailed look at a specific channel without the interference of the other channels (Fig. 5C and D). The option “display direction” adds arrowheads to drawn lines to indicate the direction of a putative connection. All figures are exportable as.tiff files with 1200 dpi and a resolution of 1920 × 1080 by using the corresponding export figure button.

### Spike Sorting with *MEA-ToolBox*

**Fig. 6:**
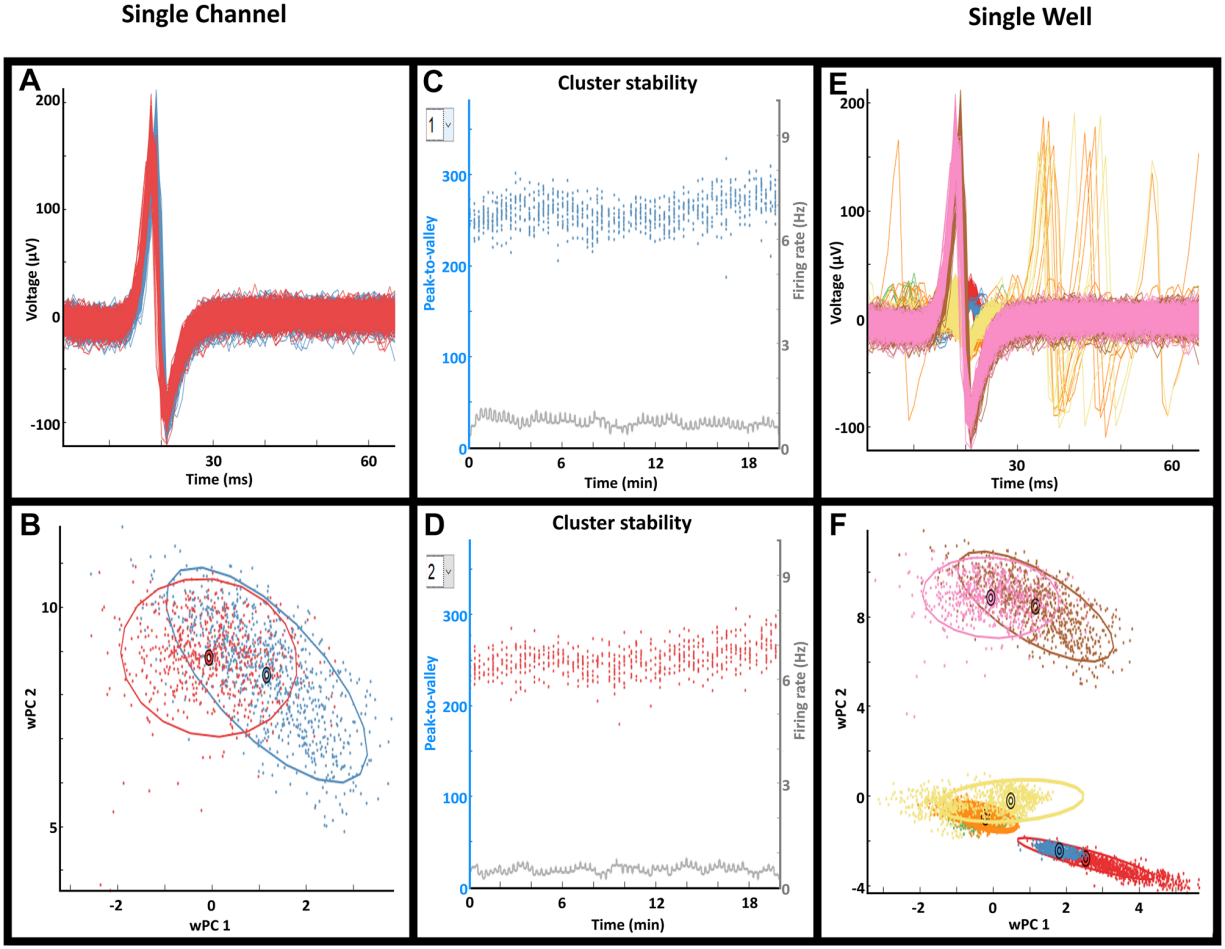
Spike sorting analysis. Examples for the output of the spike sorting analysis. **A**-**D**: the output of channel 6 of well 6 of a multiwell MEA data set containing 12 electrodes/well. **A** and **B** indicate the clusters (separated by color) that were found for this channel whereas **C** and **D** show the cluster stability over time for two different clusters, measured in terms of peak-to-valley and firing rate. **E** and **F** show the 7 clusters found of all 12 electrodes (channels) of well 6 of the multiwell data set

*MEA-ToolBox* also includes tools for spike sorting and saving the detected clusters and the sorted spike waveforms. After loading the raw voltage traces, spike waveforms of a selected channel can be inspected by using the pull-down menu. After spike sorting, the different spikes are organized per channel and displayed in the ‘’sorted waveforms’’ panel for inspection. Spike sorting can be performed on a channel-by-channel basis (Fig. 6A), or for the whole MEA-well in the case of a multiwell data set (Fig. 6B, 12 channels per well). After the spike sorting process is finished the user can visualize the detected clusters via the ‘’clusters button’’. The user can visualize the clusters, either as a scatterplot of two dimensions in the clustering space or as a line plot of all five dimensions in the clustering space. Also provided is a plot of the cluster stability which is defined as the peak-to-valley distance of waveforms over the whole recording. The peak to valley distance of the spike waveform was calculated by taking the difference between the maximum value and the minimum value of the spike waveform. The detected clusters can be saved via the save button which will store the detected clusters in a.mat file which contains the spikes and which cluster they belong to and if they are not sorted than the spikes will denoted as nan. The.mat file also contains the created model used to classify the spikes with the mean (mu), covariance matrix (S) and weight of each gaussian (alpha). Lastly, the file contains the clustering space of each channel. For example, Fig. 6A shows that channel 6 of well 6 has two detected clusters that both show stable peak-to-valley values of the waveforms over the whole recording. Furthermore, there are no major deviations in firing rate indicating that, most likely, the spikes are sorted correctly. Another example can be seen in Fig. 6B, in which a whole MEA-well (well 6) was spike sorted. Within the well, seven clusters were identified, indicating that the spikes could come from seven different neurons.

### Application of *MEA-ToolBox* to a Published MEA Data Set

To demonstrate key features of *MEA-ToolBox* on experimental data, a published MEA data set was processed with default parameter settings. The data set contained recordings from a multiwell plate (with 24 wells, and 12 channels/well) from hiPSC-derived neurons from a patient with Kleefstra syndrome, for which a combination of custom-written scripts and toolboxes was used to demonstrate features of disturbed network excitability (Frega et al., 2019).

**Fig. 7:**
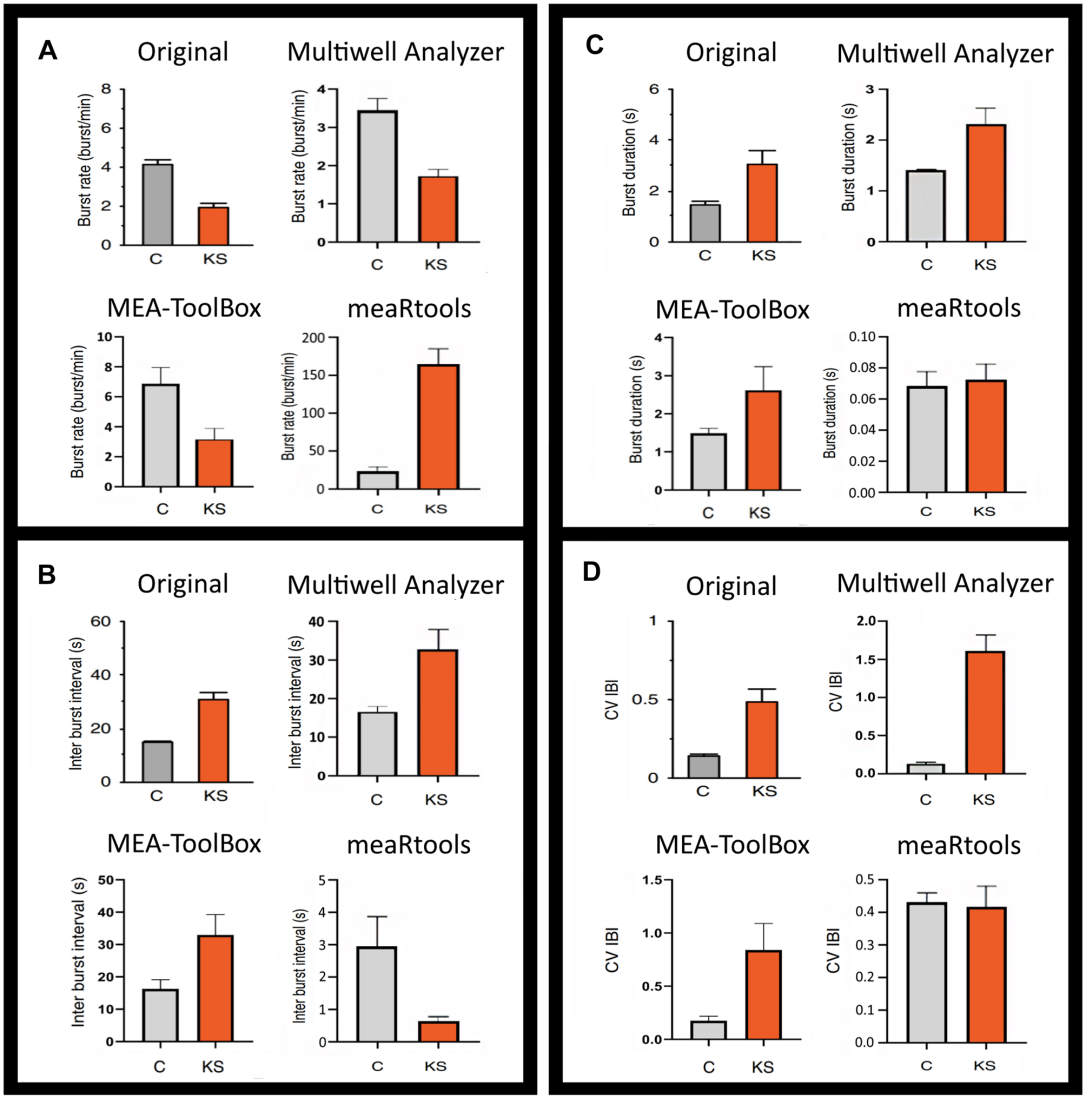
Comparison between toolboxes. Visualization of the outcome of different toolboxes using bar graphs in which network-related neuro endpoints are compared to results of a published dataset for similarity. For the comparison only those neuro endpoints are shown that were reported in the original paper^7^ (Kleefstra cultures show lower network burst rate but longer network burst duration, network IBI, and network CV IBI). **A**: The burst rate per min, **B**: the inter burst interval, **C**: burst duration and lastly **D**: the coefficient of variation of the inter burst interval. The *MEA-ToolBox* and the *multiwell analyzer* were able to output the same results reported in the original paper. *MeaRtools* could replicate the finding that Kleefstra cultures had less network bursts and a higher network burst IBI but in contrast to the published data, network burst duration and network burst CV of IBI did not differ between the two groups (not shown). For the *multiwell analyzer* (as well as the *meaRtools*) the network burst IBI and network burst CV of IBI needed to be calculated manually as they were not included as an output

Figures 7A and B compare the visualization of the data using the original data set (Fig. 7A) with the results from *MEA-ToolBox* (Fig. 7B), including a selection of four network-related neuro endpoints calculated as an average of all active electrodes per condition. The selection of neuro endpoints was based on parameters reported in Frega et al. (2019) In the *MEA-ToolBox* results, control cultures show a higher single-channel fire and burst rate, burst duration and count. In line with the report of Frega et al. (2019); the Kleefstra cultures show lower network burst rates but longer network burst duration, network IBI, and network CV IBI. These results were obtained using the GUI of *MEA-ToolBox*, which did not require the need of any coding expertise and demonstrated the ease of extracting metrics from the data using *MEA-ToolBox*.

### Comparison with *meaRtools *and *Multiwell-Analyzer*

To illustrate how *MEA-ToolBox* functions in comparison to existing toolboxes, the Kleefstra data set was also analyzed with *Multiwell-Analyzer* and *meaRtools*. Both of the toolboxes contain tools for burst analysis, while connectivity analysis and spike sorting are not included. *Multiwell-Analyzer* is a stand-alone free software package designed to analyze multiwell MEA data (Pasquale et al., 2008), but is restricted to the multi-channel system data format. *Multiwell-Analyzer* only provides four network burst endpoints: start time stamp, duration, spike count and spike frequency. To acquire values for network burst IBIs and network burst CV of IBI, manual calculation is required. For these endpoints, *Multiwell-Analyzer* reproduced published results for the Kleefstra data set, i.e. with respect to the number of network bursts (being less frequent), longer burst durations and longer inter-burst intervals (Fig. 7C). *MeaRtools* is a software package created and available only in R and therefore difficult to use for unexperienced users without R coding expertise (Gelfman et al., 2018). We performed the spike detection using the *MEA-ToolBox* before converting the MEA data into the CSV format needed to use *meaRtools*. For comparison, we only used the above-mentioned parameters reported as in Frega et al. (2019) Similarly to the *Multiwell-Analyzer*, we manually calculated the network burst IBI and network burst CV of IBI. The results obtained with *meaRtools* showed that Kleefstra cultures had less network bursts, higher network burst IBI and the network burst duration and network burst CV of IBI did not differ between the two groups (data not shown).

## Discussion

Standardization of MEA analysis is currently challenging as many researchers use different tailor-made analyses. We here provide a streamlined open source analysis toolbox that is freely available and does not require any coding expertise. Furthermore, for users with coding expertise, we have provided a detailed description of the code in the manual starting from page 30, allowing modification of the tools. *MEA-ToolBox* contains all the basic analysis tools needed for MEA analysis, so that spike and burst detection, connectivity, and spike sorting as well as all classical visualization options for MEA data are provided together in one package.

*MEA-ToolBox* enables analysis of raw voltage traces as well as time stamp data, including spike detection, burst detection, network burst detection and additional tools. The software is compatible with different MEA formats (e.g. 60- and 120-channel single-well as well as multiwell MEAs), and provides 20 neuro endpoints to ease interpretation of the MEA data. For data visualization, multiple tools are added such as raster plots, heatmaps and full traces. We also added a spike sorting component for characterizing individual spike data from the multiunit data sets (Souza et al., 2019). *MEA-ToolBox* can analyze multiple files in series which avoids file-by-file analysis and allows keeping the same analysis parameters thus streamlining the analysis process.

In addition, *MEA-ToolBox* has connectivity and spike sorting options. We believe that adding these tools will be beneficial for users as it can help with understanding the MEA data better. *MEA-ToolBox* can draw all putative functional connections one-by-one using a visualization tool that allows the user to obtain information about the dynamics of in vitro neuronal networks. We added the directionality to the connections as well for signal propagation studies, which can be used for analysing cortical spreading depolarization events with directionality or investigate the spatio temporal events of the firing patterns on the MEA. Assessment of these functional connections has been proposed to be a potential tool to indicate if an in vitro culture is healthy or not (Monssink et al., 2021). For spike sorting, in principle, each detected cluster can represent a different putative cell, but it is important to note that the amplitude of action potentials can strongly decrease during repetitive burst firing, and spike sorting may erroneously categorize these as different clusters. Caution should, therefore, be taken when interpreting such clusters. Still, spike sorting can be very useful, as shown, for example, by Becchetti et al. (2012) to help discriminate between inhibitory and excitatory neurons. A suggestion for future *MEA-ToolBox* improvements would be to quantify the peak to valley stability, by evaluating the amount of variability over time and setting a threshold (as a percentage of a defined range of variability, parameters set as fixed or depending on the mean or median).

## Limitations of *MEA-ToolBox*

Although our *MEA-ToolBox* software contains a wide variety of tools to analyze MEA data there are some limitations. The entire code is written using MATLAB scripts, which means that the interpretation of the code is slower compared to using for example MEX files. However, since *MEA-ToolBox* was made for offline analysis, fast processing times are not critical. We chose not to make use of MEX files because multiple functions for the visualization tools within the GUI were not compatible with MEX files. As a general concern, the time/speed needed to analyze the data and work with the GUI is dependent on the hardware of the user’s PC. The bigger the data set is, the more ram is needed. For example, with 16 GB ram, a 10 min-long voltage recordings (roughly 4 GB) of a 12-well MEA multiwell (288 individual channels) will take on average 30 min to analyze, which can be broken down into 3 distinct phases: 4 min for spike detection, 7 min for single-burst detection and 18 min for network related metrics. For files containing only time stamps, the analysis will be much faster, only taking a few minutes.

We alleviated some of the limitations by employing several tricks such as splitting the data in multiple pieces before combining them together later in the analysis pipeline when needed. *MeaRtools* does not have this limitation because it does not need to work with voltage traces and only works with spike time stamps data, making the analysis much faster. This makes *meaRtools* very suited for users that are experienced in R and want to quickly explore their data. In the case of *Multiwell-Analyzer*, this package is specialized to analyze multiwell MEA data and does not pose a limit on the amount of data it can process. Exploratory studies usually contain large data sets and if they are multiwell data sets then the *Multiwell-Analzyer* is more suited for these types of exploratory studies.

Another limitation of *MEA-ToolBox* is that the incorporated algorithms are not parameter-free. The benefits are that the user can fine tune parameters but this means that the neuro endpoints are not data-driven but based on the parameters that are set. As a result, features of the data set could be missed or distorted if the user does not know what kind of parameters to use. This is why default values are provided for each parameter based on literature. However, if the user wants to perform a more exploratory analysis, parameter-free methods such as the Otsu method to detect network bursts—used in *meaRtools*—would be preferred. This might also explain the difference found in the analysis of the Kleefstra data set using *meaRtools* compared to *MEA-ToolBox*, as *meaRtools* used the parameter-free method for network burst detection. Although *meaRtools* could replicate the findings of the original publication that Kleefstra cultures showed fewer network bursts and a higher network burst IBI, in contrast to the publication, network burst duration and network burst CV of IBI did not differ between the two groups, whereas *MEA-ToolBox* replicated also the latter. Neither the methods used in the *MEA-ToolBox* nor the methods used in *meaRtools* are wrong because there is no golden standard and we believe that each approach serves different purposes. There is a great example of this in the paper of Cotterill et al. (2016) that used 8 different methods to detect single-channel bursts from literature to compare their performance. The outcomes show that even if one tries to use the same parameters and compare them, different methods do not necessarily give the same results. The authors discussed that they cannot conclude on a perfect method for burst detection and recommend to choose a burst detector based on the degrees of freedom the user wants to control. Nevertheless, two methods were found to perform best in the analysis, i.e. the max interval method and the log ISI method, which is why we have chosen to include these in our *MEA-ToolBox*. Both the *meaRtools* and *Multiwell-Analyzer* are more specialized toolboxes tailored for users with either R coding expertise or exploratory studies and we also recommend these toolboxes for those users. Lastly, Dastgheyb et al. (2020) have compiled a table (*cf* table ESM1) containing most of the toolboxes found in the literature and have scored them per feature, which gives an excellent overview of the previously published toolboxes limitations.

## Conclusion

The focus for the *MEA-ToolBox* was to represent a general toolbox for MEA data analysis that is user friendly while containing a multitude of tools for users which have no experience in coding. *MEA-ToolBox* will automatically detect spikes and collect information about spike activity, bursting behavior and network related metrics and is provided with an intuitive GUI interface. Lastly, we will provide continuous support for the development of *MEA-ToolBox* as an open source program on Github (e.g. make toolbox compatible with more file formats from other MEA manufacturers), and encourage more advanced users to provide add-on packages for increased modularity and new features or improve the existing scripts, so that *MEA-ToolBox* can become and remain a universal and standardized MEA analytical tool.

## Information Sharing Statement

*MEA-ToolBox* is freely available at the Github repository (https://github.com/mhyhu/MEA-ToolBox/tree/master). This also contains a link to a google drive which contains some test data HDF5 files used in this manuscript https://drive.google.com/drive/folders/1IrS9-TnZHw8d7IW7TlJVqvXeNlGGVtY3?usp=sharing.

## Supplementary Information

Below is the link to the electronic supplementary material.

Supplementary file1 (TIFF 9834 KB)

Supplementary file2 (TIFF 1999 KB)

Supplementary file3 (TIFF 11568 KB)

Supplementary file4 (TIFF 2097 KB)

Supplementary file5 (TIFF 18229 KB)

Supplementary file6 (DOCX 11 KB)

Supplementary file7 (DOCX 8049 KB)

Supplementary file8 (TIFF 2040 KB)
