# Supplementary figures and images for: MEA-ToolBox: an Open Source Toolbox for Standardized Analysis of Multi-Electrode Array Data
Source: Neuroinformatics. 2022 Jun 9;20(4):1077–92. doi: 10.1007/s12021-022-09591-6 (PMC9588481; doi:10.1007/s12021-022-09591-6)

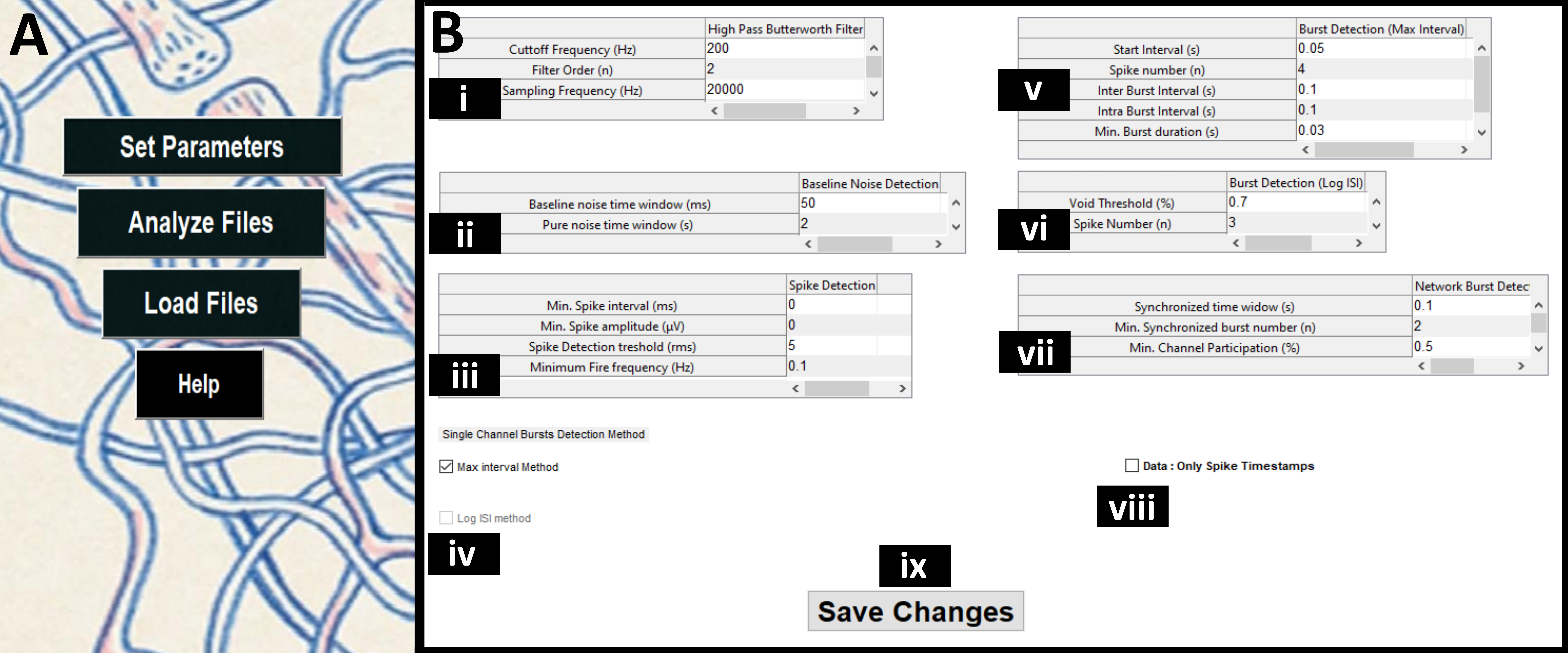

Supplement: Supplementary file 1 — Supplementary file1 (TIFF 9834 KB) [file 12021_2022_9591_MOESM1_ESM.tiff]

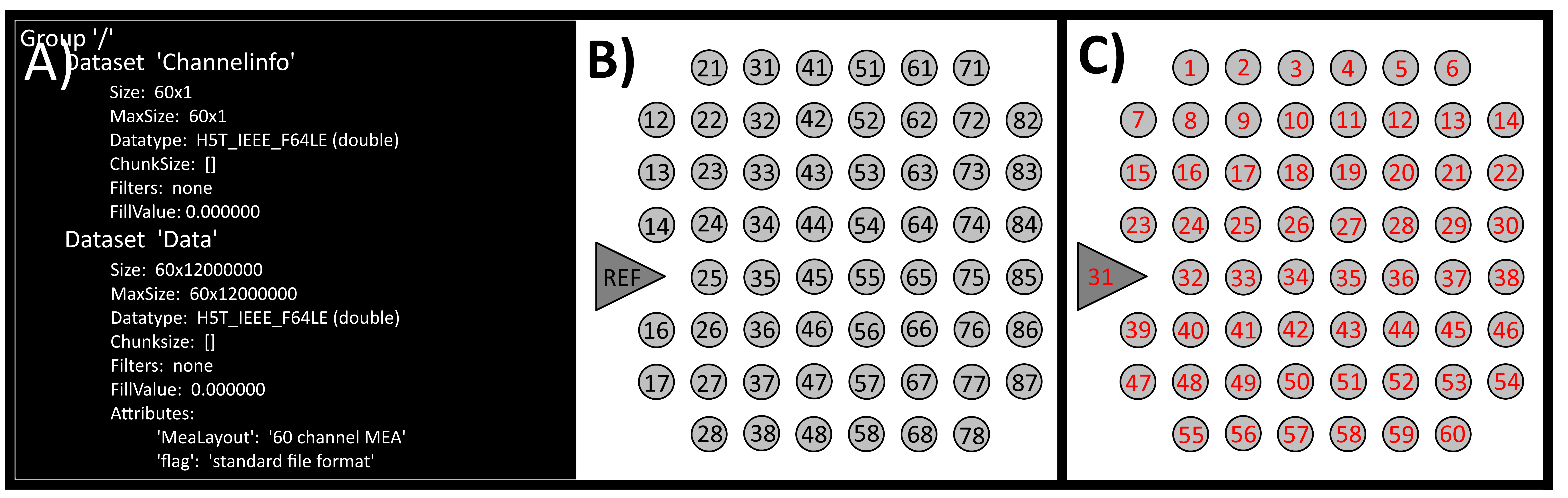

Supplement: Supplementary file 2 — Supplementary file2 (TIFF 1999 KB) [file 12021_2022_9591_MOESM2_ESM.tiff]

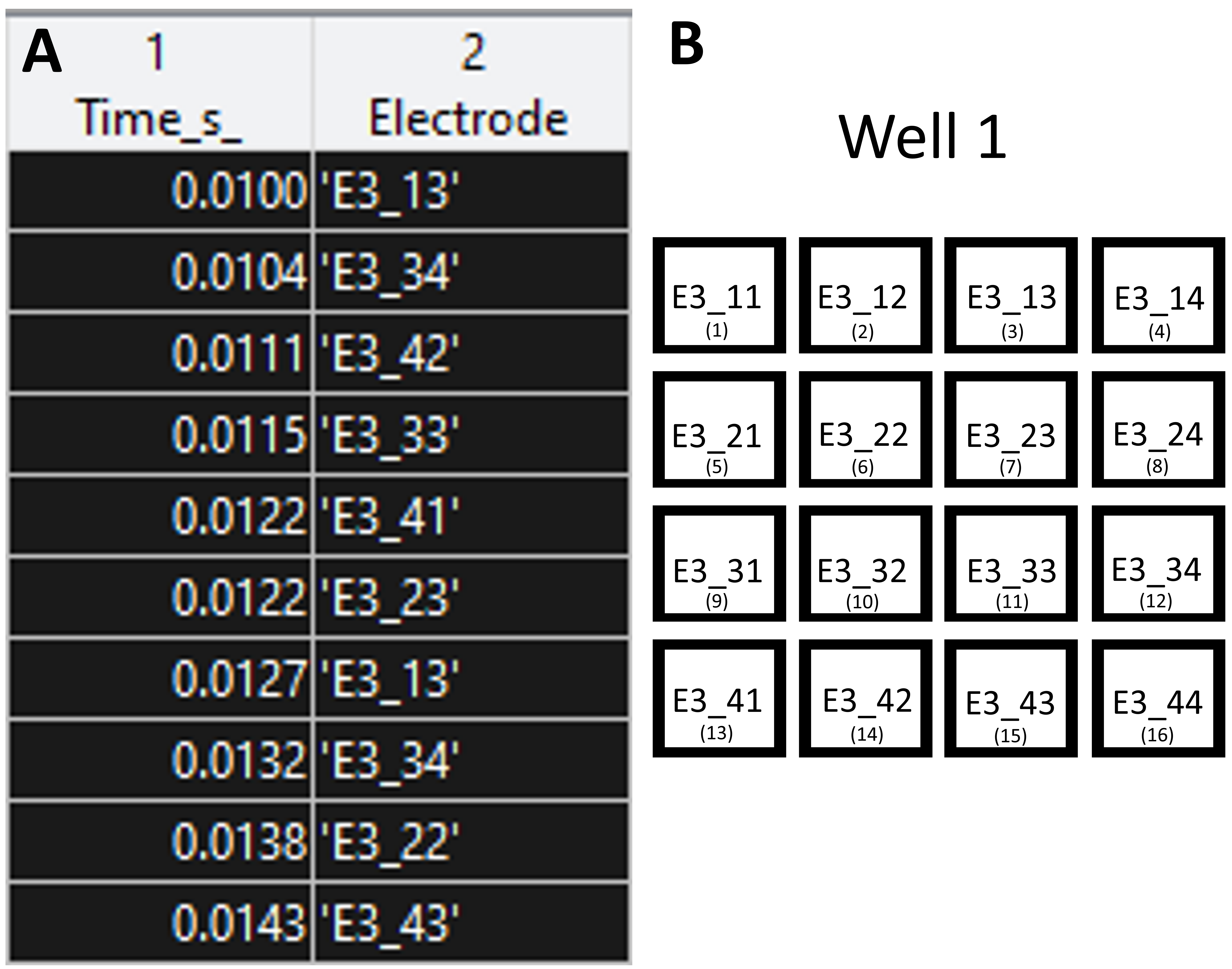

Supplement: Supplementary file 3 — Supplementary file3 (TIFF 11568 KB) [file 12021_2022_9591_MOESM3_ESM.tiff]

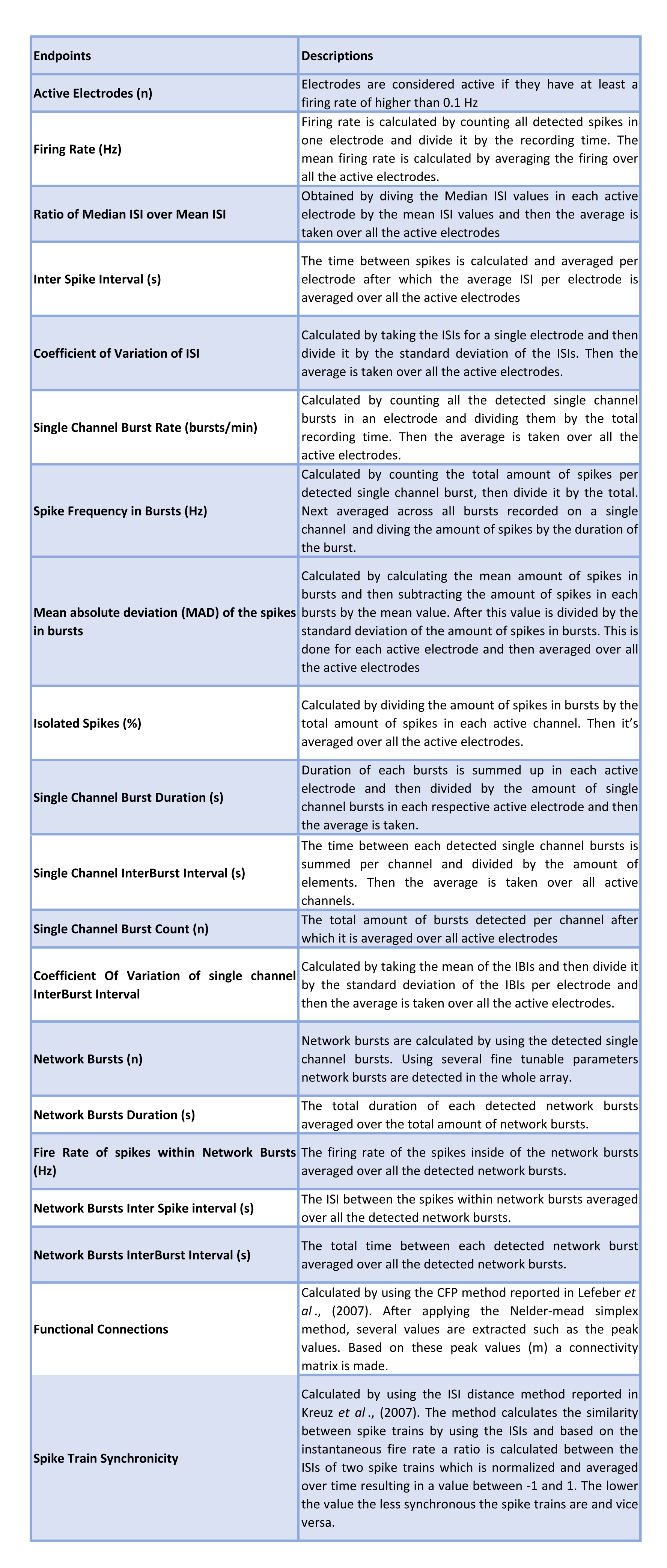

Supplement: Supplementary file 4 — Supplementary file4 (TIFF 2097 KB) [file 12021_2022_9591_MOESM4_ESM.tiff]
