# Supplementary material for: MEA-ToolBox: an Open Source Toolbox for Standardized Analysis of Multi-Electrode Array Data
Source: Neuroinformatics. 2022 Jun 9;20(4):1077–92. doi: 10.1007/s12021-022-09591-6 (PMC9588481; doi:10.1007/s12021-022-09591-6)
Supplement: Supplementary file 6 — Supplementary file6 (DOCX 11 KB) [file 12021_2022_9591_MOESM6_ESM.docx]

<https://github.com/mhyhu/Toolbox>
