# Supplementary material for: MEA-ToolBox: an Open Source Toolbox for Standardized Analysis of Multi-Electrode Array Data
Source: Neuroinformatics. 2022 Jun 9;20(4):1077–92. doi: 10.1007/s12021-022-09591-6 (PMC9588481; doi:10.1007/s12021-022-09591-6)
Supplement: Supplementary file 7 — Supplementary file7 (DOCX 8049 KB) [file 12021_2022_9591_MOESM7_ESM.docx]

**MEA-ToolBox:**

***User Manual***

**Table of Contents**

| **1. How to get started** |  |  |  | **4** |
| --- | --- | --- | --- | --- |
| 1.1 Installation |  |  |  | 4 |
| 1.2 Getting started |  |  |  | 4 |
| 1.3 How to use the toolbox |  |  |  | 6 |
| 1.3.1 Set parameters |  |  |  | 6 |
| 1.3.1.1 Filtering parameters |  |  |  | 7 |
| 1.3.1.2 Baseline noise detection |  |  |  | 7 |
| 1.3.1.3 Spike detection |  |  |  | 8 |
| 1.3.1.4 Single-channel burst detection (Max Interval) | |  |  | 8 |
| 1.3.1.5 Single-channel burst detection (LogISI) |  |  |  | 8 |
| 1.3.1.6 Network burst detection |  |  |  | 8 |
| 1.3.1.7 Single-channel burst detection method |  |  |  | 8 |
| 1.3.1.8 Only spike timestamps data format |  |  |  | 9 |
| 1.3.1.9 Save changes |  |  |  | 9 |
| 1.4 Analyse files |  |  |  | 10 |
| 1.5 Load files |  |  |  | 10 |
|  |  |  |  |  |
| **2. Home panel** |  |  |  | **11** |
| 2.1 Overview of all channels |  |  |  | 11 |
| 2.1.1 Single-channel information |  |  |  | 11 |
| 2.1.1.1 Filtered voltage trace |  |  |  | 11 |
| 2.1.1.2 Single-channel spike raster plot |  |  |  | 11 |
| 2.1.1.3 Single-channel firing rate histogram |  |  |  | 12 |
| 2.1.1.4 ISI histogram |  |  |  | 12 |
| 2.1.1.5 Single-channel bursts |  |  |  | 12 |
| 2.2 Voltage trace view or most active channels |  |  |  | 12 |
| 2.3 Colour bar |  |  |  | 12 |
| 2.4 General information |  |  |  | 13 |
| 2.5 Whole array spike raster plot and heatmap |  |  |  | 13 |
| 2.6 Analyse files |  |  |  | 13 |
| 2.7 Load files |  |  |  | 13 |
| 2.8 Full voltage trace view |  |  |  | 13 |
| 2.9 Remove channels |  |  |  | 13 |
| 2.10 Top channels |  |  |  | 13 |
| 2.11 MEA movie activity |  |  |  | 14 |
| 2.12 Neuro endpoints |  |  |  | 14 |
| 2.13 Heatmap |  |  |  | 14 |
| 2.14 Raster plot |  |  |  | 15 |
|  |  |  |  |  |
| **3. Bursts** |  |  |  | **16** |
| 3.1 Select channel |  |  |  | 16 |
| 3.2 Switch view |  |  |  | 16 |
| 3.3 Rerun burst detection |  |  |  | 16 |
| 3.3.1 Finish burst detection |  |  |  | 17 |
| 3.3.2 Burst detection methods |  |  |  | 17 |
| 3.3.3 Rerun burst detection buttons |  |  |  | 17 |
| 3.4 Rerun network burst detection |  |  |  | 17 |
| 3.4.1 Finish network burst detection |  |  |  | 18 |
| 3.4.2 Network burst detection methods |  |  |  | 18 |
| 3.4.3 Rerun network burst detection buttons |  |  |  | 18 |
| 3.5 Zoom in bursts |  |  |  | 18 |
| 3.6 Burst statistics |  |  |  | 19 |
| 3.7 Button panel |  |  |  | 19 |
|  |  |  |  |  |
| **4. Connectivity** |  |  |  | **20** |
| 4.1 Load |  |  |  | 20 |
| 4.2 Option buttons |  |  |  | 20 |
| 4.3 Visualize connections |  |  |  | 21 |
| 4.4 Export figure |  |  |  | 21 |
| 4.5 Select channel |  |  |  | 21 |
|  |  |  |  |  |
| **5. Spike sorting** |  |  |  | **21** |
| 5.1 Load spike waveforms |  |  |  | 21 |
| 5.2 Save spike sorted waveforms |  |  |  | 21 |
| 5.3 Run sorting |  |  |  | 21 |
| 5.4 Run all channels |  |  |  | 21 |
| 5.5 Display wells |  |  |  | 22 |
| 5.6 Clusters |  |  |  | 22 |
| 5.7 Reset |  |  |  | 22 |
| 5.8 Edit unsorted |  |  |  | 22 |
| 5.9 Plot all waveforms |  |  |  | 22 |
| 5.10 Discard waveforms |  |  |  | 22 |
| 5.11 Unsorted spike waveforms |  |  |  | 22 |
| 5.12 Sorted spike waveforms |  |  |  | 22 |
| 5.13 Waveforms |  |  |  | 22 |
| 5.14 Resort |  |  |  | 22 |
| 5.15 Delete |  |  |  | 23 |
| 5.16 Cluster stability |  |  |  | 23 |
|  |  |  |  |  |
| **6. Neuro Endpoints and their definitions** |  |  |  | **24** |
| **7. Output of *MEA-ToolBox* and their definitions** |  |  |  | **26** |
| **8. Design of *MEA-ToolBox* for modifying the code** |  |  |  | **30** |
| 8.1 Main_Menu_Toolbox.m |  |  |  | 30 |
| 8.2 MEAToolboxV3.m |  |  |  | 31 |
| 8.3 Multiwell_Mainbody.m |  |  |  | 44 |
| 8.4 Justspiketimes.m |  |  |  | 46 |
| 8.5 CSVfiles.m |  |  |  | 47 |
| **9. References** |  |  |  | **49** |
|  |  |  |  |  |
|  |  |  |  |  |

**1. How to get started**
 **1.1 Installation**

The standalone application of *MEA-ToolBox* can be accessed freely at:

<https://github.com/mhyhu/MEA-ToolBox>

and can be installed on a Windows 10 PC. The toolbox needs MATLAB 2018b runtime and it will download it. *MEA-ToolBox* start up icon can be double clicked to launch *MEA-ToolBox*. The full source code can be found on GitHub so that users experienced in MATLAB can work directly from MATLAB if desired. This manual was written to further explain how to use *MEA-ToolBox.* Users are referred to download the publication for further insights.

**1.2 Getting started**

When starting *MEA-ToolBox,* a terminal window will open. This terminal window can be ignored but is useful to keep open as it lists any errors it encounters. Therefore, it will be very useful for troubleshooting. *MEA-ToolBox* is currently only compatible with HDF5 files and CSV files. We chose this file format because it is supported by MATLAB, Python and R. The user must convert their data files first into a HDF5 or CSV file format before the toolbox can be used. We describe how the data should be organised in either the HDF5 or CSV file format below. If the user uses hardware from multi-channel systems (MCS) the easiest way would be to use their data converter: <https://www.multichannelsystems.com/software/multi-channel-datamanager>.

If the user does not use MCS, raw voltage traces can be converted into HDF5 files according to a layout seen in figure 1A. The HDF5 file should contain one group with two datasets within this group. One dataset named ‘’Data’’ contains all the raw recording data in an M-by-N matrix. M in this matrix represents the channels and the N the samples in each channel. An example can be seen in figure 1A where ‘Data’ contains a 10-min recording of a 60-channel MEA recorded at 20 kHz. Important is to also include within the ‘Data’ the attribute ‘MEA layout’ to indicate what kind of MEA the data is from (i.e. 60- or 120-channels MEA, or a multiwell MEA). Lastly, it is important to include a ‘flag’ attribute and name it ‘*standard file format’*. The other dataset named ‘*Channelinfo*’ should contain information about the number of channels and the spatial location of the channels. For example, in figure 1B is a layout of a 60 MEA from MCS and in figure 1C is how the toolbox interprets the channel location information. The channel 1 in the matrix of the standard file format should contain channel data from channel ‘’21’’ and channel 2 should contain information of channel ‘’31’’ etc. If the user has a more specialized layout of the electrodes, a new datafile can be easily created to fit the user layout on request.

**
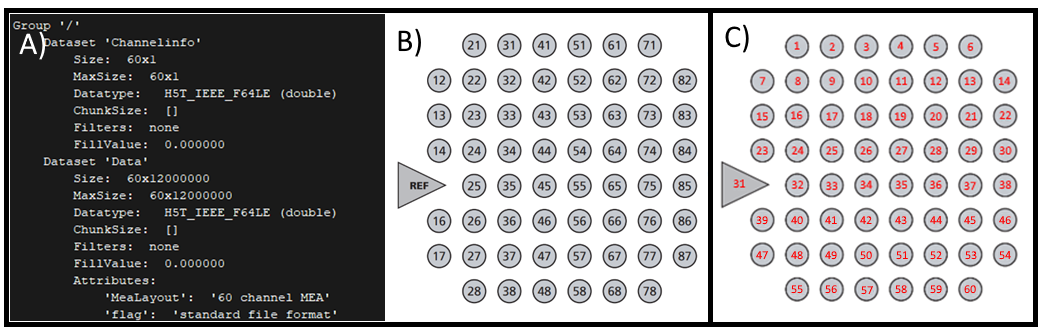
Figure 1***. File layout of the HDF5 file format for voltage trace data***.**

*MEA-ToolBox* is also compatible with CSV files. Currently, the user can analyse a CSV file like the HDF5 files, whereby the user only has to press the ‘analyse’ button. *MEA-ToolBox* will determine by itself if the data concern an HDF5 file or a CSV file. The analysis will be the same for a CSV file as an HDF5 file, however due to the lack of voltage trace data the analysis will likely be faster. When the analysis is finished the output is a .mat file which is described in more detail on page 26 (section 7). Using the GUI for datasets without voltage trace is possible, however the options that require voltage trace data will not be available.

If the user wants to convert data into a CSV file, this is possible by organising the file as follows. There needs to be one column containing the spike timings which can be named anything but needs to contain ‘time’ in it. Another column needs to contain the information from which channel the spike originates from, and the column name needs to contain ‘Electrode’ in it. These two columns need to be matched with each other. For example, in figure 2A, the 2^nd^ spike in the whole recording occurs at 0.0104 seconds than 0.0104 will be listed in the 2nd row in the time column and in the same row for the Electrode column there needs to be the channel name from where the spikes originate from. There is no other information needed to make it compatible with *MEA-ToolBox*. However, there is one assumption made with respect to the layout of the electrodes: the layout is assumed to be as in the layout of the 24 multiwell from axion biosystems in which the electrodes are organised in a 4x4 pattern. This means that each well contains 16 electrodes, and the numbering goes from left to right and from top to bottom. An example of how the first well is organised can be seen figure 2B.


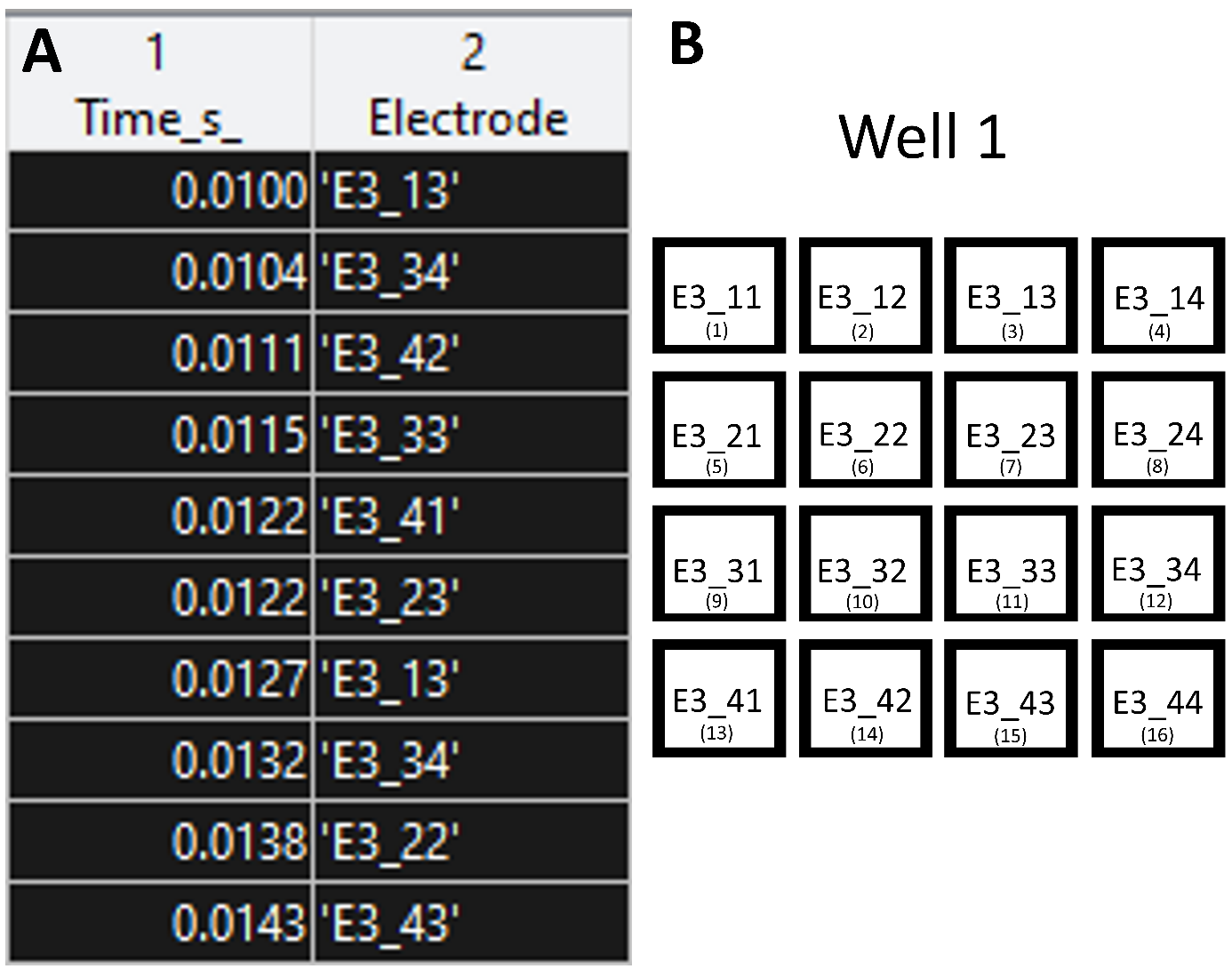


**Figure 2.** *CSV file organisation*

**1.3 How to use *MEA-ToolBox***

After the installation is finished the toolbox can be started by double clicking on the icon installed on your desktop. The user will be presented with the start screen shown in figure 3. Besides the ‘Help’ button, only a ‘Set Parameters’ button is shown at the start. The correct parameters must be set before subsequent buttons become available.


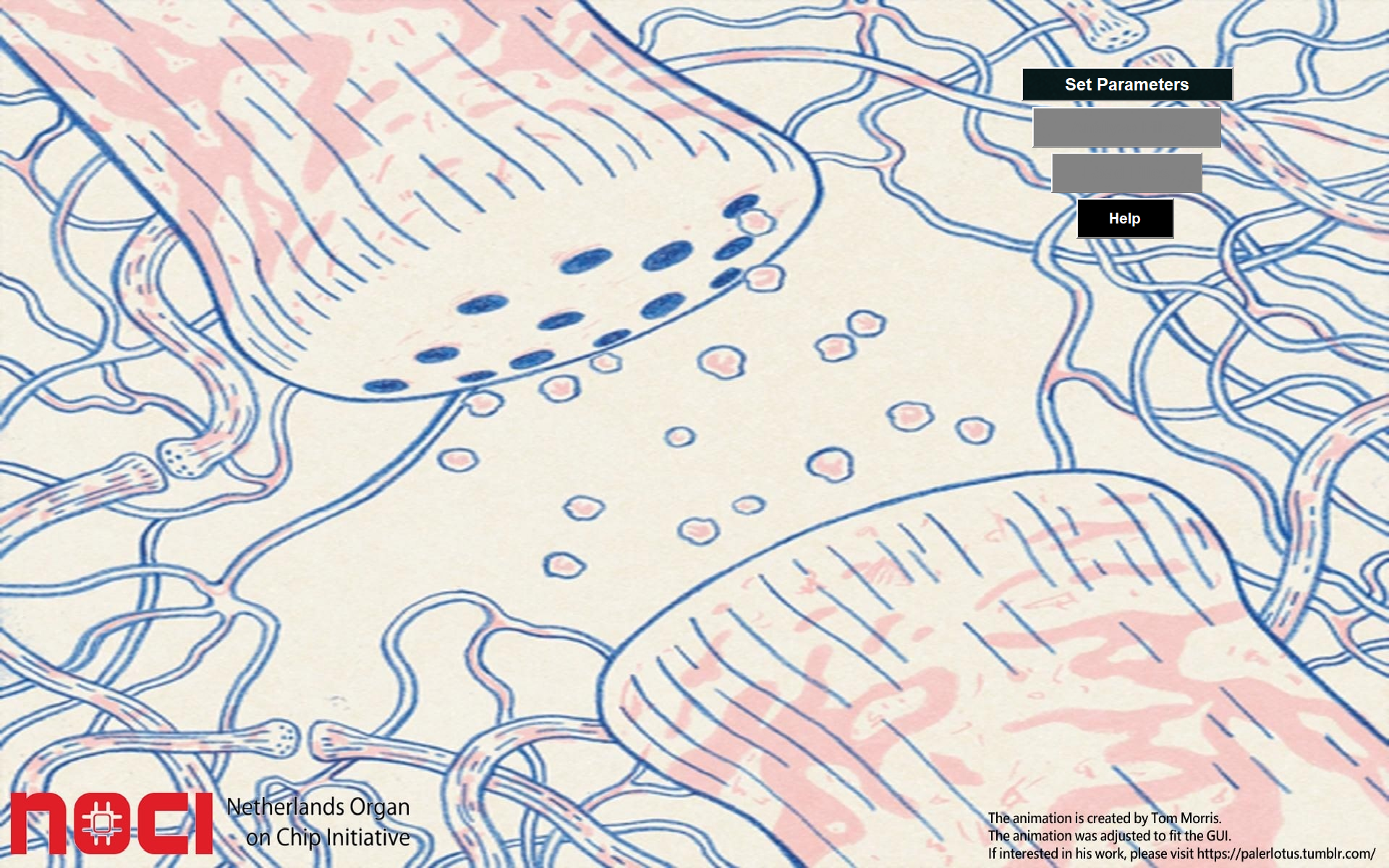


**Figure 3.** *Start screen. Set parameters, analyse files (greyed out) and save changes (greyed out).*

**1.3.1 Set parameters**

Before starting the analysis procedure, it is important to verify that the settings such as the sampling frequency used are set correctly. A new window will appear for the user that highlights the options for adjustments (figure 4). Each value can be altered by clicking on the number and typing in the desired values.


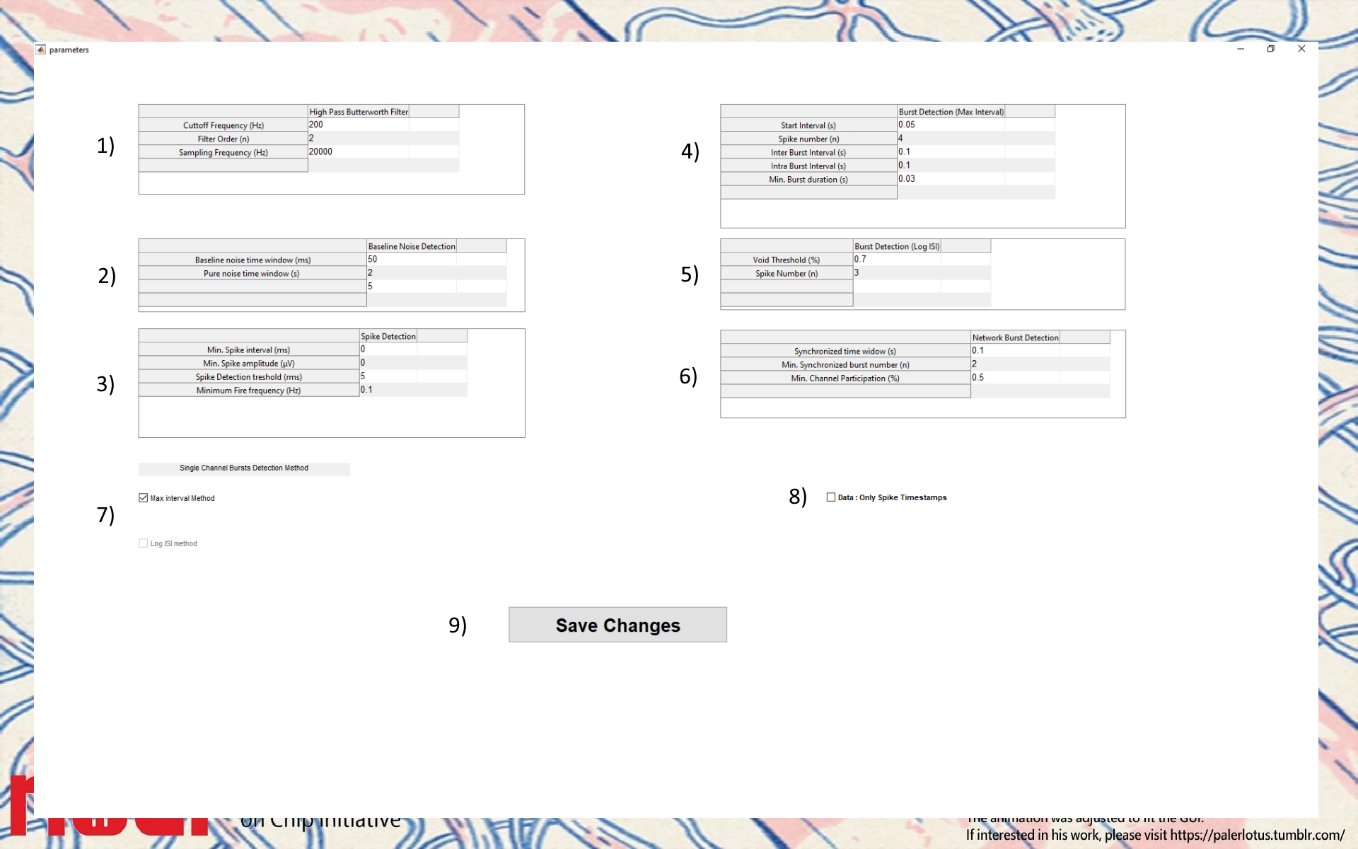


**Figure 4.** *Setting the correct parameters for data analysis. 1) Filtering parameters, 2) Baseline Noise Detection, 3) Spike Detection, 4) Single*-*Channel Burst Detection (Max Interval), 5) Single*-*Channel Burst Detection (Log ISI), 6) Network Burst Detection, 7) Single*-*Channel Bursts Detection Method, 8) using data containing spike timestamps only and 9) save changes.*

**1.3.1.1 Filtering parameters**

The analysis procedure uses a high pass Butterworth filter to remove low frequency components and make the spike detection procedure easier.

*-Cuttoff Frequency (Hz):* Frequency at which the filter operates. For example, a cut-off of 200 Hz indicates that all frequencies above 200 Hz are allowed through the filter while any frequencies below this cut-off frequency are attenuated.

*-Order of fit (n):* The order of a fit determines how well the filter performs. The higher the order the better the filtering process but the downside is an increase in complexity leading to longer processing time.

*-Sampling Frequency (Hz):* The frequency at which data is sampled within one second; this value must match the recording sampling frequency used. For example, if 20000 data points are sampled within one second, the sampling frequency that should be set is 20000 Hz.

**1.3.1.2 Baseline noise detection**

Before spike detection can take place, the analysis procedure must determine the baseline noise so it can set a threshold without manual intervention.

*-Baseline noise time window (ms):* This parameter represents the size of each bin when the data is split in smaller windows to determine which section of the recording is pure noise.

*-Pure noise time window (s):* This parameter indicates the minimum duration of the time window that is used to establish the baseline noise.

**1.3.1.3 Spike detection**

*-Min. Spike interval (ms):* The minimum time between spikes. If there are any spikes detected that have a time interval between spikes of less than this parameter will be removed.

*-Min. Spike amplitude (µV):* This parameter represents the minimum voltage that a spike should be. Any detected spikes that have a peak value of less than this parameter will be removed.

*-Spike Detection threshold (rms):* This parameter represents how strict the threshold is for spike detection. The higher this number is, the stricter the threshold.

*-Minimum Fire frequency (Hz):* The minimum firing rate a channel should have to be included in the analysis.

**1.3.1.4 Single**-**channel burst detection (Max Interval)**

*-Start Interval (s):* The maximum time interval between the first two spikes of a burst.

*-Spike number (n):* The minimum number of spikes that a burst should contain.

*-Inter Burst Interval (s):* The maximum time interval between detected bursts. If the time interval is smaller than this parameter, the bursts will be combined.

*-Intra Burst Interval (s):* After finding the start of the burst using the Start Interval parameter, the algorithm will determine if the following spikes belong to the burst based on this parameter. The interval between the spikes after the 2 initial spikes are found, should be less than this parameter.

*-Minimum Burst Duration (s):* The minimum duration a burst should be.

**1.3.1.5 Single**-**channel burst detection (Log ISI)**

-Void Threshold (%): The threshold for determining if the peaks detected in the log ISI histogram are separated well enough. If the calculated value is higher than this parameter, the peaks are separated well enough.

-Spike Number (n): The minimum number of spikes that a burst should contain.

**1.3.1.6 Network burst detection**

*-Synchronized time window (s):* the maximum time window that the analysis procedure will look in the data to determine if bursts are firing synchronously. If bursts from different channels are firing within this time window, these bursts are considered as network bursts.

*-Min. Synchronized burst number (n):* the minimum number of synchronized bursts needed to be considered a network burst.

*-Min. Channel Participation (%):* the minimum number of channels that have synchronized bursts that should be participating in the network burst.

**1.3.1.7 Single**-**channel bursts detection method**

The user has the option to change the method that is used to detect single-channel bursts. Note that this will influence the network burst detection as these single-channel bursts are the basis to detect the network bursts.

**1.3.1.8 Only spike time stamps data format**

The *MEA-ToolBox* is also compatible with data containing only spike stamps. However, similar to the voltage data the spike timestamps must be presented as an HDF5 file before it its compatible with the toolbox. The user must convert their data into an HDF5 file format with a similar layout as portrayed in figure 5. There are two groups one which contains all the spike stamps data, and the other group contains information about the recording such as what kind of MEA was used. For the group that contains all the spike stamp data, important is to split the spike waveform and the time of the spike into two datasets. For example, channel 1 has 438 detected spikes therefore, there is one dataset called Data_1 which contains the spike waveforms of all the 438 spikes (the toolbox will read the spike waveforms horizontally). In addition, there should be a dataset called Data_ts_1 that contains all the spike times corresponding to the spikes of Data_1. This should be done for the channels that contain spike data. Important is that spike times should be converted to seconds and then multiplied by 1000000. Please use the same naming convention as seen in figure 4. The other group that contains information about the data should be the same as the one from the HDF5 for voltage trace data (See section 1.2 getting started) but with one addition which is the attribute called ‘Duration’ should be added. The duration attribute should be in seconds and multiplied by 1000000. An example of a compatible HDF5 file is shown in figure 4 where there are only two channels that contain spike data from a 1 hour recording for illustrative purposes.

**1.3.1.9 Save changes**

The user must press the save changes button otherwise the changes will not be saved. The default values for each parameter were taken from the literature. While *MEA-ToolBox* is analysing the raw data files (which can be multiple files in series from the same folder), a progress bar will be shown to indicate the progress of each data file. After the analysis procedure is finished a graphical user interface (GUI) will appear of the last file that was analysed (figure 6).





**Figure 5.** *File layout of the HDF5 file format using spike stamps data.*

**1.4 Analyse files**

The user will be prompted to select a folder that contains HDF5 files to start the analysis process.
*MEA-ToolBox* will analyse all the HDF5 files within the selected folder using the same set parameters.
Important to remember is that *MEA-ToolBox* will currently **only** accept HDF5 files (See 1.2 Getting started for more information). The result is analysed .mat files (MATLAB) that are saved inside a new folder titled ‘Analysed files’.

**1.5 Load files**

The user can then open analysed .mat files with *MEA-ToolBox* to view the results of the analysis.


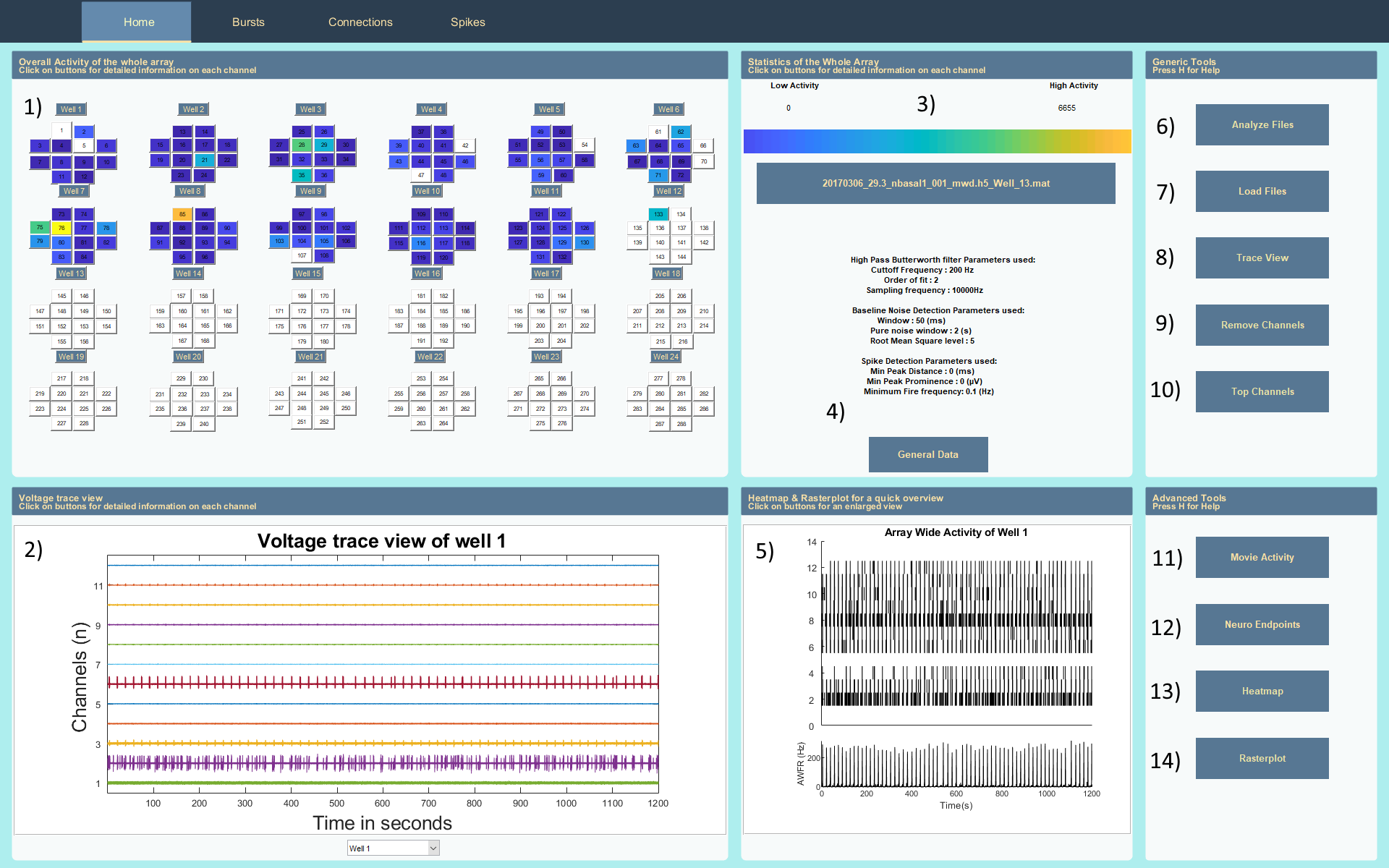
**2. Home panel**

**Figure 6.** *MEA-ToolBox home screen. 1) Overview of all channels and single*-*channel information, 2) Voltage trace view and most active channels, 3) Colour bar, 4) General information, 5) Whole array spike raster plot and heatmap, 6 to 10) Generic tools and 11 to 14) Advanced tools.*

**2.1 Overview of all channels**

Overview of all the channels in the well depending on the layout of the MEA it can change from a single-well to a multi well layout. An example is given of a multiwell data set (figure 6(1)). This overview allows for a quick overview of the active and non-active channels represented by their colour. Each channel can be pressed for additional single channel information, see below.

**2.1.1 Single channel information**

When a single-channel is pressed, a new screen will appear that will provide additional information about the channel (figure 7). *This function will not work if the dataset does not contain any voltage trace data.*

**2.1.1.1 Filtered voltage trace**

On the top, the filtered voltage trace is displayed of the selected channel and which part of the voltage trace is used to set the threshold for spike detection together with the thresholds used for spike detection.

**2.1.1.2 Single**-**channel spike raster plot**

Spike raster plot of a single-channel in which each black line represents a detected spike.

**2.1.1.3 Single**-**channel firing rate histogram**

Histogram of firing rate over the whole duration of the recording.

**2.1.1.4 ISI histogram**

Histogram of the distribution of the inter spike interval (ISI).

**2.1.1.5 Single**-**channel bursts**

Spikes are visualized together with single-channel bursts (either max interval or log ISI method).


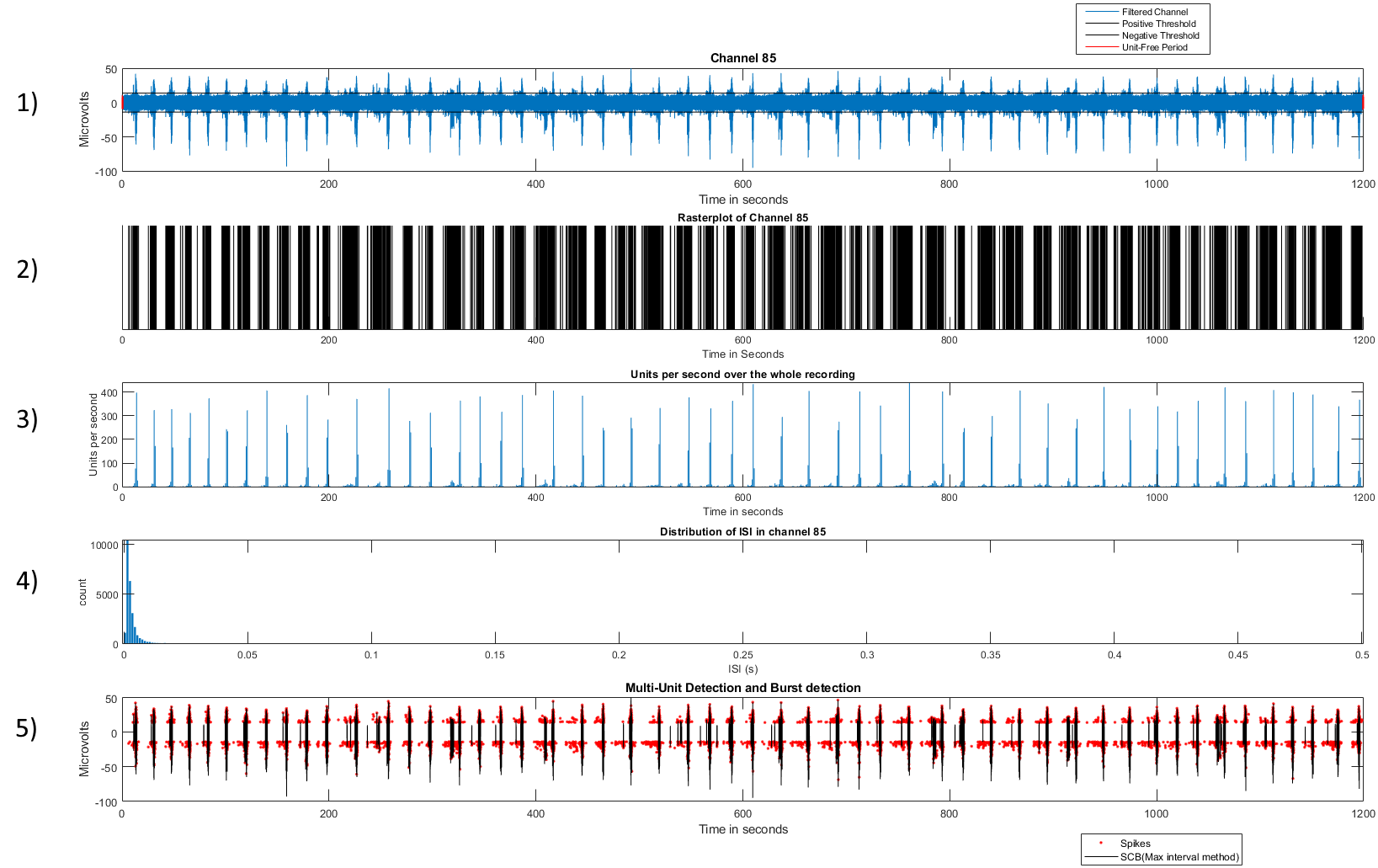


**Figure 7.** *Single*-*channel information. 1) Filtered voltage trace, 2) Single*-*channel spike raster plot, 3) single*-*channel firing rate histogram, 4) ISI histogram and 5) Single*-*channel bursts.*

**2.2 Voltage trace view or most active channels**

Depending on if it is a single-well or multiwell MEA dataset the panel will be different. In the case of a multiwell dataset (shown in figure 6) the panel will display the voltage traces of all channels in well 1. The voltage view can be changed to display other wells by using the drop box below the panel. This allows for a quick overview of the voltage traces of one well. If the dataset is a single-well MEA dataset, the panel will display the 5 most active channels of the dataset. If needed the user can press the button and a bigger display will be given of the most active channels that allows for a more in-depth look.

**2.3 Colour bar**

The colour bar that highlights the overall activity of the whole well or multiple wells with the number at low activity representing the lowest number of spikes detected in the whole well in a single-channel whereas the number at high activity represents the highest number of spikes detected in a single-channel. Note that the colour bar’s location is different depending on if the dataset is from a multiwell or single-well MEA.

**2.4 General information**

The name of the data file can be found here as well as the parameters used to analyse the file. Below, the information about which parameters are used there is a general data button. The general data button can be pressed for a table that contains spike train metrics about each individual channel (for example, ISIs, firing rates and single-channel bursts).

**2.5 Whole array spike raster plot and heatmap**

Depending on the dataset, this panel is also different. If the dataset is a single-well MEA data, then there will be two separate panels which contain a heatmap on the left side and a raster plot of the whole array on the right side. The heatmap on the left side is based on the number of spikes in which high amounts of spikes are represented as yellow and low amounts of spikes are in blue. For a more detailed view of the heatmap, or if the heatmap needs to be saved, the heatmap button can be pressed. On the right side is the whole array spike raster plot in which each black line represents a spike. Below the raster plot is a plot containing the array wide firing rate of the whole recording. In the case of a multiwell dataset, only the whole array spike raster plot is present as can be seen in figure 6.

**2.6 Analyse files**

The user will be prompted to select a folder that contains HDF5 files (or CSV files) to start another analysis process. *MEA-ToolBox* will analyse all the HDF5 files (or CSV files) within the selected folder using the same set parameters. Important to remember is that *MEA-ToolBox* will currently **only** accept HDF5 files and CSV files (See 1.2 Getting started for more information). The result is analysed .mat files that are put inside a new folder that is created within the selected folder titled ‘Analysed files’.

**2.7 Load files**

The user can open analysed .mat files inside the analysed folder created after the analysis procedure is finished.

**2.8 Full voltage trace view**

The user can view the voltage traces of all the channels in one well. This means 120 voltage of a 120 channel MEA however in the case of the multiwell this would mean for example 12 channels if there are 12 electrodes per well. If the button is pressed, the user is requested to put in the desired time window to be shown. In the case of a multiwell dataset, the user is also asked to choose a specific well. Once chosen the individual traces will be shown, the user can move through the recording in time by using the left and right arrow keys on the keyboard. The y axis that is shown by default is based on the maximum detected voltage value in the data, however the user can change it by using the up and down keys on the keyboard (zoom in and out). *Note that* *this function will not work if the dataset does not contain voltage trace data.*

**2.9 Remove channels**

The user can delete channels from further analyses by pressing this button. The user will be prompted to type in the selected channel for deletion. Important to realise is that this action is **irreversible,** so if the user wants to get the data back, the user will have to re-load the file.

**2.10 Top channels**

The user can plot the topmost active channel in one go with this button for visual inspection to quickly determine if the spikes were correctly detected.

**2.11 MEA movie activity**

The user can create a video of the spiking activity of the whole recording. High activity patterns are indicated in yellow whereas blue represents low activity or none. The video is accompanied with the 5 most active channels on the right side. The video has a dimension of 640x 480 with 30 frames per second. *Note that* *this function will not work if the dataset does not contain any voltage trace data.*

**2.12 Neuro endpoints**

The Neuro endpoints button allows the user to extract 20 different endpoints that can be used to determine the status of the neuronal cultures (see also section 6). This can only be performed on analysed data files with the .mat extension. The user will be prompted with a question. If all the analysed files of which the 20 endpoints are to be extracted are in one folder already, the user can press continue select the desired folder. If the analysed files are not in one folder the user can select terminate and the user will have to create a new folder that will hold all the analysed files. When all the analysed files are in one folder, a new popup will appear in which the user has to select folder containing all the analysed files to calculate the neuro endpoints for. After collecting all the data there will be an extra prompt if the data is from a multiwell to ask the user if *MEA-ToolBox* can group certain wells together. The user will be asked how many groups there are in the data. Important is that each group must have equal amounts of wells. For example, you have 4 groups of 6 in a 24 multiwell data set. The user can assign which wells belonging to each group. After which the user will be asked to name the newly created excel that will contain all the neuro endpoints (figure 8A). A second popup will appear to name a second excel file. This 2^nd^ excel file is all the neuro endpoints for all files but without the grouping (figure 8B). If the user does not choose to do the grouping, then there will be no extra prompt and a pop up will appear to name an excel file that contains all the Neuro endpoints (figure 8B).


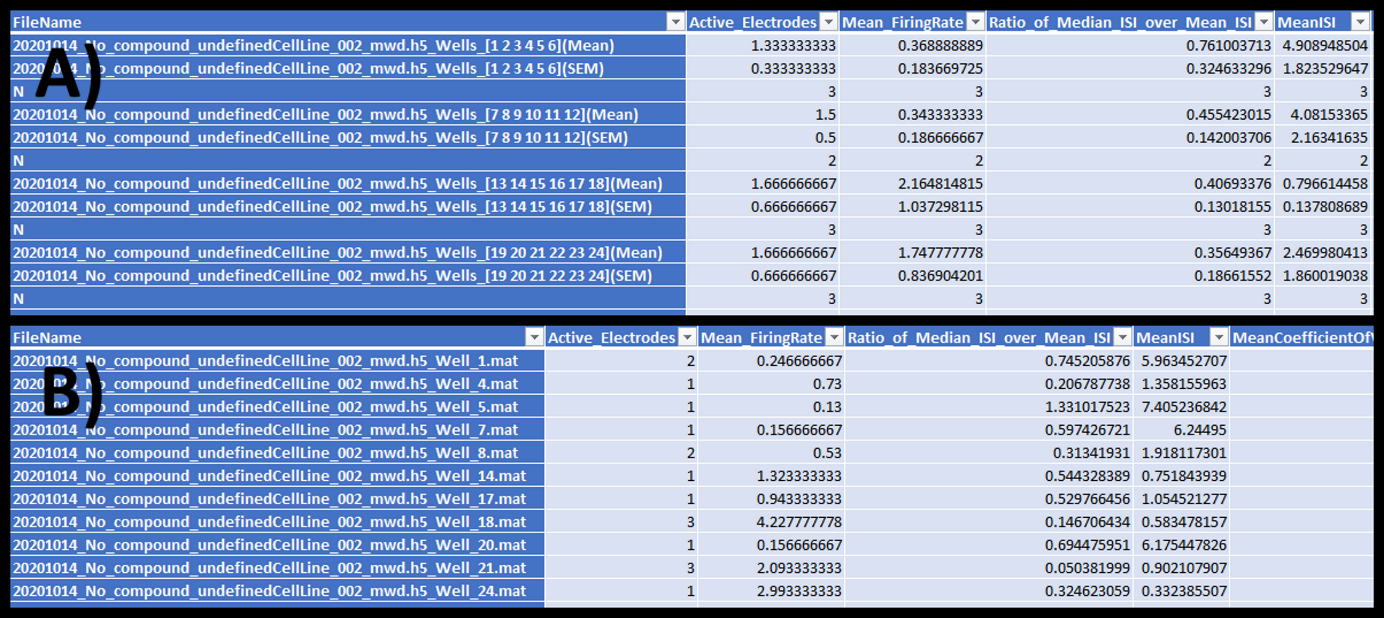


**Figure 8.** *Neuro endpoint measurements. Example of A) grouped endpoints and B) ungrouped endpoints.*

**2.13 Heatmap**

In case the user is interested to have a different view of the overall activity in the well or multiple wells the user can view the overall activity as a heatmap.

**2.14 Raster plot**

The user can press this button to get a more detailed view of the raster plot and array-wide firing rate. Once pressed, a new window will appear, and 2 new options together with a more detailed view of the spike raster plot can be accessed. The single-channel bursts can be displayed in red overlaid with the spike raster plot or the network bursts in blue. The other option will only appear if the dataset is from a multiwell and will be in the lower left corner with a pull-down menu that allows for the selection of each individual well together with their spike density function (figure 9A). It is also possible to zoom in as can be seen in figure 9B where the first 200 seconds are shown of the data where the network bursts are displayed in blue.

**
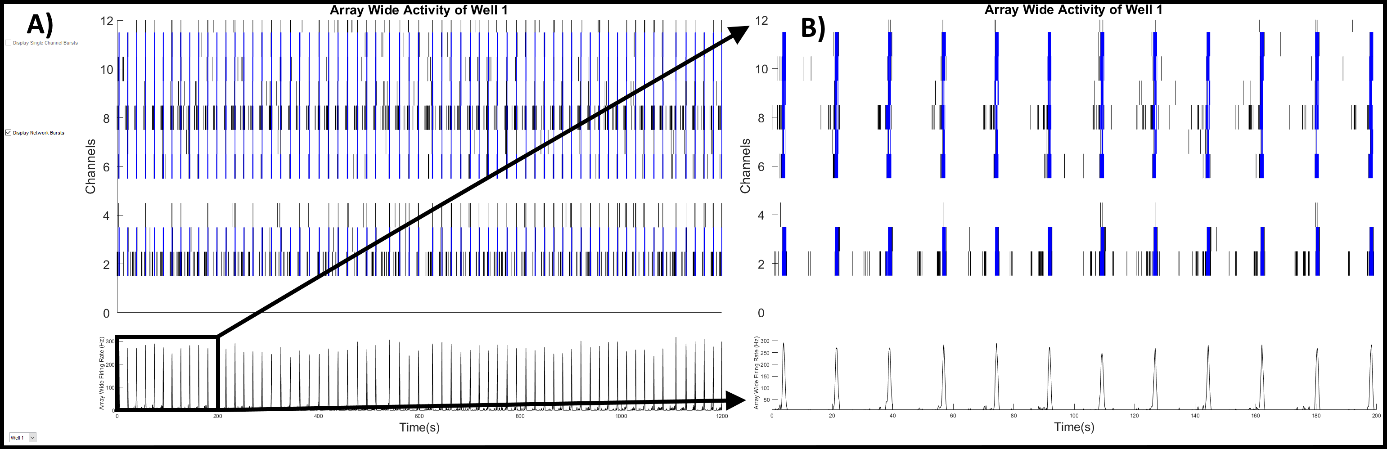
Figure 9.** *Raster plot showing A) array wide overlay of network bursts and B) raster plot zoomed in.*

**3. Bursts panel**

**3.1 Select channel**

Selection of a specific channel that allows for a more detailed investigation in burst detection and network burst detection. Before any other options becomes available, the user must select a channel for investigation (figure 10). Once a channel is selected, several buttons will become available in the button panel. *Note that* *this function will not work if the dataset does not contain any voltage trace data.*


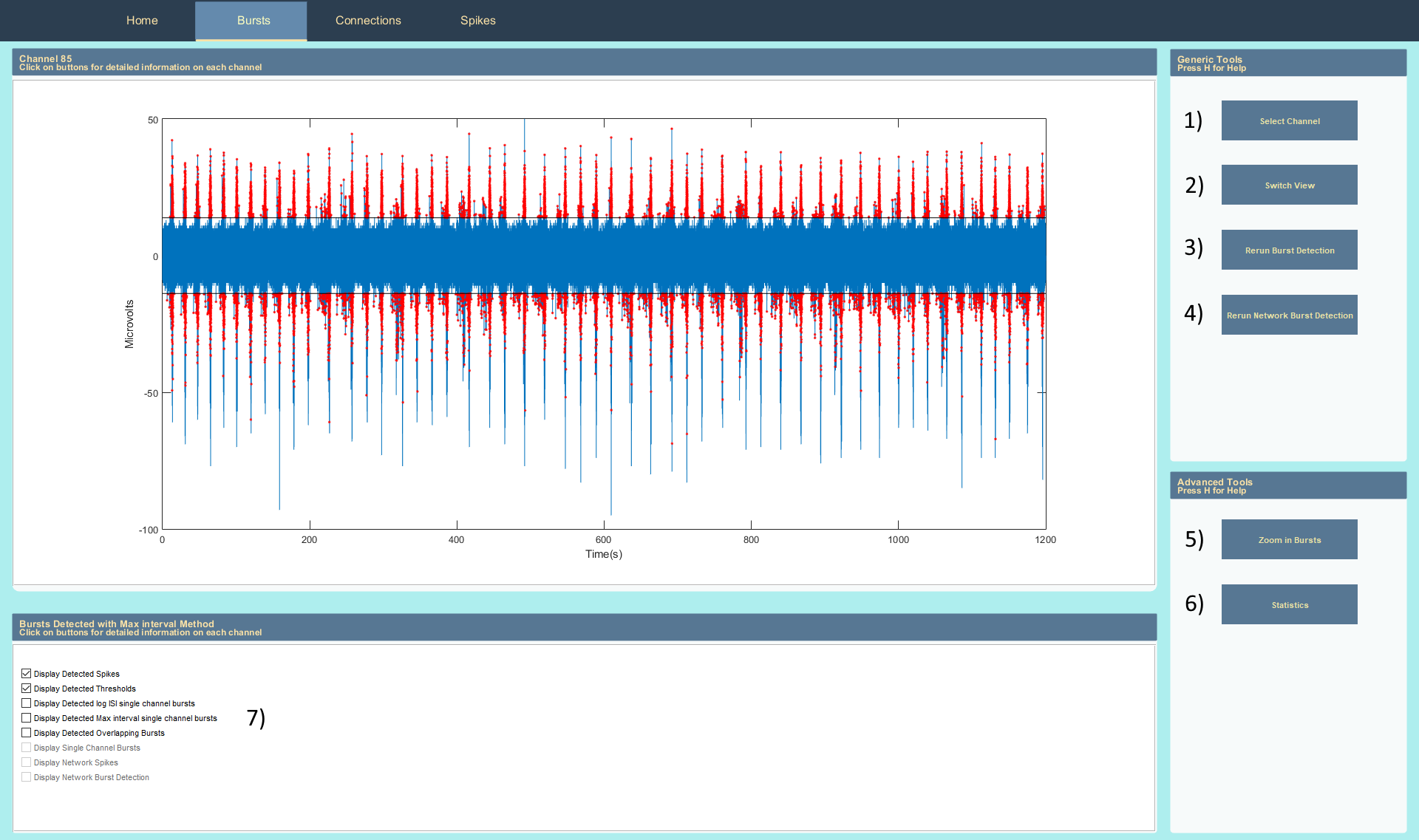


**Figure 10.** *Bursts Screen showing detected spikes and bursts (red) with 1) Select channel, 2) Switch view, 3) Rerun burst detection, 4) Rerun network burst detection, 5) Zoom in burst, 6) Statistics and 7) Button panel.*

**3.2 Switch view**

Switches the panel that displays the voltage trace with the whole array spike raster plot together with the spike density plot. Once this button is selected, two different options will become available in the button panel and the other options will be greyed out. The other buttons will become available again when you press the same button again.

**3.3 Rerun burst detection**

The button panel will change and will reveal the two available burst detection methods as described in the paper and some additional parameters. After a burst detection method is selected, the user can change the parameters that are used to detect the bursts. The result is a comparison between the detected bursts using the previous parameters versus the newly chosen parameters (figure 11). *MEA-ToolBox* will automatically save the bursts detected using the new parameters.

**
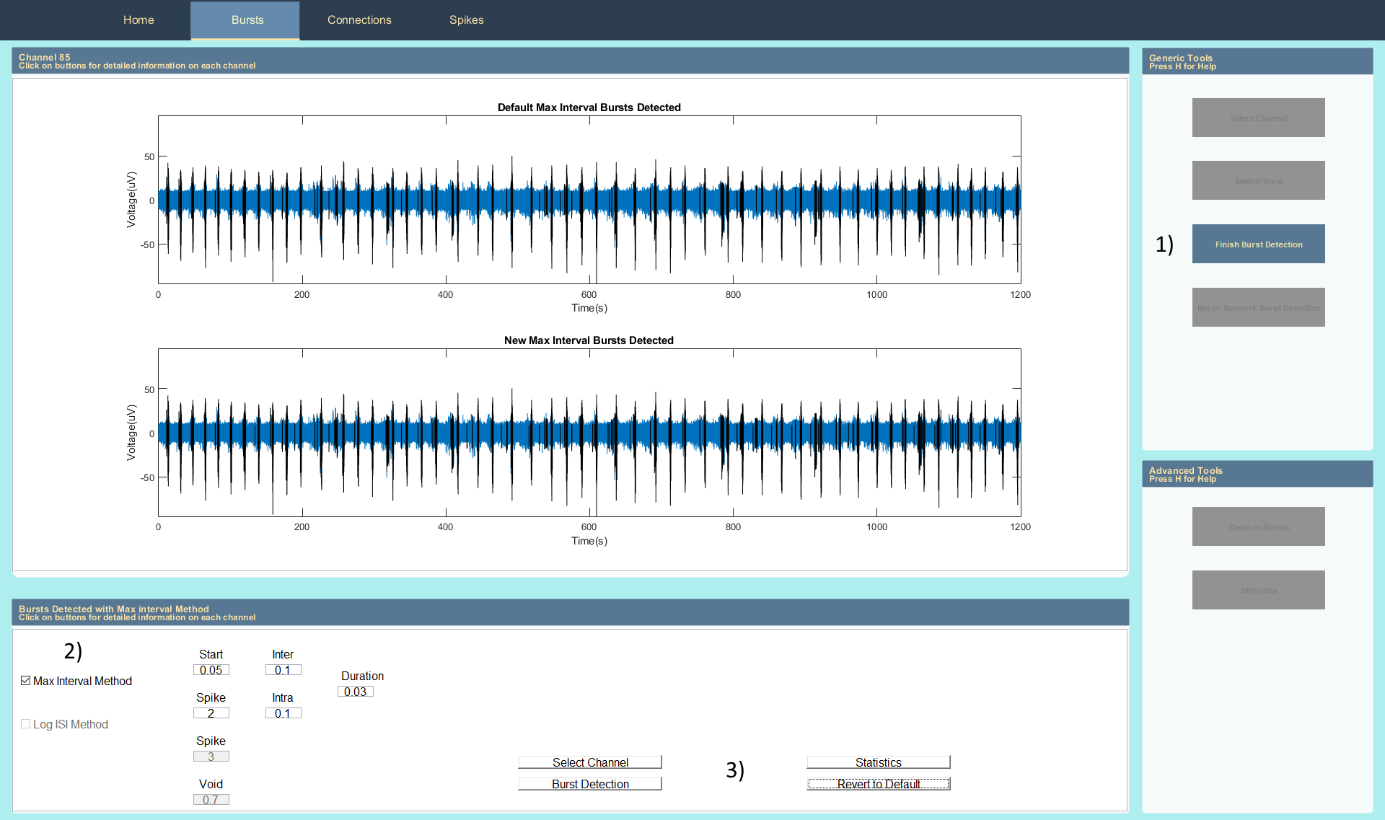
Figure 11.** *Rerun burst detection. 1) Finish burst detection, 2) Burst detection methods and 3) Rerun burst detection buttons.*

**3.3.1 Finish burst detection**

Once the rerun burst detection button is pressed, the name of this button will change and new buttons will appear, and the previous buttons will be greyed out. To get access back to the old buttons the user must press the finish burst detection button.

**3.3.2 Burst detection methods**

The user can choose one of the two detection methods to rerun the burst detection for the selected channel. After a method is selected, the individual boxes for that detection method will become accessible and the user can alter the default parameters. The adjustable parameters are also described in the publication.

**3.3.3 Rerun burst detection buttons**

These 4 buttons are only available when the rerun burst detection is pressed. To rerun the burst detection with the newly changed parameters the user must press the burst detection button. The result is a comparison between the previous selected (default) parameters on the top of the figure versus the result of the newly chosen parameters on the bottom. For the precise numbers, the statistics button can be pressed. The revert to default button can be used to go back to the default settings in case this is needed. An example is given in figure 11 where we changed the minimum number of spikes that is a burst from 4 (top) to 2 (bottom) spikes.

**3.4 Rerun network burst detection**

Similarly, to the rerunning burst detection, this button will allow the user to fine tune the network burst detection without having to rerun the full analysis. After the user has changed the parameters used to detect the network bursts the toolbox will plot both the old detected network bursts (top) and the new detected network bursts (bottom) in blue which will allow for an easier comparison (figure 12).

**
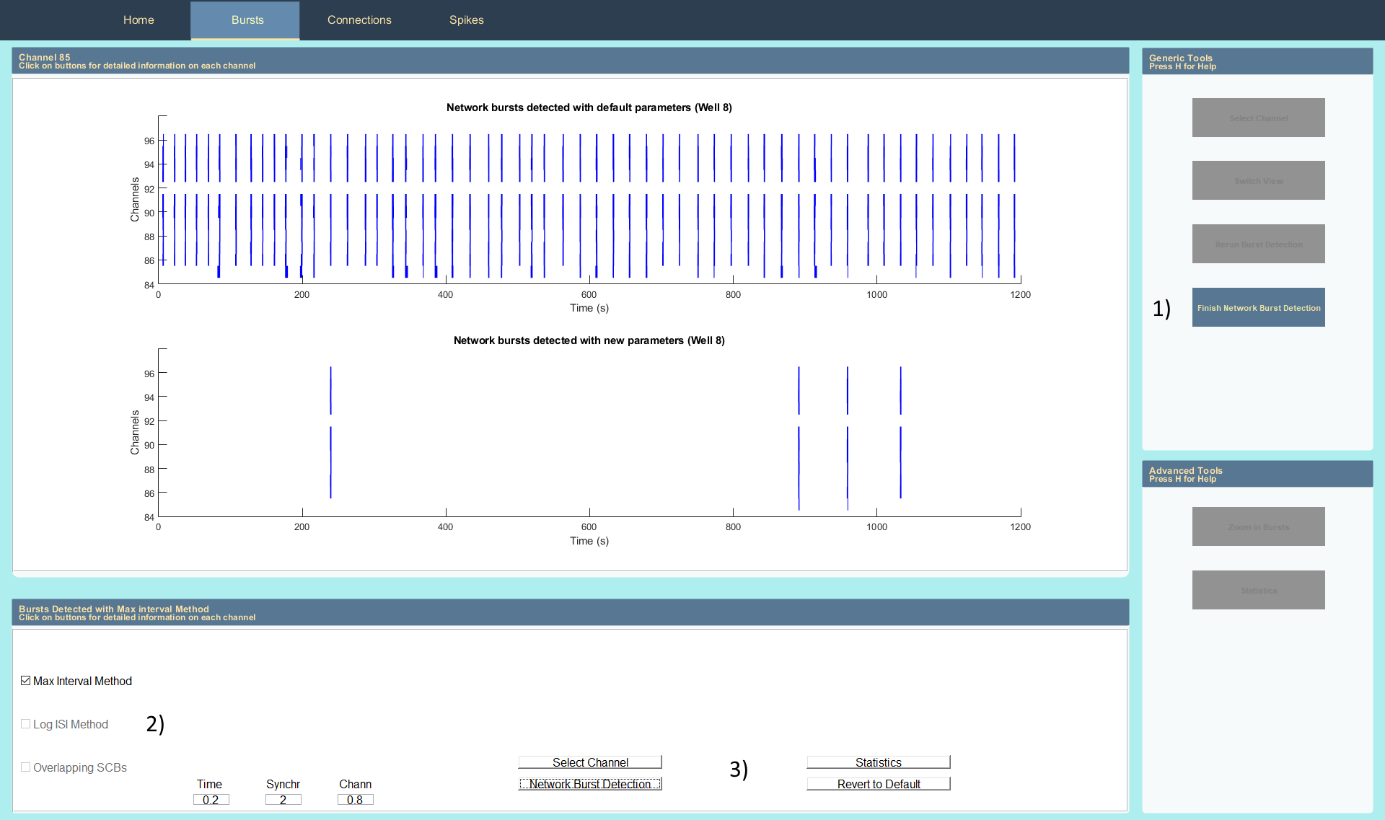
Figure 12.** *Rerun network burst detection. 1) Finish network burst detection, 2) Network burst detection methods and 3) Rerun network burst detection buttons.*

**3.4.1 Finish network burst detection**

When the rerun network burst detection button has been pressed several new options to fine tune the detection will appear. When the user is finished with fine tuning the parameters to detect network bursts, the user must press the finish network burst detection in order to gain back the old buttons.

**3.4.2 Network burst detection methods**

There are several methods that the user can use to detect network bursts and the user must select one before the adjustable parameters become available. The adjustable parameters are also described in the publication.

**3.4.3 Rerun network burst detection buttons**

The 4 buttons found here are the exact same as the 4 buttons found during rerunning burst detection (section 3.3.3.). To rerun the network burst detection with the newly changed parameters, the user must press the network burst detection button. The result is a comparison between the previous selected (default) parameters on the top of the figure versus the result of the newly chosen parameters on the bottom. For the exact numbers, the statistics button can be pressed. The revert to default button can be used to go back to the default settings in case this is needed. An example is given in figure 12 where we changed the minimum percentage of channels that must participate in the network burst to be considered a network burst from 25 to 80%.

**3.5 Zoom in bursts**

When selected, prompt will be given, and a number will be asked. This number is a burst number. For example, when the number 1 is selected the figure will zoom in on the first detected burst. This option is only available when the voltage trace of the selected channel is displayed.

**3.6 Burst statistics**

A table will be presented that contains all the burst metrics for the selected channel of both the max interval method and log ISI method.

**3.7 Button panel**

After a channel is selected, this button panel becomes available. The first four options are only available when the voltage trace is displayed whereas the last 3 options are only available when the switch view button is selected. These buttons allow for the visualization and inspection of the used thresholds for spike detection and burst detection.

**4. Connectivity panel**

**4.1 Load**

The data must be loaded in before the options become available (figure 13).

**
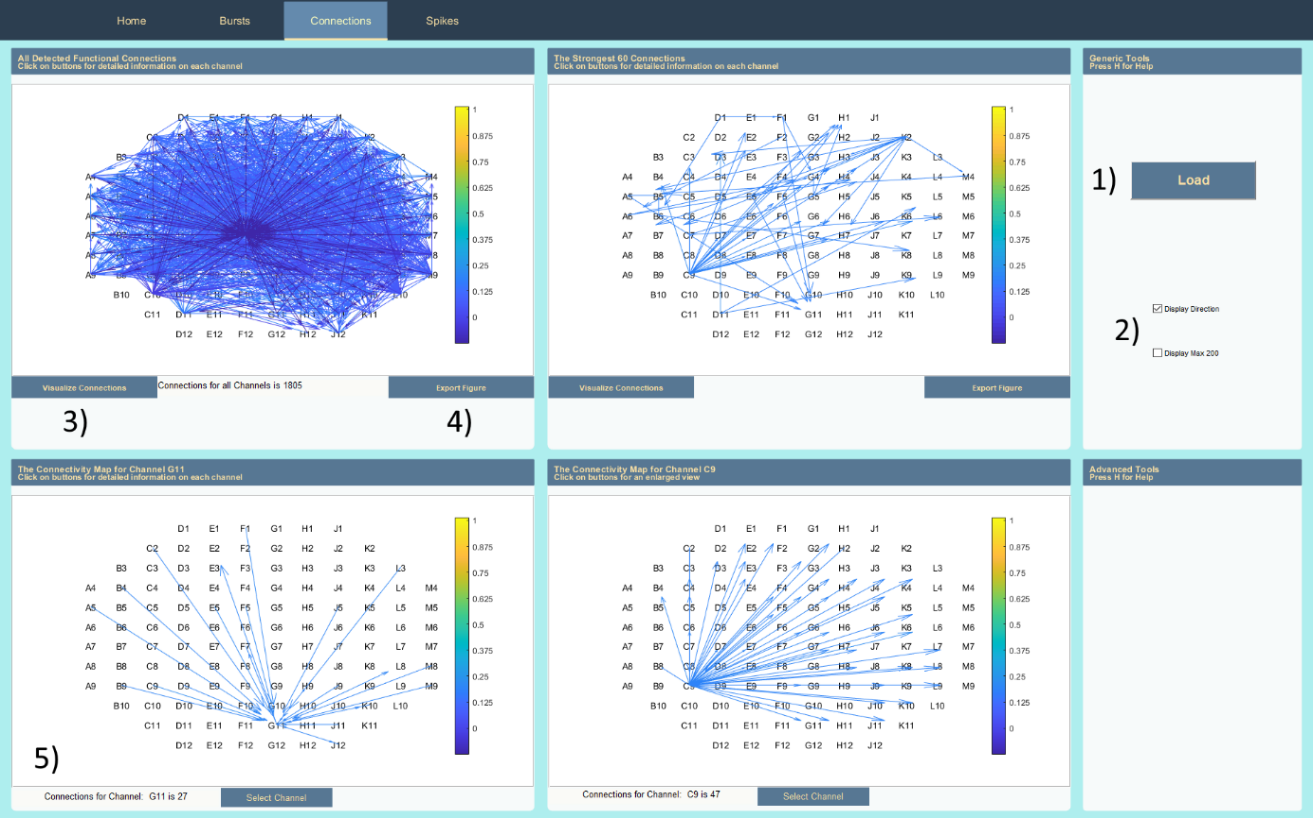
**

**Figure 13.** *Connectivity Screen. 1) Load, 2) Option buttons, 3) Visualize connections, 4) Export figure and 5) Select channel.*

**4.2 Option buttons**

The first option will add arrowheads to the drawn lines, which can be useful for clarity to determine the direction of the connection. The second option limits the number of connections drawn in the top left panel. Normally, all the connections will be drawn. However, if this option is selected, only the top 200 connections will be drawn (with the highest probability).

**4.3 Visualize connections**

Will draw the connections one by one in the panel above the button. Depending on how many connections are found, this step might take a long time therefore it is recommended to always first visualize the strongest 60 connections.

**4.4 Export figure**

Allows the user to export the figure in HD with 1200 dpi and resolution of 1920x1080 as .tiff file.

**4.5 Select channel**

This button will prompt to select a channel and will only visualize the connections associated with the selected channel. Either the selected channel is connected to other channels or other channels are connected to the selected channel.

**5. Spike sorting panel**

**5.1 Load spike waveforms**

Click to load in the spike waveforms of the detected spikes in the unsorted waveforms panel. After loading in the waveforms, it is possible to look at the spike waveforms of each channel by using the pull-down menu beneath the panel (figure 14(5.11)). *This function will not work if the dataset does not contain any spike waveform data.*

**
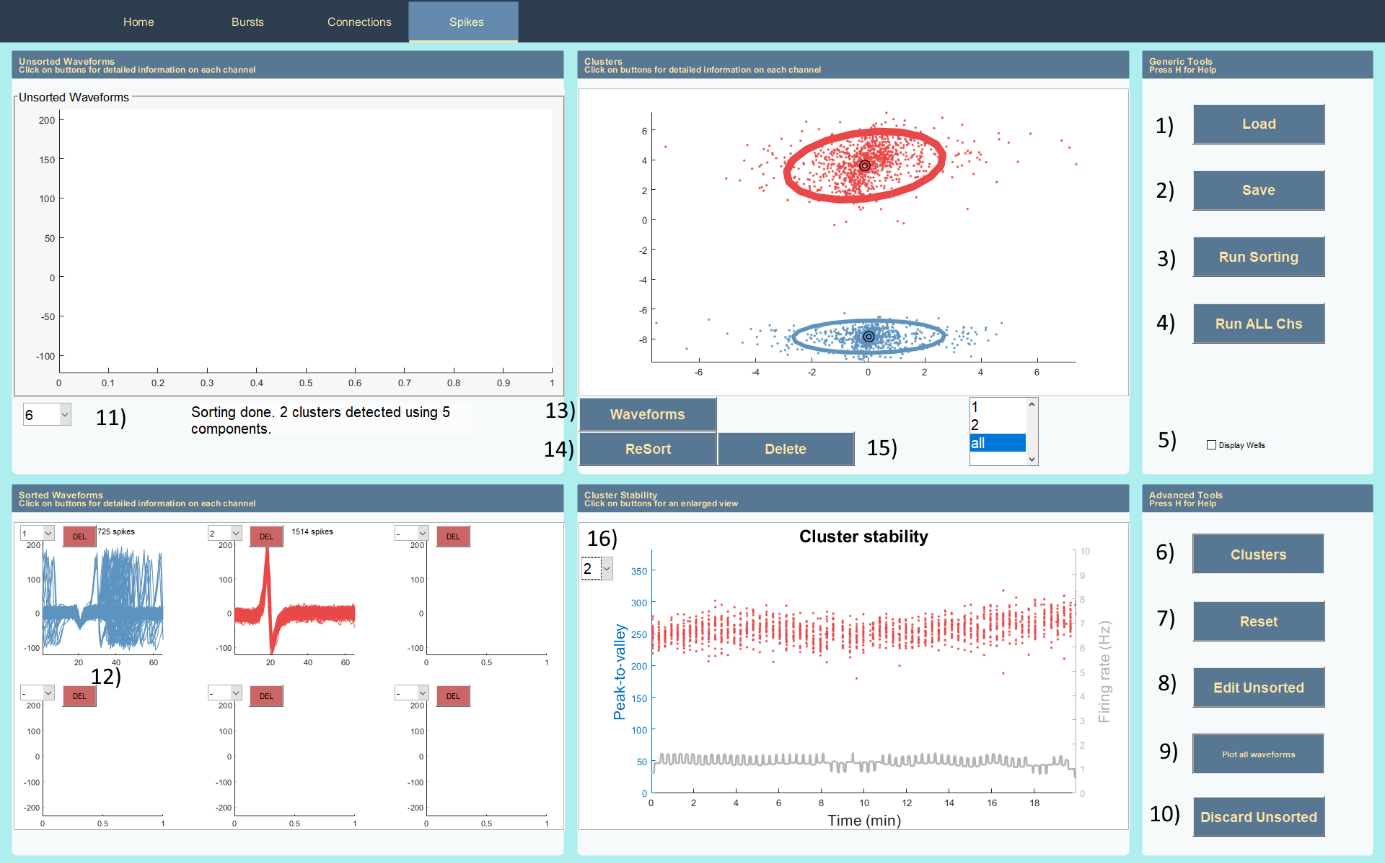
**

***Figure 14.*** *Spikes sorting screen. 1) Load Spike waveforms, 2) Save Spike sorted waveforms, 3) Run sorting, 4) Run all channels, 5) Display Wells, 6) Clusters, 7) Reset, 8) Edit unsorted 9) Plot all waveforms, 10) Discard unsorted, 11) Unsorted Spikes Waveforms, 12) Sorted Spike Waveforms, 13) Waveforms, 14) Resort, 15) Delete and 16) Cluster stability.*

**5.2 Save spike sorted waveforms**

After the spike sorting process is finished, the different detected clusters and the associated spike waveforms can be saved.

**5.3 Run sorting**

Starts the spike sorting process for the current selected channel. After the spike sorting process is finished, the different sorted spikes are displayed in the sorted waveforms panel and several new buttons will become available.

**5.4 Run all channels**

Starts the spike sorting process for the all the channels that have spike waveforms. The algorithm will go through each channel one by one therefore this can take a while before it is finished depending on the number of spikes.

**5.5 Display wells**

Normally the spike sorting process will take place channel by channel. However, ticking this checkbox, all the spike waveforms will be taken together per well and the user can perform spike sorting on all the spikes in a single-well.

**5.6 Clusters**

Visualizes the detected clusters in the cluster panel, which allows for modification of the detected clusters and opens 3 new buttons (Waveforms (5.13), ReSort (5.14) and Delete (5.15)).

**5.7 Reset**

Removes any modifications done after the sorting.

**5.8 Edit unsorted**

When this button is pressed, all the unsorted spike waveform still in the unsorted waveforms panel are one cluster. This allows the user to combine clusters that should belong together. For example, after the sorting process was finished, the user noticed two cluster that are very similar in the sorted spike waveform panel (5.12)). The user can delete these two clusters by using the delete button within the panel and these waveforms will be moved in the unsorted waveforms panel (5.11). By using this button, the spike waveforms within the panel will be combined to form one cluster.

**5.9 Plot all waveforms**

By default, only up to 1000 waveforms are plotted however all waveforms can be plotted with this button.

**5.10 Discard unsorted**

Removes any waveforms that are in the unsorted panel. This action cannot be undone using the reset button.

**5.11 Unsorted spikes waveforms**

After loading in the spike waveforms, the waveforms will appear in this panel and the user can view all the spike waveforms for each channel.

**5.12 Sorted spike waveforms**

After the sorting process is finished, the spike waveforms will disappear from the unsorted waveforms panel (5.11) and appear sorted with unique colours for each cluster. By using the delete button next to each spike waveform, the user can send the spike waveforms back to the unsorted waveforms panel if the user does not agree with the sorting.

**5.13 Waveforms**

Allows for switching between 3 types of plots in the cluster panel. The default is the spike waveforms, a scatterplot of 2 dimensions in the clustering space and, lastly, a line plot of all 5 dimensions in the clustering space.

**5.14 Resort**

Clustering of selected clusters is repeated, and the result is a merged model which allows the unselected clusters to remain the same. The clustering space does not change.

**5.15 Delete**

Selected clusters’ Gaussian are deleted and their associated waveforms are re-distributed to the remaining Gaussians following the same rule used during the clustering process. The Gaussian with the highest probability determines where the waveforms belong to.

**5.16 Cluster stability**

Displays the peak-to-valley distance of the cluster that is selected together with the firing rate which will allow the user to see the stability of the cluster over time.

**6. Neuro endpoints and definitions**

The “Neuro endpoints” button (Figure 5.12) allows the user to automatically extract 20 different endpoints that can be used to determine the status of the recorded neuronal cultures. These include the number of Active Electrodes, Mean Firing Rate, Ratio of Median ISI over Mean ISI, Mean ISI, Mean Coefficient of variation of ISI, Mean Burst Rate, Mean Spike Frequency in Bursts, Mean Absolute deviation (MAD) of the spikes in bursts, Mean Isolated Spikes, Mean Burst Duration, Mean Inter Burst Interval, Mean Burst Count, Mean Coefficient Of Variation of Inter Burst Interval, Mean Network Bursts, Mean Network Bursts Fire Rate, Mean Network Bursts Duration, Mean Network Bursts ISI, Mean Network Bursts Inter Burst Interval, Mean Amount of Connections, and Synchronicity. A description of the Neuro endpoints can be found in table 1. The analysed data is exportable as an excel table for further data manipulation or creating graphs. Note that for multiwell data, the neuro endpoints were calculated by first averaging across the active electrodes in each well to get a single value per well. As explained in the publication, the idea is that these multiple different standardized endpoints can be used to create the electrophysiological profiles. Although to date, there are no standards for how a diseased culture electrophysiological profiles should look, there is evidence that suggest that it is possible to create these electrophysiological profiles for different disease phenotypes.

**Table 1:** *List of calculated ‘Neuro endpoints’ and their definitions.*

| **Endpoints** | **Descriptions** |
| --- | --- |
| Active Electrodes (n) | Electrodes are considered active if they have at least a firing rate of higher than 0.1 Hz |
| Firing Rate (Hz) | Firing rate is calculated by counting all detected spikes in one electrode and divide it by the recording time. The mean firing rate is calculated by averaging the firing over all the active electrodes. |
| Ratio of Median ISI over Mean ISI | Obtained by diving the Median ISI values in each active electrode by the mean ISI values and then the average is taken over all the active electrodes |
| Inter Spike Interval (s) | The time between spikes is calculated and averaged per electrode after which the average ISI per electrode is averaged over all the active electrodes |
| Coefficient of Variation of ISI | Calculated by taking the ISIs for a single-channel and then divide it by the standard deviation of the ISIs. Then the average is taken over all the active electrodes. |
| Single-Channel Burst Rate (bursts/min) | Calculated by counting all the detected single-channel bursts in an electrode and dividing them by the total recording time. Then the average is taken over all the active electrodes. |
| Spike Frequency in Bursts (Hz) | Calculated by counting the total amount of spikes per detected single-channel burst, then divide it by the total. Next averaged across all bursts recorded on a single-channel and diving the number of spikes by the duration of the burst. |
| Mean absolute deviation (MAD) of the spikes in bursts | Calculated by calculating the mean number of spikes in bursts and then subtracting the number of spikes in each burst by the mean value. After this value is divided by the standard deviation of the number of spikes in bursts. This is done for each active electrode and then averaged over all the active electrodes |
| Isolated Spikes (%) | Calculated by dividing the number of spikes in bursts by the total amount of spikes in each active channel. Then it’s averaged over all the active electrodes. |
| Single-Channel Burst Duration (s) | Duration of each burst is summed up in each active electrode and then divided by the amount of single-channel bursts in each respective active electrode and then the average is taken. |
| Single-Channel Interburst Interval (s) | The time between each detected single-channel bursts is summed per channel and divided by the number of elements. Then the average is taken over all active channels. |
| Single-Channel Burst Count (n) | The total amount of bursts detected per channel after which it is averaged over all active electrodes |
| Coefficient Of Variation of single-channel Interburst Interval | Calculated by taking the mean of the IBIs and then divide it by the standard deviation of the IBIs per electrode and then the average is taken over all the active electrodes. |
| Network Bursts (n) | Network bursts are calculated by using the detected single-channel bursts. Using several fine tunable parameters network bursts are detected in the whole array. |
| Network Bursts Duration (s) | The total duration of each detected network bursts averaged over the total amount of network bursts. |
| Fire Rate of spikes within Network Bursts (Hz) | The firing rate of the spikes inside of the network bursts averaged over all the detected network bursts. |
| Network Bursts Inter Spike interval (s) | The ISI between the spikes within network bursts averaged over all the detected network bursts. |
| Network Bursts Interburst Interval (s) | The total time between each detected network burst averaged over all the detected network bursts. |
| Functional Connections | Calculated by using the CFP method reported in Lefeber *et al*., (2007)^2^. After applying the Nelder-mead simplex method, several values are extracted such as the peak values. Based on these peak values (m) a connectivity matrix is made. |
| Spike Train Synchronicity | Calculated by using the ISI distance method reported in Kreuz *et al*., (2007)^3^. The method calculates the similarity between spike trains by using the ISIs and based on the instantaneous fire rate a ratio is calculated between the ISIs of two spike trains which is normalized and averaged over time resulting in a value between -1 and 1. The lower the value the less synchronous the spike trains are and vice versa. |

**7. *MEA-ToolBox* output and definitions**

The main output of *MEA-ToolBox* after the analysis are .mat files. There are in total 68 or 69 variables depending on if the dataset is from a multiwell or a single-well.

**Ans90** is a cell that contains the names of the channels, and this variable is used to plot the names on the x-axis when the user wants to see the heatmap.

**Bab** is a table variable that contains the number of channels that are inactive.

**Bak** is a table variable that contains the number of channels that are active.

**Bal** is a table variable that contains the names of the channels that are active.

**Bap** is a table variable that contains the names of the channels that are inactive.

**BI** is a table variable that contains the burstiness index.

**Bincountssec** is variable that contains information about spike timings for each channel used to create the movie of the spike activity of the well. Each row represents a channel, and the spike data was binned into bins of 30 ms.

**Binranges4567** is a variable that contains the bin ranges used to create the bincountssec variable for the movie.

**BLog** is a variable that contains the amount of single-channel bursts detected in total using the log ISI method.

**BLogSEM** is the variable that contains the standard error mean of the total amount of single-channel bursts detected using the log ISI method.

**BNeuro** is a variable that contains the amount of single-channel bursts detected in total using the max interval method.

**BNeuroSEM** is the variable that contains the standard error mean of the total amount of single-channel bursts detected using the max interval method.

**Burst6** is a cell that contains the single-channel bursts detected with the max interval method. Each row represents a channel and within each cell there are also cells and each of those cells represent the individual detected single-channel burst with their associated spikes(times).

**Burst6indx** is a cell that is the same as the variable burst6 but instead of the spike times it contains the indices of the spike times.

**Burst7** is a cell that contains the single-channel bursts detected with the log ISI method. Each row represents a channel and within each cell there are also cells and each of those cells represent the individual detected single-channel burst with their associated spikes (times).

**Burst7indx** is a cell that is the same as the variable burst7 but instead of the spike times it contains the indices of the spike times.

**Burstview2** is a cell that contains all the indices of the start of the single-channel bursts per channel.

**Burstview3** is a cell that contains all the indices of the end of the single-channel bursts per channel.

**Burstview19** is a cell that same as burstview 2 however from each value 1500 was subtracted.

**Burstview20** is a cell that same as burstview 3 however from each value 1500 was added (a check was performed to ensure that the indices will not go out of bounds).

**Connections** is a variable that contains the total amount of functional connections detected in a well.

**Electrodecount** is a variable that contains the number of electrodes per well. This variable does not exist if the data is a single-well with 60/120 electrodes).

**Exburst** is a cell that has a struct in each row. Each represents a channel and the structs contains information about how many bursts are detected in said channel, the duration of said bursts, how many spikes are in each burst, the fire rate of the spikes within the bursts, the average interspike interval of the spike within the bursts, the standard deviation of the interspike interval of the spikes within the bursts and the interburst interval (these single-channel bursts were detected using the maxinterval method).

**Logburst** is a cell that has a struct in each row. Each represents a channel and the structs contains information about how many bursts are detected in said channel, the duration of said bursts, how many spikes are in each burst, the fire rate of the spikes within the bursts, the average interspike interval of the spike within the bursts, the standard deviation of the interspike interval of the spikes within the bursts and the interburst interval (these single-channel bursts were detected using the logISI method).

**filteredData1** is a variable that contains the voltage trace data used for plotting (y data). The row represents the channels.

**Fs** is a struct that contains information about all the parameters that the user can adjust in the beginning before the analysis starts. There are 8 structs pertaining to spike detection, single-channel bursts detection network burst detection and more.

**HITS** is a cell that contains two columns. The first columns contain the channel names, and the 2^nd^ column contains how many spikes were detected in those channels.

**HITSSEM** is a variable that contains the standard error mean of the total spike count.

**Imax** is a variable that contains the channel that was the most active.

**Imaxch** is a variable that contains the names of 10 channels that were the most active in the well.

**Imaxch2** is a variable that contains the names of 5 channels were 6^th^ to 10^th^ most active in the well.

**INDEX1** is empty variable that contains the selected channel by the user during the use for the GUI.

**ISI58** is a cell that contains the interspike intervals of all channels. Each row represents a channel.

**Joost** is a variable that is either a zero or a 1 which serves a flag to tell the toolbox that we have a data set that only contains spike times and no voltage trace data.

**Multiwell1** is a variable that is either a zero or a 1 which serves as a flag to tell the toolbox that we have a multiwell dataset (when both joost and multiwell1 are 1 then the toolbox knows that the dataset is a CSV file).

**Layout** is a variable that contains the layout of how the channels are organised. If there is a channel at specific location than the channel is represented by the number of spikes detected in that channel. If there is not channel located at that location it will be represented as a NAN.

**Looo12** is the variable that contains the total amount of connections that is the same as the variable connections, but this variable is reserved for the GUI to change so it doesn’t overwrite the connections variable.

**M** is a variable that contains the peak values of the connections between channels. If there are no connections found, then the value is zero or empty.

**M111** is a variable that contains the timings of the spikes that were detected that had a positive peak.

**M2** is a variable that contains information about the number of detected spikes on each channel and when they occurred. The rows represent the channels.

**M222** is a variable that contains the voltage values of the spikes that were detected that had a positive peak.

**M66** is a variable that contains the timings of the spikes that were detected that had a negative peak.

**M88** is a variable that the same as the M2 variable however instead of the spike timings it contains the voltage values of the peaks of the spikes.

**M99** is a variable that contains the voltage values of the spikes that were detected that had a positive peak.

**Negunits** is a variable that contains information about the total amount of spikes detected that had a negative peak in a well.

**NegunitsSEM** is the standard error mean of the number of spikes detected in the whole well with a negative peak.

**Posunits** is a variable that contains information about the total amount of spikes detected that had a positive peak in a well.

**PosunitsSEM** is the standard error mean of the number of spikes detected in the whole well with a positive peak.

**Networkburstttt** is struct variable that contains information about the detected network bursts. There are in total 9 fields, starttime, endtime, amount, duration, spikesfr_in_nbursts, nburst_rate, ISI, IBI and CVIBI. The starttime contains all the start times of all the detected network bursts. The endtime contains all the end times of all the detected network bursts.

**newM2** is a variable that contains the layout to create the connectivity maps. This variable is identical to the layout variable however this was done in order to not to overwrite the original layout variable.

**Noiseall18** is a variable that contains the indices of the base line noise that will used to detect the spikes (x axis).

**Noiseall2** is variable that contains the indices of the base line noise that will used to detect the spikes
(y axis).

**OFFSET** is a variable that contains the offset values estimated during the calculations for the conditional firing probability. The rows and columns represent channels. For example, the offset value between channel 30 and 60 can be found in row 30, column 60.

**OPP** is a variable that contains the surface area values of the peak estimated during the calculations for the conditional firing probability. The rows and columns represent channels. For example, the OPP value between channel 30 and 60 can be found in row 30, column 60.

**RMS7** is a variable that’s contains all the threshold values per channel used to detect spikes.

**Selecteddata** is variable that contains the name of the analysed file.

**Parts** is a variable that contains in how many parts the file should be split in. this number is equal to the number of wells there are in the dataset. This variable only exists if the dataset is from a multiwell.

**Spikeform3** is a variable that contains the all the spike waveforms per channel of only the spikes detected with a negative peak. Each row presents a channel.

**Spikeform4** is a variable that contains the all the spike waveforms per channel of only the spikes detected with a positive peak. Each row presents a channel.

**Supa** is a variable that contains the cross correlations of all the channels. Each row represents a channel. This variable is used to calculate the conditional firing probability.

**T** is a table variable that contains information about each individual channel. There are 11 columns that contain the amount of spikes, the average inter spike interval, the median inter spike interval, the standard deviation of the inter spike interval, the amount of single-channel bursts detected using the max interval method, the amount of single-channel bursts detected using the log isi method, the fire rate, the average instantaneous firing rate, the average coefficient of variation of the inter spike interval, the average 2^nd^ coefficient of variation of the inter spike interval and the threshold value used to detect the spikes. The table is organised based on the number of spikes. So, the channel with the most spikes is on top.

**Textstrings** is a variable that contains the names of the channel which is used to plot the connectivity maps

**Timesss** is a variable that contains the total duration in seconds of the recording.

**Tt** is a variable that contains the time values from the origin to the timepoint of the peak estimated during the calculations for the conditional firing probability. The rows and columns represent channels. For example, the time value between channel 30 and 60 can be found in row 30, column 60.

**Unitpersecond** is a variable that contains the binned spikes per channel to plot a spike histogram.

**X2** is a variable that contains times information used for plotting (x data).

**Xpoints** is the variable that contains x axis data in order to plot the spike raster plots.

**Ypoints** is the variable that contains the y data in order to plot the spike raster plots.

**8. Design of *MEA-ToolBox* and instructions for modifying the code**

**For code modification, users should be familiar with MATLAB programming (Indexing, for/if/while loops, variable types, etc.). For navigation purposes, the instructions below use the line numbers corresponding to the line numbers in the code in this explanation to assist with navigating the code. Lastly, the names of the scripts will be in bold and suggestions in italic.**

Currently there are 2 types of data formats that is compatible with the *MEA-ToolBox*, i.e. HDF5 and CSV file format. The HDF5 file format can either contain voltage trace data or only spike data without the voltage trace data using a different spike detection program or algorithm. The CSV file format can only contain spike timestamps and not voltage trace data. For the exact details on how the data should be organised in these two specific data formats please see 1.2 (Getting started) or 1.3.1.8 (Only spike time stamps data format).

**Starting up the *MEA-ToolBox***

**8.1 Main_Menu_Toolbox.m**

The user can start the *MEA-ToolBox* by calling the script **Main_Menu_Toolbox**. The user will be presented with a GUI containing 4 pushbuttons each containing a callback that can be found in **Main_Menu_Toolbox**. In the openingfcn there are three lines (56-59) which makes the GUI full screen on start-up and assigns the name ‘ha’ to the axes of the GUI in which the animation is plotted into. The user can change the animation of the GUI by changing either the files in the Animation folder or the name in line 111 to another name.

The way the toolbox works is that the user must press the Set Parameters button first before the analyse and load button becomes available. Once pressed (pushbutton 4) the script called **parameters** will be called and a new screen will be shown containing a multitude of parameters that the user can change. For more details on these parameters please see 1.3.1 (Set parameters). The openingfcn of this script contains all the default values that will be used if the user does not change anything. *The user can add new default values if the user wants to add a different method here.* There are several uitables within this script matching the different analysis methods parameters used in the *MEA-ToolBox* and the user add or remove parameters here. Or add new parameters as well by creating new uitables. Ignore pushbutton3 within this script (parameters) for now as it is still a work in progress. Lastly there are 3 checkboxes that are used to check and indicate what kind of data the user will use, and which single-channel burst method the user wants to use. *The user can add additional checkboxes here to check for different data types or if the user added a new method, then the user can add a checkbox here for usage. If the method is an alternative to the exiting methods such as a different single*-*channel burst detection method, then the user can use the existing SCBflag. 1 and 0 are used for the max interval and logISI method respectively. If the user adds new parameters, please make sure to add it to the fs structure as this variable is used to store all the parameters used in the analysis.*

Once the parameters are set and the changes are saved, the analyse (pushbutton1) and load buttons (pushbutton3) will become available. Within the callback for pushbutton1 the variable flag is used to distinguish between if the data is only just spikes in a HDF5 file format from the rest (the other types are distinguished in the script called **MEAToolboxV3**). The user could use t*his flag here to distinguish between different data inputs if the user decided to add one.*

Loading data or the callback for pushbutton3 will only accept .mat files as input which the output file of the *MEA-ToolBox*. The output file of the *MEA-ToolBox* will be the exact same name as the input file containing 67/68 variables. For more detail, please see 7. (*MEA-ToolBox* output and their definitions). Here there are two flags that are used to distinguish between multiwell, single-well (HDF5 with voltage) and CSV files (only spike times) in order to use the correct GUI (**multiwell** is used for multiwell data that originated from HDF5 files, **CSVmultiwell** is used for multiwell data that originated from CSV files and lastly **MEA_Data_Plots2** is use for single-well data that originated from HDF5 files). *If the user added a different input file format and created a different GUI to match this file format, then the user can add another flag to open the correct GUI matching with this input format.*

**Preparation to analyse with *MEA-ToolBox***

There are three main scripts that contain all of the analyses described in the manuscript. The 3 main scripts are **MEAToolboxV3**, **justspiketimes** and **Multiwell_mainbody**. The **justspiketimes** is the script that is used for data that only contains spike times in the HDF5 file format. Please see 1.3.1.8 (Only spike time stamps data format) for more details about the input format. All of these share the same methods that are used to analyse the data as described in the manuscript. In order to make this concise we will go through **MEAToolboxV3,** and we will highlight any differences in the other scripts.

**8.2 MEAToolboxV3.m**

**Verification of the data format (lines 7 – 137)**

The first section (lines 7 – 79) contains multitude of global variables used across multiple scripts and in the GUI. If the user adds a new variable that needs to be used across multiple scripts and the GUI, then the user can consider adding this here as a global variable. In the next section (80 – 90) the user is asked to select the folder in which the files are located to be analysed. Of note is that if the user wants to add another input file, then it is of importance to add this file extension in line 88 together with the other file extensions. Following this is the section (92 - 120) to check what kind of data the HDF5 or CSV files are. The first check happens in line 109 – 113 where we determine if the file is a CSV file. If so the *MEA-ToolBox* will run the script **CSVfiles** and set the variable electrode count to 16. *The user can change this value to match the number of electrodes in each well in the dataset.*

Lines 115 – 121 can be ignored as they are still a work in progress to account for a problem that does not exist yet. Continuing onwards since the file is not a CSV file the *MEA-ToolBox* will assume it’s a HDF5 file. What is important is that the channel information is assigned to Data1 in line 131. Channel information in this case is the name of the channel. For example, channel 1 is named A1. The assumption we make here for now is that index 1 of this variable is channel 1 of the dataset. This assumption changes depending on if the dataset is from a multiwell or from a single-well. *Therefore, if the user wants to add another type of input file format it is important to assign the channel information to Data1.*

If the data is not a CSV file, then we continue with the 2^nd^ check that happens in lines 130 – 137. This will check if the data set is from a multiwell. This detection is because HDF5 files generated by the multichannel systems (MCS) hardware contain a field that states what kind of MEA layout it is. If the user is adding a new type of input file format, then the user can use the variable ‘checkmulti’ to indicate if the data is from a 24 multiwell by reading the specific field that contains strings that indicate it is a multiwell dataset or the user can just assign the string 24W. If the data file was from a 24 multiwell then the electrode count is set to 12 and the script **Multiwell_Mainbody** will be called. *The user can use the if statement to add more conditions to account for a more unique channel distribution per well or if the user has added another input method for a multiwell dataset then the user can call their method within this if statement.* Continuing the *MEA-ToolBox* will assume that the data file is from a single-well MEA.

**Extracting voltage trace data and channel information (lines 141 – 224)**

Lines 141 - 143 can be ignored as it refers to something that does not yet exist. What is important here is that the channel data is assigned to the variable Data2. The reason why we have the try and catch statement is because sometimes the location of the channel data is different. *This section is only important to change if the user has added a new input file format.* *Please make sure that the channel data (voltage trace data) is assigned to Data1 in the format channels * samples.* Data1 should be a matrix in which a row contains all the samples recorded per channel and each column represents the channels. If the orientation of Data1 is reversed, then it will be corrected in lines 158 – 168. On line 170, the variable multiwell1 is set at zero indicating that the data file is from a single-well MEA. Next the lines 172 – 186 can be ignored as they are still a work in progress. Lastly, Data2 is converted into double precision on line 188 because certain inbuilt functions of MATLAB do not accept integers as an input. On line 189 the Channel data is divided by a certain factor which is unique to the MCS hardware. This conversion factor is not present in CSV files.

Lines 203 – 208 is used to account for files that are too short for analysis. If the dataset is too short, then it will be stored in a new folder and continue with the next datafile. Lines 210 – 224 is purely meant to remove any excess channels of the single-wells that have 60 channels. Some recording software will include the reference electrodes. For example, the older normal single-well with 60 channels from MCS has 64 channels including the reference channels. We have noticed that this is not always the case however if this is not accounted for it will have some influence on the network burst analysis.

**Filtering the data (lines 226 – 267)**

Lines 226 – 234 create the time vector for the voltage trace data in Data1 for plotting purposes. From lines 242 – 263 the voltage trace data in Data1 is being passed through a high pass Butterworth filter in order to remove all the low frequency components. The cut-off frequency is determined in line 242 and we are using a built-in MATLAB function called butter to create the filter. The function butter requires three arguments, the filter order, the cut-off frequency and what kind of filter it is (high, low, broadband or stop). After which a pre-allocation step is performed in order to try and speed up the process of applying the filter on each channel in lines 246 - 256. Lastly, the filter is applied to each channel one by one in lines 258 – 261 and assigned to a new variable called filteredData1. *The user can add his/her own filters in this section and apply it to Data2.*

**Determining baseline noise (lines 274 – 447)**

In order to determine the baseline noise, we are using a method from the literature ^1^. This method starts by splitting the data into smaller bins. Each bin is 50 ms long in the original method. Line 275 is used to indicate how many samples are in one bin in order to reach the correct bin size. For example, we have a sampling frequency of 20 kHz, and we want to split the data in bins of 50 ms. Then the n on line 275 will be 1000. Now that we know how long each bin should be, we must split the data in the correct number of bins. In order to do this, we first pre allocate a matrix that matches the size of the final matrix in line 277 and fill it with nan values. We know the final size of the matrix because each bin should be the size of 1000 samples. Let’s say we have a recording containing 6 million samples. This means that the final matrix size for one channel would be 1000 * 6000. For 6 channels this would be 1000 * (6000*6). Then we assign the correct values to each nan value in line 278. We switched the orientation in line 276 purely so we could navigate easier through the matrix. Now we have a variable called dummy that contains the voltage trace data split into 50 ms bins per channel. We also do this for the time vector in lines 281 – 283 for plotting purposes so we can visualize the detected baseline noise per channel. The organisation of the dummy variable is not optimal as all the 50 bins of all channels are next to each other. This means that if we take the previous example channel 2 would starts from column number 6001. In order to separate these bins per channel for a better overview the lines 288 to 297 exist. The new variable called split has all the voltage trace data split in 50 ms separated for each channel in a cell. If we use the same example form earlier then we would see a 1000 x 6000 matrix in one column in each row up until the number of channels.

Next, we make use of a built-in MATLAB function called normfit which allows us to estimate the parameters from a normal distribution. We obtain the standard deviation for every 50 ms bin for each channel and store it in a cell called pdyt. Once again if we use the previous example this would mean that we have 6000 standard deviation values for each channel. Lines 315 – 321 exist to make the organisation of the values the same. Each cell in the variable pdyt will have their standard deviation values aligned as a column. Lines 326 – 331 calculate the threshold that will be used to detect the baseline noise. Here we set the threshold to the average value of the standard deviations per channel. Lines 336 – 352 compares each 50 ms bin in each channel to see if the values are below the threshold calculated before. If they are lower than the threshold then they will be stored in a new variable called spanLength. After which line 348 will search for a section of consecutive 50 ms bins that are below the threshold. The default value is 2 seconds. So, the algorithm will have to find at least a 40 consecutive sections. If this section is found it will be stored in noiseall99, otherwise it will be left empty. Please note that the numbers stored in noiseall99 are index values and the algorithm will continue until a section is not below the threshold value. This means the length of the sections will not always be 40 but can be longer. If multiple sections are found, then these will all be stored. Lines 355 - 366 are a double check-up to ensure that Data1 contains the channel information (channel 2 is A2 for example). This is because sometimes this information is stored in another field.

Lines 368 – 370 are meant to fill the empty spots of the variable noiseall99 with a vector of equal size to one binsize. This was done to ensure that the later algorithms didn’t have to account for an empty cell and all the code will work. Later, we will disregard the empty channels. Lines 375 – 387 will attempt to search if there are two 2 seconds windows that can be used to calculate the baseline noise. One additional condition is added which is that the two seconds windows must be at least 12 seconds apart from each other which is seen in line 378. The result is a cell with 1 or 2 rows of indices that will be used as the baseline noise per channel. The total length of each row should match with the window you selected. For example, each bin is always 50 ms and if you wanted a time window of 2 seconds then the total length of each row is 40. Lines 391 – 397 are to ensure that the organisation in each cell is the same. Lines 402 – 404 are to round up the numbers in noiseall99.

Lines 407 – 412 are to correct for the fact that sometimes if the code in lines 375 – 387 finds a 2^nd^ time window that is near the end of the recording then it can happen that the full length of the section is not equal to time window indicated by the user. For example, in this case it’s 2 seconds and let’s say the recording is 60 seconds long. The code finds a suitable 2 second window from 58 to 60 seconds. However, there is sometimes a problem that arises that the actual recording is not really 60 seconds but 59.8 seconds or 60.1 seconds. We have encountered this several times we think it’s a hardware issue because the recording software should stop at the user indicated times, but it sometimes doesn’t. Therefore, in order to combat this issue, we check if the last index of the windows we found in noiseall99 is the actual last index in the recording. If it’s not, then we change the last index to match with the actual recording. Lastly, there is a final check to determine if the index values in noiseall99 are values that are within range of the data that we split in 50 ms bins in the variable Split.

Lines 426 – 433 are to extract the actual values from the variable Split using the index values from nosieall99 to visualize the baseline noise that is used for the spike detection. Each bin is 50 ms containing the number of samples matching this duration (for example if the sampling frequency 20 kHz, then 50 ms is equal to 1000 samples). We first use the index values to extract the voltage values and store them in noiseall2. Note in our example this would mean that the length of one 2 second window is equal to 40000 samples. We do the same for the time values in lines 436 – 443 and store them in noiseall18. *Alternatively, the user can forgo calculating the baseline noise and instead just calculate the standard deviation of the whole filtered signal in Data2 and set the threshold based on that. Important here that the user needs to make sure that the threshold values per channel needs to be stored in a variable called RMS7.*

**Calculating the Root Mean Square (lines 451 - 490)**

Now that we have determined the baseline noise, we can proceed to calculate the RMS used for spike detection. Lines 450 – 455 squares all the points of the baseline noise we found in noiseall2 and stores it in RMS19. Lines 459 – 469 will take the average of the squared values and store it in the variable new. If there were two windows, then there will be one value for each window. Lines 474 – 476 will square the values in the variable new. Lines 478 – 480 will multiple the number(s) with the multiplier chosen by the user to set the threshold for spike detection. Lines 482 – 484 will take the average if there were two values to serve as the threshold to detect spikes and store all the threshold values in a new variable called RMS7*. The user can change the method used to set the threshold for spike detection. For example, instead of using the RMS, the user can change it to standard deviation.*

**Spike Detection (lines 498 - 586)**

Lines 500 – 506 are used to detect the spikes using a MATLAB built-in function called findpeaks. Using this function, we can find peaks in the filtered signal that meet our criteria. There are three options that we are using but the most important one is the MinPeakHeight which allows us to find peaks above a threshold. The threshold values we use are in the variable RMS7. We can also add 2 extra criteria that our spikes need to fulfil. For example, the spikes need to be a certain distance from each other. Or the spikes need to have a minimum amplitude before it’s considered a spike. The output of the findpeaks function are the voltage values and the time values associated with each detected spike. These are stored in a cell called allpeaks. The first column are the voltage values of each spike per channel and the 2^nd^ column contains all the time values for each spike per channel. These spikes that are stored in allpeaks are all ‘positive spikes’, meaning that these spikes come from the positive side of the signal. Lines 511 – 516 counts the number of positive spikes that are detected.

Lines 522 – 530 will detect the negative spikes by inverting the filtered data in line 522. Similarly, to the positive spikes the voltage and time values are stored in all1peaks and the voltage values are in the first column and the time values are in the 2^nd^ column. Lines 534 – 539 will calculate the number of negative spikes per channel. Lines 543 – 546 will put the highest number of spikes detected on the positive side in the first column in the variable called maxiu1. The highest number of detected spikes on the negative side is put in column 2. The reason for these 2 values is because then we can put all the detected spikes (both positive and negative) in one matrix as seen in lines 551 - 564. The channels that have not as many spikes as the channel with the highest number of spikes will be padded with nan values to make them equal in length so we can put them in a matrix. For example, we have 1000 spike as the highest number of spikes on the positive side and 1000 on the negative side. The result is that we will have a matrix with the size of 60 * 2000 (the first value represents the number of channels). We do this for the voltage values and store them in a variable called ToT2. Each row represents a channel, and it contains the voltage values for the detected spikes otherwise it is a nan value. We do the same for the time values for each detected spike in lines 570 – 584. The time values for each detected spike are stored in ToT.

**Preparation for artefact detection (lines 591 – 683)**

Lines 591 – 596 reorganises the spike data in positive and negative spikes and stores the voltage and time values for both in separate cells (M111 and M222 for negative spikes, M66 and M99 for positive spikes). Lines 601 – 607 separates the number of spikes back to a channel-by-channel basis however now all the positive spikes negative spikes are combined, and the nan values are not included. The time values for each detected spike are stored in a variable called M2. We do the same for the voltage values and store them in the variable M88 in lines 609 – 615. Lines 619 – 631 will sort the spikes based on their timing in each channel. We use a MATLAB built-in function called sort. This function will not only sort the values but also give us the indexes how the new order was created. Using these indexes, we also order the voltage values of the detected spikes in M88. We also order the separated spikes in M66 and M99 for the positive spikes and M111 and M222 for the negative spikes. Lines 634 – 680 are to ensure that there are no nan values in the variables M2, M88, M66, M99, M111 and M222.

**Artefact detection (lines 698 – 751)**

Lines 699 – 721 are used to check if the detected spikes occur within 1 ms from each other (both negative and positive spikes) and if so then the spike that has the lowest amplitude will be removed. We find the indexes of spikes that occur within 1 ms from each other in line 702. Then we use the index to find the associated voltage value of the spikes that occur within 1 ms from each other in lines 708 and 709 and compare which is higher. The spike that has the highest amplitude value will be kept and the lowest one will be removed. This is done channel by channel. Lines 733 – 741 are used to check if the fire rate of each channel is higher than the minimum fire rate for a channel to be considered active. Lines 744 – 750 are used to calculate how many spikes there are per channel, and they are stored in Truehits. *The user can add an alternative artefact detection method based on the amplitude of the spikes here if desired.*

**Preparation general data (lines 755 – 762)**

Lines 755 – 760 are used to calculate some basic data such as the number of spikes and the maximum and minimum amplitude values per channel.

**Spike Waveforms (lines 769 - 1000)**

Line 769 is used to calculate how many samples is equal to 1 ms. Lines 770 – 773 is used to create a new cell that contains the voltage trace separated per channel in each cell. Lines 777 – 782 are used to get the indexes of when the negative spike times occur in the recording by using a built-in MATLAB function called intersect and store them in M20. Lines 784 – 791 are used to get the index that corresponds to 1 ms before the negative spikes occur and these indexes are stored in M21 and the indexes that corresponds to 2.2 ms after the negative spikes occur are stored in M22. *The user can change these values to obtain spike waveform that are longer or shorter.* Lines 796 – 842 are used to check the indexes that are found in M21 and M22 do not fall out of bounds. The reason for this is because we simply add or subtract to the indexes as can be seen in lines 787 and 788. What can happen is that if we have spikes that either occur really close to the beginning or end of the recording that these indexes fall out of bounds. In order to correct that we check all the indexes in M22 and M21 to make sure they aren’t out of bounds and if they do we set them to either the first index or the last index of the recording. This means that in certain fringe cases that certain spike waveforms are not 3.2 ms in total duration but a little shorter. Lines 852 – 859 are used to fill in the empty channels or channels with less than 10 spikes with a 1. Lines 863 – 878 is used to obtain the actual spike waveforms by using the indexes from M21 and M22 and indexing in the variable Good. There is one if statement to counter the empty channels that are filled with a one. These spike waveforms of the negative spikes are stored in Spikeform3. Lines 880 – 882 are used to switch the orientation of the data in Spikeform3. Lines 890 – 997 are the exact same as lines 777 - 882 except we extract the spike waveforms for only the positive spikes and store them in the variable Spikeform4.

**Mapping the channel names (lines 1006 – 1206)**

Lines 1006 – 1126 is only used if the data is from a 120-channel single MEA. The correct channel names are stored in the variable channelnames2. Lines 1131 – 1134 are used to check if the number of channels is 64 if so the last 4 channels are removed. With the newer single-well MEA’s the reference channels are not stored anymore so this is probably not needed anymore. Lines 1138 – 1198 is created to assign the correct channel names for a 60-channel single-well MEA. Lines 1200 – 1204 is used to check how many channels there are in the dataset and depending on if it’s a 60 or 120 channel MEA the corresponding variable will be used for the correct channel names. *The user can add a different organisation for the channel names if the user has a more unique organisation of their channels. The channel names organisation here are based on the multi channel systems single*-*wells. Please make sure to save the names in the variable channelIDs.*

**Preparation for spike raster plots (lines 1211 - 1228)**

Lines 1211 – 1216 are used to create one vector and assign the channel numbers to the spikes that occur. For example, there are 100 spikes that occur in the first channel and 50 in the 2^nd^ channel. The output (ypoints) of these lines is a vector in which the first 100 elements are 1’s and the next 50 elements are 2’s. Lines 1220 – 1224 creates one vector (xpoints) that contains all the spike timings from each channel sequentially. So, if we take the previous example, the first 100 spike timings will be from channel 1 and the next 50 spike times are from channel 2.

**Single**-**channel burst detection (Max interval method) (lines 1241 – 1501)**

Lines 1241 – 1246 are used to calculate the inter spike interval (ISI) per channel using a built-in MATLAB function called diff and store them in test2. Lines 1248 – 1250 is used to add index values to the ISI numbers per channel. Lines 1254 – 1258 will remove any channel that has less than 3 spikes. Line 1269 will first find the indexes of when ISI between the spikes is lower than the 170 ms (default value) and store them in idx. Next, we loop through these indexes we found (line 1275) one by one to search for spikes that occur within 300 ms (default) from these indexes that we stored in idx (line 1276). If the spike occurs within 300 ms then we save them in the variable burst 3 and otherwise we break out of the for loop and concatenate the results of burst3 and assign them to burst4 before going to the next element in idx. The output for the nested for loops in lines 1273 – 1290 is a variable burst4 that will contain all the indexes that start with spikes that have an ISI of 170 ms and the following spikes occur within 300ms. The lines 1293 – 1299 is used to check if the number of indexes found are at least higher than 10 (default value). If not, then they are removed. Line 1301 is used to remove any empty cell. Line 1305 is used to create a new vector called NewVector and get the last index from each cell of burst4. Lines 1308 – 1319 is used to check if the last index of the cells of burst4 don’t overlap with each other. If they do overlap that means that the end on the same time which means that they are a duplicate but have a different starting time. So, if they are the same the subsequent cells are removed resulting in the first one remaining behind which is the cell that starts the earliest. Lines 1327 – 1341 are used to check if we should merge the cells or not. This is done by checking the start time of the next cell with the end time of the previous cell and see if the difference is smaller than 200 ms (default value). If the difference is smaller than we merge them together before storing them in burst5. Lines 1346 – 1351 is used to check if the duration of the cell is at least 10 ms (default value) if not then they will be removed. This process is done channel by channel and stored in burst6. All the values stored in burst6 are all indices. Lines 1365 – 1369 converts the indices to actual time values. Lines 1371 – 1480 are used to calculate various single-channel burst metrics before storing them in the variable Exburst. An explanation of these metrics can be found in table 1 of page 23.

Line 1486 can be ignored as it supposed to raise a flag to find a potential error, but it is not used yet. Lines 1489 – 1499 is used to calculate the ISI and converts it into milliseconds.

**Single**-**channel burst detection (Log ISI method) (lines 1519 - 1894)**

Lines 1519 – 1539 are used to create histograms based on the ISI values per channel in which we use equally spaced logarithmic bins. In order to do this, we first calculate the maximum number of bins that are needed to create the histogram by taking the log of the maximum ISI value in a channel in line 1526. After which in line 1527, we create the logarithmic bins by using a built-in MATLAB function called logspace which allows us to create a logarithmically spaced vector between 10^0 and 10^maxWin with a certain number of points. This vector called bins is then used to create a histogram which is further normalized. Lines 1543 – 1552 are used to smooth the log ISI histograms stored in logISI_hist. Lines 1559 – 1596 are used to detect peaks in the smoothed log ISI histograms as can be seen in line 1568. After which we check if there is a peak in the histogram that occurs before 100 ms if so, we save the time of the peak. If there are multiple peaks before 100 ms then we take the peak with the highest ISI value and store it in intraburstpeaks. Otherwise, if there are no peaks found then there are no single-channel bursts that occur in the channel. The other peaks are stored and the associated time value as well. These time value of the peak value stored in intraburstpeaks will be used later to detect the single-channel bursts. Lines 1605 – 1636 are used to determine the interburst interval that will be used to detect the single-channel bursts later. Line 1618 calculates the minimum value between the highest peak ISI value before 100 ms and the other peaks that were found. This minimum value is stored in temp1 and then we calculate how well these peaks are separated which is based on the void parameter which is calculated in line 1620. The first peak that has a void parameter that exceeds 0.7 will be set as the interburst interval. If there are no subsequent peaks, then the interburst interval is set at 100 ms. The interburst intervals are stored in interburstlogISI. Lines 1640 – 1647 combines the found intraburst intervals and interburst intervals for each channel in the variable ISIthh and converts them into seconds because the ISI values from ISI58 were in milliseconds. Lines 1656 – 1664 are used to make sure that if we both have the time values for the intraburstpeaks and interburstlogISI. The most important value is the intraburstpeaks in the 2^nd^ column. If the interburstlogISI is missing, then we set the value to 100 milliseconds.

Lines 1670 – 1675 creates a variable called test2 that contain all the ISI in seconds on a channel-by-channel basis. Lines 1684 – 1796 are used to detect the single-channel bursts using the values in the variable ISIthh. Line 1695 finds indexes of ISI values that are lower than the intraburstpeaks values stored in the 2^nd^ column of ISIthh. These indexes are stored as they are the potential starting points of single-channel bursts. Next, we loop through these indexes we found (line 1695) one by one to search for spikes that occur within a certain time from these indexes that we stored in idx (line 1695). If the subsequent spikes are smaller than the time values in the first column of ISIthh then we save them in the variable burst3 and otherwise we break out of the for loop and concatenate the results of burst3 and assign them to burst4 before going to the next element in idx. So, we use the values from intraburstpeaks as the starting point of a single-channel burst and we use the values from interburstlogISI to add spikes to the potential single-channel burst. Lines 1720 – 1726 are used to remove any potential bursts that are found to be smaller than 4 spikes (default). Line 1728 is used to remove any empty cell. Line 1733 is used to create a new vector called NewVector and get the last index from each cell of burst4. Lines 1736 – 1745 is used to check if the last index of the cells of burst4 don’t overlap with each other. If they do overlap that means that the end on the same time which means that they are a duplicate but have a different starting time. So, if they are the same the subsequent cells are removed resulting in the first one remaining behind which is the cell that starts the earliest. Lines 1754 – 1765 are used to check if we should merge the cells or not.

This is done by checking the start time of the next cell with the end time of the previous cell and see if the difference is smaller than corresponding interburstlogISI value. If the difference is smaller than we merge them together before storing them in burst5. Lines 1770 – 1784 are the same as lines 1736 – 1745 to check if there are duplicates, if so, we remove them and then we assign the detected single-channel bursts to burst7. All the values stored in burst7 are all indices. Lines 1795 – 1799 converts the indices to actual time values. Lines 1801 – 1891 are used to calculate various single-channel burst metrics before storing them in the variable logburst. An explanation of these metrics can be found in table 1 of page 23. *The user can add their own single*-*channel burst detection method here. If the method is also based on the ISIs then the user can make use of the variable ISI58. Other useful variables are M2 and fs. M2 contains all the spikes times in seconds per channel and fs contains the parameters information. If the method has changeable parameters, then the user can add these parameters in fs and let other users change them as well. If the user adds a new detection method, then the user needs to make sure that the output is saved as a separate variable and saved later. Also, what is important for visualization purposes is to have the organisation of the saved output to be like the organisation of burst6/ burst7.* *When the detected bursts are used in the GUI the user can just copy the code used to display the max interval or log ISI bursts by changing the locations that uses burst6/burst7 with the new variables name if the organisation is the same.*

**General data (lines 1901 - 2166)**

Lines 1904 – 1913 are used to calculate the instantaneous fire rate per channel by using the formula in line 1897. Lines 1918 – 1923 are used to calculate the mean instantaneous fire rate per channel. Lines 1934 – 1939 are used to calculate the mean ISI value per channel. Lines 1943 – 1948 are used to calculate the standard deviation of the ISI per channel. Lines 1952 – 1958 are used to calculate the coefficient of variation of the ISI per channel. Lines 1964 – 1999 to calculate the coefficient of variant 2 of ISI per channel using the formula in line 1963. Lines 2003 – 2009 are used to calculate the mean value for the coefficient of variation 2 of the ISI. Lines 2015 – 2019 calculates the number of single channel bursts detected using the max interval method per channel. Lines 2023 – 2027 calculates the number of single-channel bursts detected using the log ISI method per channel. Lines 2030 – 2037 will put the channel names in separate cells if there are more than 120 channels. Lines 2039 – 2048 will calculate several endpoints such as fire rate or the standard error mean of several endpoints such as the number of negative/positive spikes. Lines 2054 – 2079 will calculate the mean ISI, median ISI and standard deviation values of the ISI’s per channel. Lines 2085 – 2098 is to check if there are the same amount of channel names as there are channels with spikes detected and if there are no duplicate channel names. If this is the case, then we change the channel names for numbers. We have noticed that at least for single MEA’s from MCS that they their channels are always organised from left to right, top to bottom. Therefore, we followed this for naming the channels. *The user can change this the names to match with her/his MEA’s layout. Please assign these names to channelIDs and when assigning these names please keep in mind that the rest of the code assumes that the layout goes from left to right first and then from top to bottom. For example, the first row contains 6 channels, and the second row contains 12 channels then you should place the names of the first 6 channels on the first 6 indexes and the 12 channels of the 2^nd^ row will be index 7 to 18 etc.*

Lines 2105 – 2107 are used to make sure that the channel names in channelIDs are strings. If not, then they will be converted to strings. Line 2109 creates a table containing all the information calculated from lines 1904 – 2079. Please keep in mind that this is information for each channel. Lines 2116 – 2121 is used to remove any channel that has less than 10 spikes. Line 2127 is used to sort the table based on the number of spikes detected. Lines 2129 – 2131 is used to remove any rows that has no spikes. Lines 2133 – 2164 is used to calculate the sum of the columns of table T and then assigns this value to the bottom of the table.

**Heatmap generation (lines 2169 - 2400)**

The heatmap is generated based on the detected spikes. The higher the number of pikes the more yellow the area becomes and conversely the lower the number of spikes the bluer they are. Line 2169 is used to create a new variable based on the number of spikes detected which is obtained from table T. Lines 2172 – 2383 is used to determine the MEA layout. If the MEA contains 60 channels, then the layout at line 2364 will be used. Otherwise, the layout in line 2381 will be used. Please note that this corresponds to a 120 channel MEA. Also, at the same time a new variable called HITS will be created. The first column contains the channel names, and the 2^nd^ column contains the number of spikes detected in said channel. *The user can add his own layout by adding an extra else statement. The way the layout works is that we work from left to right and go from top to bottom. The empty spaces between the channels are filled with nan values because these can be ignored later. The reason why we use nan values is so that we can create a matrix of equal lengths. When the user adds his/her own layout please make sure that the variable HITS is also created with the names in the first column and in the 2^nd^ column the number of spikes per channel are stored. Also please make sure that the organisation matches with the variable channelIDs.* Lines 2385 – 2398 are used to create a variable called ans90 to contain the y axis of the heatmap. *If the user wants to change the y axis to different names, then please change ans90 but make sure that everything is in strings.*

**Active/inactive channels (lines 2406 - 2421)**

Line 2046 will calculate the mean firing rate over the whole well. Lines 2407 – 2418 will calculate the amount of silent and active channels but these values are currently not used for now. So, these lines (2406 - 2418) can be ignored for now.

**Zoomed-in single**-**channel bursts (lines 2425 - 2499)**

Lines 2425 – 2429 will assign the either the max interval or the log ISI detected bursts to the variable SCBmethod based on the choice the user made in the beginning (max interval method is default). The user can add a flag here to assign the detected bursts using their method to the variable SCBmethod. Lines 2431 – 2444 will assign the first element of each detected burst to the variable burstview596 and it will also assign the last element of each detected burst to the variable burstview597. Lines 2447 – 2457 will find the corresponding index matching to the first element of each detected bursts in the variable X2 and store these indices in burstview2. Lines 2461 – 2471 will do the exact same as line 2447 – 2457 but now with the last element of each detected burst and store the indices in burstview3. Lines 2476 – 2481 will subtract 1500 indices from the starting index of each detected burst and store these indices in burstview19. Lines 2484 – 2490 will add 1500 indices to the last index of each detected burst and store these indices in burstview20. Lines 2494 – 2497 is a check to make sure that the indices in burstview20 is not higher than the last index of the whole recording. If it is bigger than we set the index to the last index of the recording.

**Burstiness index (lines 2515 - 2559)**

Lines 2517 – 2530 bins the data with bins in which each bin contains 1/30 of a second. The binned data is stored in bincountssec and will be used to create the video if the user presses the button in the GUI. *The user can alter the number 30 in line 2518 and 2522 to alter the frames per second in the video.* Lines 2534 – 2546 can be ignored as it bins the data using the same bin ranges as in lines 2517 – 2530 however this will be maybe used in the future for creating a different video. Lines 2548 – 2551 concatenates all the spikes times from the variable M2 into one long vector named M3. M3 is sorted in line 2552. Afterwards the data is binned in 1 second bins in line 2553. Line 2554 calculates the how many bins need to be included to be 15%. Line 2556 calculates the burstiness index by using a built-in MATLAB function called maxk which allows us to obtain the maximum values of n numbers of elements. Then we follow the formula in line 2514 to calculate the burstiness index.

**Connectivity maps (lines 2572 - 3868)**

Lines 2572 – 2575 will calculate the 10 channels with the highest spiking activity which will be used for plotting purposes in the GUI. Lines 2580 – 2585 removes any channel with less than 10 spikes. Lines 2588 – 3858 will create a new variable called newM2 depending on the number of channels in the dataset. This variable newM2 based on the variable M2 will be used as a layout to create the connectivity maps. Similarly, to the maps created for the heatmaps we will use nan values to fill in the gaps so that all the rows are equal. In the case of the single-well both the row and columns are 12 elements which the 60 (lines 2589 - 2743) or 120 (lines 3712 - 3857) channels are mapped on. The other lines can be ignored as they are not used anymore here. *However, they were left in for other users to use in case they have a unique MEA layout. If the MEA layout cannot be fit in a 12 x 12 matrix then the user can make it bigger however the user has to make sure that the names assigned to the channels is correct.* Lines 3860 – 3868 is used to create a variable called textstrings which contains the correct positions for the channel names. *If the user has added a different MEA layout then the user can add the correct channel names here. Please ensure that the total length and empty spaces of textstrings matches with newM2.*

**Network bursts (lines 3877 - 4405)**

Depending on which SCB method is chosen in the beginning it will either take the SCB detected by the max interval method, or the log ISI method as can be seen in line 3877 and line 4144. Lines 3882 – 3888 collects all the starting times of all the detected SCBs and stores in one vector in starttimesbursts2. Line 3890 selects the non-overlapping starting times and stores them in uniquestarttimesbursts. Lines 3897 – 3900 takes the detected SCBs in each channel and converts them from cells into one vector per channel and stores it in a temporary cell called temp. Lines 3902 – 3903 takes all the vectors of each channel from the variable temp and puts everything in one matrix called newc. Empty channels or channels that did not contain as many detected SCBs were filled up with nan values. Lines 3908 – 3912 will loop through the uniquestarttimesbursts and create a window around the start times of SCBs. This window is ± 100 ms (default). Then this window will be used to look in the matrix of newc to find if there are SCBs that fall within this window on other channels. if there are other SCBs that fall within this window are found then the channel of these potential network bursts will be stored in channelnumbernnbursts, and the time will be stored in timeofnnbursts. Please keep in mind that the values timeofnnbursts are index values. Lines 3918 – 3928 will check if all results are not all from the same channel. If the SCBs that fall within this are all from the same channel and no other channel, then this potential network burst gets removed. Lines 3939 – 3945 will check if the SCBS in channelnumbernnbursts are from at least 2 distinct channels (default). If this is not the case, then the potential network bursts are removed.

Lines 3948 – 3950 are used to remove empty cells and nan values in the variables uniquestarttimesbursts, channelnumbernnbursts and timeofnnbursts. Lines 3957 – 3962 creates a new variable called burst6length that is the exact same as burst6 except instead of containing the spike times of each detected SCBs, it contains the length of each detected SCBs per channel. Lines 3964 – 3966 converts the cells of burst6length into one vector for each channel. So, each number is the length of each detected SCB in each channel. Lines 3969 – 3983 is used to determine which SCBs belong to the potential network bursts. We do this by checking the index values in the variable timeofnnbursts. These index values were obtained from the matrix newc that contained all the spike timings of the detected SCBs one of after each other. For example, in channel 1 there is a potential network burst. This potential network burst occurs in channel 1 and 2 and the one of the SCB that belongs to this network burst has an index 67 in channel 2. In order to find the correct burst that belongs to this potential network burst we make use of a bult-in MATLAB function called cumsum. Let’s say there are 3 SCBs in channel 2 and they have the length of 20 for the first SCB,30 for the 2^nd^ SCB and 40 for the 3^rd^ SCB. Then using the function cumsum we can find that index 67 is in SCB number 3. We then assign this 3^rd^ SCB to a new variable called potentialnnbursts. Lines 3992 – 3995 calculates the median + 2.5 standard deviation of each channel based on the time values of the potential network bursts. Lines 3997 – 4004 checks if median time value of each SCB that is part of the potential network burst is smaller than the value calculated in lines 3992 – 3995. If it is smaller than then the SCB will be removed as it is an outlier. Lines 4020 – 4026 creates a new variable called potentialnnburstsvector. The first column contains all the SCBs that belong to the potential network burst as one vector. The 2^nd^ column contains the start time and end time for the potential network burst.

So up until now we have found potential network bursts based on the detected SCBs. We used a window around the detected SCBs to determine if there are other SCBs that fall within this window. Then we searched the corresponding bursts that fall within this window. So up until now we only have the beginning parts of the potential network bursts. Lines 4028 – 4038 will find other SCBs that fall within the start and end time listed in the 2^nd^ column of the variable potentialnnburstsvector. For example, we have a potential network burst that starts at 1 second and ends at 3 seconds and it occurs on channel 1 and 2. Up until now we have only found the SCBs that fall within the window of 0.9 – 1.1 seconds. Now we will find the other overlapping SCBs that for example start at 0.5 seconds and end at 3.6 seconds on other channels. We do this by using the start and end time listed in the 2^nd^ column of potentialnnburstsvector and looking through all the detected SCBs channel by channel. If we find values within the other SCBs on other channels that occur within the start and end time of the potential network bursts, we will save this SCB and the channel and the index of the SCB. So, we know later which SCBs to merge with. Lines 4042 – 4046 are used to remove any empty cell from the variables tobemergedbursts, mergeburstchannel, mergeburstnumber. Lines 4051 – 4055 are used to remove potential network bursts if less than 50% of the active channels (default) participate in the potential network burst. Lines 4059 – 4063 creates a new variable called findoverlappingnnbursts and contains the start and end time of the potential burst in one column obtained from the variable tobemergedbursts. This is done because by adding the other overlapping SCBs the end time for the potential network bursts might have changed. Lines 4065 – 4089 uses the start time and end time from findverlappingnbursts to remove duplicate potential network burst and chooses the network burst that ends the latest. The reason for the duplicate potential network burst is because we go through everything channel by channel which is why we sometimes select a similar potential network burst on other channels. For example, a potential network burst is found and channel 1 and 2 contributes to this potential network burst.

Then we will find one potential network burst on channel 1 and another one on channel 2 but, it’s one potential network burst. Lines 4095 – 4098 is to remove any empty cell from the variables tobermergedbursts, mergeburstchannel, mergeburstnumber and findoverlapingnnbursts. Lines 4104 – 4138 are used to calculate the network burst metrics which are explained in table 1 on page 23/24. Lines 4142 – 4405 is the same as lines 3882 – 4138 except instead of using SCBs detected using the max interval method we use the SCBs detected by the log ISI method. Everything else is the same. *The user can add another method to detect network bursts here and can make use of the SCB flag. However, the user must make sure that this new method is added in the GUI of* ***parameters.***

**Conditional firing probability (lines 4415 - 4626)**

Line 4415 will create one vector called spikestimes containing all the spike times. Lines 4416 – 4431 will carte a vector called ‘spikeslocations’ that matches the vector created in line 4415 however instead of containing all the spike times it contains the channel locations of each detected spike. For example, there are 3000 spikes in the first channel and 200 in the 2^nd^ channel. Then the first 3000 elements will be 1’s and from 3001 until 3200 it will be 2’s. Line 4433 sorts the variable spikestimes and we store the index and sorted spikes separately. Line 4434 sorts the variable spikeslocations using the indexes obtained from line 4433. We change the variable names in lines 4437 – 4438 for easier writing. Lines 4440 – 4443 will set the functional connections to zero if there are less than 2^15 detected spikes in the whole dataset and skip the code for detecting functional connections. The reason for this is because we use the CFP method and for this method, we split the data in chunks of 2^15 spikes. Lines 4447 – 4462 divides the spike data in chunks of 2^15 spikes and assigns the first 2^15 spikes to the first chunk and the next 2^15 to the 2^nd^ chunk etc in the variable divdataTs. We also store from which channel each spike is in the variable divdata. Please note that we disregard any spikes that don’t fit in a chunk. For example, there are 2^15 +1 spikes in total. We would have one chunk and one spike leftover. The one spike would be ignored in the CFP calculations. Lines 4467 – 4473 will sort the spike times within each chunk into their corresponding channels. For example, we have 60 channels, and the 1^st^ element of the first chunk is a 2 and the next element is an 18 then the first element will be put in the 2^nd^ cell and the next element will be put in the 18^th^ cell in the new variable sortchunks. As you can see the numbers represents from which channels the spike times come from. So, we sort the spike times of each chunk in their corresponding channels. Lines 4475 – 4477 flips the columns with rows. Lines 4483 – 4523 calculates the CFP curves. We do this by setting up some parameters in lines 4483 – 4487. The important ones are that we use a binsize of 5 ms and the time window we will calculate the CFP curves over will be 500 ms. We calculate the CFP by comparing the spike trains with each other in each chunk separately. Each channel in a chunk will serve as the reference spike train and all other spike trains within this chunk will be compared to this reference spike train. We create the CFP curve by using the spikes of the reference train as the starting point and bin the spike data in bins of 5 ms up until 500 ms. Then we count how many spikes in the other spike train fall within these bins. Then we shift to the next spike in the reference spike train and repeat this process. We end up with a histogram which we normalize by the spikes of the reference spike train as can be seen in lines 4508 – 4513. Since we do this for all the channels, we will also end up with an auto correlation as the channel will also be compared to itself. This auto correlation will be removed in line 4515. We end up with a variable called CFP that contains the CFP curves for each channel per chunk.

In order to extract several parameters from the CFP curves like the amplitude we use a nelder-mead algorithm as described in the manuscript ^1^. In order to do this several parameters are set up in lines 4525 – 4537. Lines 4545 – 4609, specifically lines 4562 - 4566 will calculate the peak value of the CFP curve (M) and the time at which the peak value of the CFP curve occurs (Tt)*.* Lines 4614 – 4620 collects the M values from the different chunks together. For example, the M value of channel 1 compared to channel 2 is 1 in chunk 1 and this M value is 1.2 in chunk 2 and there are only 2 chunks in total then the final M value in variable M is 2.2. Line 4622 divides the M values by the number of chunks to get a mean value. These M values are used to create the connectivity maps. *The user can plot the M values of all the chunks to follow changes in the functional connections over time*

**Save variables and closure of analysis (lines 4630 - 4662)**

Lines 4634 – 4638 creates a new folder called Analzyedfiles to store the newly created mat file that will contain all the variables listed on page 25. Lines 4652 – 4661 will open either the GUI for multiwell data or single-well data.

**8.3 Multiwell_Mainbody.m**

**Extracting voltage trace data and channel information**

Line 4 will set the variable multiwell1 to 1 which ensures that the GUI will recognize the output to be from a multiwell. Lines 6 – 11 will set the variable parts to be either 12 or 24. This variable parts will determine how many parts the multiwell data set is split into. *The user can change this number to match the amount of wells that the multiwell dataset has. Please make sure that the number here matches with the amount of wells that exist in the dataset because otherwise the data will not be properly split. For example, there are 120 channels in total en we have 12 wells with 10 channels in each well. If the variable parts is set at 13 then the 120 channels would be split into 13 parts. This means that certain channels would be placed in the wrong wells during the calculations. This will result in wrong calculations for network bursts and functional connections.* Line 13 is important because this is one of the key differences between the script used for the single-well (**MEAToolboxV3**) and this script used for the multiwell. With line 13 we loop through the divived multiwell data. The reason why we split the multiwell data is because otherwise it would be very difficult to analzye the data as you would need a large amount of ram in order to load all the voltage data from the multiwell. Line 15 will extract the channel names. Line 16 will calculate the amount of wells there are in the dataset. Line 17 will calculate which channels belongs to which part. For example we have 10 wells each with 12 channels for a total of 120 channels. we set the variable parts to 10 then the vairable partznum will be one vector in which the first elemnt is 1, the 2^nd^ element will be 12, the 3^rd^ will be 24 etc. This will allows us in line 24 to select specific wells from the multiwell dataset for the analysis.

**Please note that after these lines all other lines in this script are very similar as the code in MEAToolboxV3. In order to minimize repetition we will refer to the lines in MEA-ToolBox V3.m that matches with the lines of Multiwell_mainBody for the explanantion.**

Lines 26 – 55 matches with lines 188 – 224 (please see page 32 for the explanation)

**Filtering the data (lines 57 – 95)**

Lines 57 – 95 matches with lines 226 – 267 (please see page 32 for the explanation)

**Determining baseline noise (lines 102 – 265)**

Lines 102 – 265 matches with lines 274 – 447 (please see page 33 for the explanation)

**Calculating the Root Mean Square (lines 269 – 308)**

Lines 269 – 308 matches with lines 451 – 490 (please see page 34 for the explanation)

**Spike Detection (lines 316 – 405)**

Lines 316 – 405 matches with lines 498 – 586 (please see page 35 for the explanation)

**Preparation for artefact detection (lines 409 – 504)**

Lines – 409 – 504 matches with lines 591 – 683 (please see page 35 for the explanation)

**Artefact detection (lines 522 – 575)**

Lines 522 – 575 matches with lines 698 – 751 (please see page 36 for the explanation)

**Preparation general data (lines 579 – 601)**

579 – 601 matches with lines 755 – 762 (please see page 36 for the explanation)

**Spike Waveforms (lines 609 – 833)**

Lines 609 – 833 matches with lines 769 – 1000 (please see page 37 for the explanation)

**Preparation for spike raster plots (lines 1211 – 1228)**

Lines 835 – 854 matches with 1211 – 1228 (please see page 37 for the explanation)

**Single**-**channel burst detection (Max interval method) (lines 1241 – 1501) (extra lines)**

Lines 867 – 1133 matches with lines 1241 – 1501 (please see page 37 for the explanation)

**Single**-**channel burst detection (Log ISI method) (lines 1519 – 1894)**

Lines 1151 – 1530 matches with lines 1519 – 1894 (please see page 38 for the explanation)

**General data (lines 1901 – 2166)**

Lines 1537 – 1796 matches with lines 1901 – 2166 (please see page 39 for the explanation)

**Heatmap generation (lines 2169 – 2400)**

Lines 1799 – 2023 matches with lines 2169 – 2400 (please see page 40 for the explanation)

**Active/inactive channels (lines 2406 – 2421)**

Lines 2029 – 2044 matches with lines 2406 – 2421 (please see page 40 for the explanation)

**Zoomed-in single**-**channel bursts (lines 2425 – 2499)**

Lines 2054 – 2128 matches with lines 2425 – 2499 (please see page 40 for the explanation)

**Burstiness index (lines 2515 – 2559)**

Lines 2144 – 2191 matches with lines 2515 – 2559 (please see page 41 for the explanation)

**Connectivity maps (lines 2572 – 3868)**

Lines 2203 – 3288 matches with lines 2572 – 3868 (please see page 41 for the explanation)

**Network bursts (lines 3877 – 4405)**

Lines 3295 – 3843 matches with lines 3877 – 4405 (please see page 41 for the explanation)

**Conditional firing probability (lines 4415 – 4626)**

Lines 3853 – 4065 matches with lines 4415 – 4626 (please see page 43 for the explanation)

**Save variables and closure of analysis (lines 4630 – 4662)**

Lines 4069 – 4114 matches with lines 4630 – 4662 (please see page 44 for the explanation)

**8.4 Justspiketimes.m**

Similar to the **Multiwell_Mainbody.m**, the script justspiketime.m shares a lot of the code with MEAToolboxV3.m the differences lies in where the justspiketimes.m searches for the spike data. The script assumes that the data file is a HDF5 file and will search in specific field names to look for the spike data and spike wavefrom dat. Lines 131 – 148 will look in a specific field in the HDF5 file to search for spike waveforms and store them in Data3. Please make sure that these are voltage values. Lines 154 – 170 creates the variable Data4 which contains all the spike times. The code will try to divide the values here by 1000000 in line 468 to convert the values into the seconds range. Currently, the code is looking for the spike times in the field Data_ts_1 and loop through Data_ts_2, Data_ts_3 etc. to find spike times corresponding to their channels. *The user can change the loop to search in a different field. The most important thing is that the variable Data4 should contain the spike times in a cell for each channel seperately. Also keep in mind that the Data3 should contain the voltage values of the spike waveforms and that the values in Data4 should be spike times either in seconds and you remove the lines 467 – 469 or you multiple the values by 1000000 in Data4.*

**Please note that all other lines in this script are very similar as the code in MEAToolboxV3.m in order to minimize repetition we will refer to the lines in MEAToolboxV3 that matches with the lines of justspiketimes for the explanantion.**

**Verification of the data format (lines 3 – 90)**

Lines 3 – 90 matches with lines 7 – 79 (please see page 32 for the explanation)

**Extracting voltage trace data and channel information (lines 176 – 184)**

Lines 176 – 184 matches with lines 188 – 231 (please see page 32 for the explanation)

Lines 186 – 229 are used to acquire the minimum or maximum value voltage value of the spike waveforms.

**Mapping the channel names (lines 232 – 423)**

Lines 232 – 423 matches with lines 1006 – 1206 (please see page 37 for the explanation)

**Preparation for spike raster plots (lines 1211 - 1228)**

Lines 428 - 477 matches with lines 1211 – 1228 (please see page 37 for the explanation)

**Single**-**channel burst detection (Max interval method) (lines 488 – 753)**

Lines 488 – 753 matches with lines 1241 – 1501 (please see page 37 for the explanation)

**Single**-**channel burst detection (Log ISI method) (lines 773 – 1153)**

Lines 773 – 1153 matches with lines 1519 – 1894 (please see page 38 for the explanation)

**General data (lines 1155 – 1421)**

Lines 1155 – 1421 matches with lines 1901 – 2166 (please see page 39 for the explanation)

**Heatmap generation (lines 1424 – 1654)**

Lines 1424 – 1654 matches with lines 2169 – 2400 (please see page 40 for the explanation)

**Active/inactive channels (lines 1662 – 1674)**

Lines 1662 – 1674 matches with lines 2406 – 2421 (please see page 40 for the explanation)

**Burstiness index (lines 1694 – 1736)**

Lines 1694 – 1736 matches with lines 2515 – 2559 (please see page 41 for the explanation)

**Connectivity maps (lines 1741 – 3037)**

Lines 1741 – 3037 matches with lines 2572 – 3868 (please see page 41 for the explanation)

**Network bursts (lines 3044 – 3577)**

Lines 3044 – 3577 matches with lines 3877 – 4405 (please see page 41 for the explanation)

**Conditional firing probability (lines 3581 – 3793)**

Lines 3581 – 3793 matches with lines 4415 – 4626 (please see page 43 for the explanation)

**Save variables and closure of analysis (lines 4630 – 4662)**

Lines 3799 – 3833 matches with lines 4630 – 4662 (please see page 44 for the explanation)

**8.5 CSVfiles.m**

**Extracting spikes (lines 10 – 35)**

The assumption for CSV files is that they are multiwell data. We split the data to a per well format and perform all the calculations per well and save everything for each well separately like the script **Multiwell_Mainbody.m.** Line 16 will search the correct column where the spike times located in. therefore it is imprortant to make sure that the spike times are located in a column that contains the strings ‘Time’ and no other column has this string. Eventually Data4 will contain the spike times in seconds. The same is done for the spike waveforms in lines 19 - 21. Please ensure that the spike times are in seconds or else lines 22 will some problems later on. Line 26 will search for the correct index of the column that contains the information from which channel each spike is from. We use the strings ‘Electrode’ to search for the correct index. *The user can change the string that Is used to look for the correct index if the file has a different name for where the spike times are located in.*

*The important thing is that Indextime should contain the correct index of the column in which the spike times are located in.* Line 28 will count the total amount of channels using a built-in MATLAB function called unique. Lines 31 – 35 will create a new variable called sortedspikes in which we use the found indices from the previous lines to assign each spike to their corresponding channel.

**Layout (lines 46 – 153)**

Lines 46 – 69 will create a cell called HITS and assign the channel names to each cell in the first column. The loop that is created will create channel names based on the names of multiwell MEAs from axion biosystems. Line 47 contains the names of each channel within a well. Line 48 and 49 are used to create the correct channel names of which an example can be seen in line 50. *The user can change the strings and numbers within these lines to match their channel names if the structure is the same.*

Lines 77 – 85 will reorganize the organisation of the variable sortedspikes to match the organisation of the channels of the variable HITS. For example, the first element of sortedspikes are spikes from the 2^nd^ channel then these spikes will be moved to the 2^nd^ element. Lines 92 – 101 will create a vector with the amount of spike per channel. We assign the number of spikes to the 2^nd^ column of HITS in line 104. Lines 115 – 119 will assign a zero in the 2^nd^ column if there is an empty cell in the 2^nd^ column. Lines 128 – 153 creates a matrix that contains the layout of the multiwell, and we used nan values to fill in the gaps so that the matrix would have equal lengths. We assume that the wells are in a 4 x 4 format.

**Splitting the data (lines 155 – 167)**

Lines 155 – 158 create a variable containing cells that is equal to the number of wells (e.g., 24 wells mean 24 cells). Within each cell are the numbers of the indexes of the channels that belong to the wells. For example, the first well contains the first 16 indexes, the 2^nd^ well contains the indexes 17 until 32 etc. (this is in the case of wells with 16 channels). Lines 160 – 167 will select the wells that will be analysed. Since we loop through the wells, we start from the first well so we will select the spike data from the channels that corresponds to the first well and in the next loop the data corresponding to the 2^nd^ well etc.

**Preparation for the raster plot (lines 172 – 206)**

Lines 172 – 183 are used to create one vector and assign the channel numbers to the spikes that occur. For example, there are 100 spikes that occur in the first channel and 50 in the 2^nd^ channel. The output (ypoints) of these lines is a vector in which the first 100 elements are 1’s and the next 50 elements are 2’s. Lines 189 – 197 creates one vector (xpoints) that contains all the spike timings from each channel sequentially. So, if we take the previous example, the first 100 spike timings will be from channel 1 and the next 50 spike times are from channel 2. Lines 203 – 206 is to create a variable called M2 which is just sorted spikes, but the columns and rows are switched.

**Please note that all other lines in this script are very similar as the code in MEAToolboxV3.m in order to minimize repetition we will refer to the lines in MEAToolboxV3 that matches with the lines of CSVfiles.m for the explanantion.**

**Single**-**channel burst detection (max interval method) (lines 217 – 483)**

Lines 217 – 483 matches with lines 1241 – 1501 (please see page 37 for the explanation)

**Single**-**channel burst detection (log ISI method) (lines 504 – 881)**

Lines 504 – 881 matches with lines 1519 – 1894 (please see page 38 for the explanation)

**General data (lines 886 – 1177)**

Lines 886 – 1177 matches with lines 1901 – 2166 (please see page 39 for the explanation)

**Heatmap generation (lines 1182 – 1204)**

Lines 1182 – 1204 matches with lines 2169 – 2400 (please see page 40 for the explanation)

**Active/inactive channels (lines 1215 – 1227)**

Lines 1215 – 1227 matches with lines 2406 – 2421 (please see page 40 for the explanation)

**Burstiness index (lines 1248 – 1304)**

Lines 1248 – 1304 matches with lines 2515 – 2559 (please see page 41 for the explanation)

**Connectivity maps (lines 1741 – 3037)**

Lines 1310 – 1331 assigns the layout created in lines 128 – 153 to be used for the connectivity maps and empties any channel with less than 10 spikes.

**Network bursts (lines 1338 – 1883)**

Lines 1338 – 1883 matches with lines 3877 – 4405 (please see page 41 for the explanation)

**Conditional firing probability (lines 1893 – 2110)**

Lines 1893 – 2110 matches with lines 4415 – 4626 (please see page 43 for the explanation)

**Save variables and closure of analysis (lines 4630 – 4662)**

Lines 2116 – 2181 matches with lines 4630 – 4662 (please see page 44 for the explanation)

**References**

1. Nick, C. *et al.* DrCell—a software tool for the analysis of cell signals recorded with extracellular microelectrodes. *Signal Proc Int J* **7**, 96–109 (2013).
2. Le Feber, J. *et al.* Conditional firing probabilities in cultured neuronal networks: A stable underlying structure in widely varying spontaneous activity patterns. *J. Neural Eng.* **4**, 54–67 (2007).
3. Kreuz, T., Haas, J. S., Morelli, A., Abarbanel, H. D. I. & Politi, A. Measuring spike train synchrony. *J. Neurosci. Methods* **165**, 151–161 (2007).
